# Genome-wide association study of 29 morphological traits in *Aegilops tauschii*

**DOI:** 10.1038/srep15562

**Published:** 2015-10-27

**Authors:** Yaxi Liu, Lang Wang, Shuangshuang Mao, Kun Liu, Yanli Lu, Jirui Wang, Yuming Wei, Youliang Zheng

**Affiliations:** 1 Triticeae Research Institute, Sichuan Agricultural University, Wenjiang, Chengdu 611130, China; 2 Maize Research Institute, Sichuan Agricultural University, Wenjiang, Chengdu 611130, China

## Abstract

*Aegilops tauschii* is the D-genome progenitor of hexaploid wheat (*Triticum aestivum*). It is considered to be an important source of genetic variation for wheat breeding, and its genome is an invaluable reference for wheat genomics. We conducted a genome-wide association study using 7,185 single nucleotide polymorphism (SNP) markers across 322 diverse accessions of *Ae. tauschii* that were systematically phenotyped for 29 morphological traits in order to identify marker-trait associations and candidate genes, assess genetic diversity, and classify the accessions based on phenotypic data and genotypic comparison. Using the general linear model and mixed linear model, we identified a total of 18 SNPs significantly associated with 10 morphological traits. Systematic search of the flanking sequences of trait-associated SNPs in public databases identified several genes that may be linked to variations in phenotypes. Cluster analysis using phenotypic data grouped accessions into four clusters, while accessions in the same cluster were not from the same *Ae. tauschii* subspecies or from the same area of origin. This work establishes a fundamental research platform for association studies in *Ae. tauschii* and also provides useful information for understanding the genetic mechanism of agronomic traits in wheat.

*Aegilops tauschii* (2n = 2x = 14, DD), the D-genome progenitor of hexaploid wheat (*Triticum aestivum*), is a diploid and self-pollinated plant. The D genome of *Ae. tauschii* and of the hexaploid wheat are closely related because of the recent origin of the latter by hybridisation of *T. durum* and *Ae. tauschii*^1^. *Ae. tauschii* is an important source of genetic variation for wheat breeding, and its genome is an invaluable reference for wheat genomics, as revealed by its utility for studying wheat gene space^2^,^3^. Although *T. aestivum* was originated by hybridisation of *T. turgidum* (AABB) with *Ae. tauschii* (DD)^4^, the participation of the latter in the hexaploidisation of common wheat was very low, and as a result the genetic diversity of hexaploid wheat is less than that of *Ae. tauschii*^5^. Therefore, understanding the genetic diversity and adaptive evolution of *Ae. tauschii* may provide important insights for breeding elite wheat varieties. Additionally, the high genetic variability of morphological traits in *Ae. tauschii* may indicate the presence of several loci or alleles that still remain to be uncovered.

Conventional linkage mapping is the most common approach to detect quantitative trait loci (QTLs), corresponding to complicated traits in plants. However, linkage mapping using bi-parental crosses is able to reveal information on two alleles at a given locus or few loci segregating in the study population. In addition, the resolution of the detected QTLs is poor, ranging from 10 to 30 cM, due to the limited number of recombination events that occur during the development of mapping populations^6^,^7^. Moreover, the development of mapping populations is an expensive and time-consuming process.

The use of single nucleotide polymorphism (SNP) markers, in conjunction with statistical approaches for association mapping (AM), provides dense genome coverage, decreases genotypic errors, and allows the accurate identification of loci^8^. AM, also known as linkage disequilibrium mapping, is the non-random association of alleles at different loci and considered to be a powerful tool for resolving complex trait variation and identifying different loci and novel alleles in natural populations^9^,^10^. AM has been extensively used to identify genes or QTLs in many plant species including *Arabidopsis*^11^, rice^12^, maize^13^,^14^, potato^15^, and wheat^16^,^17^,^18^. Particularly, genome-wide association studies (GWAS), which exploit marker polymorphisms across all chromosomes, have become increasingly popular and powerful, since they have been successfully used in human and animal genetics^19^,^20^. GWAS has been employed to study several important traits in rice^21^, barley^22^, and wheat^23^,^24^.

In the present study, we used the GWAS approach employing 7,185 SNP markers in a core collection of 322 *Ae. tauschii* accessions of diverse origin in order to: 1) investigate marker-trait associations for 29 morphological traits and 2) scan for candidate genes that control corresponding morphological traits. Furthermore, we aimed to provide a comprehensive overview on genetic diversity of morphological traits, as well as, subspecies classification based on phenotypic data and genotypic comparison. Overall, this study was designed to provide useful information for understanding the genetic mechanism of morphological traits in *Ae. tauschii* and further unlock the regulatory network of complicated morphological traits in this species.

## Results

### Phenotypic evaluation

Analysis of variance (ANOVA) revealed significant variation among genotypes for all 29 morphological traits (Table 1). The level of variation was also reflected by the distribution of traits in 2012 and 2013 (see Supplementary Fig. S1 1a to 29a available online). Significant differences (*P* < 0.001) were observed between 2012 and 2013 for all traits, except for AL2 and GL2. Also, significant variation (*P* < 0.001) and significant differences (*P* < 0.001) in year × genotype interactions were observed for all traits.

Phenotypic variation among genotypes for each trait was confirmed by its range, mean, standard deviation, and coefficient of variation (Table 2). The coefficient of variation in 2012 ranged from 8.08 to 22.19%, while in 2013 it ranged from 7.53 to 23.05%. Compared to 2012, the mean values of SL, IL1, IL2, IL3, IL4, SNN, and AL1 were significantly increased (*P* < 0.001) in 2013, while the rest 22 morphological traits including PH, FL, FW, LN, SPL, SPW, SPN, GL1, GW1, GW2, GT1, GT2, LL1, LL2, LW1, LW2, PL1, PL2, PW1, and PW2 were significantly decreased (*P* < 0.001) in 2013.

Broad-sense heritability estimates were calculated for all 29 morphological traits (see Supplementary Table S1). Among them, GW1 and GW2 had the highest heritability (0.94), while LN had the lowest heritability (0.27). Heritability estimates for all other traits ranged from 0.58 (PH) to 0.90 (SPW).

Pearson’s correlation was used to investigate the relationship of traits between 2012 and 2013, and all of them were significantly correlated (*P* < 0.01) between the two years (see Supplementary Table S2). In 2012 and 2013, GW1 and GW2 were highly correlated (*r* = 0.891^**^, *P* < 0.01 both years), while all other traits were moderately correlated. The best linear unbiased predictor (BLUP) was calculated from the fixed effects of phenotypic data to avail unbiased mean estimates and used in the correlation analysis among the study traits. The correlation coefficients of this combined analysis are presented in Supplementary Table S3. GW1 and GW2 were highly positively correlated (*r* = 0.989^**^, *P* < 0.01), while IL4 and SPN were highly negatively correlated (*r* = −0.589^**^, *P* < 0.01). AL2, SPW, GT1, GT2, LW1, LW2, PL2, PW1, and PW2 were significantly correlated (P < 0.05) with all other traits. The remaining traits showed moderate to weak correlation among each other.

### *Ae. tauschii* classification based on BLUP values

**Figure 1:**
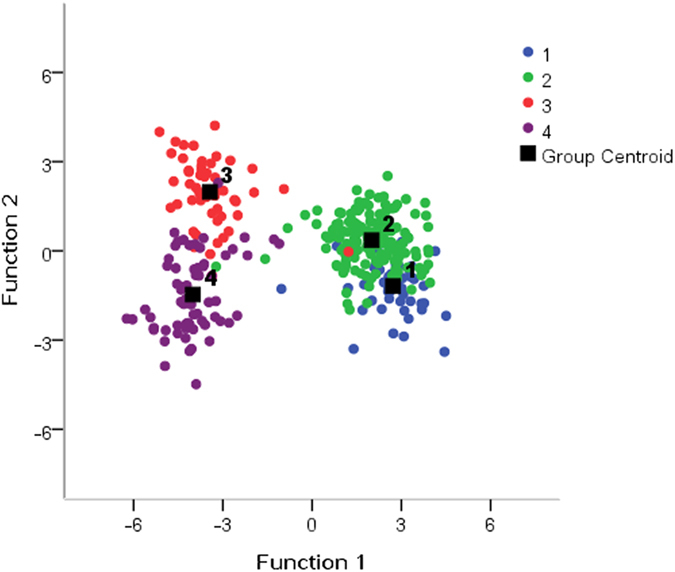
Scatter plots for function 1 and function 2 in discriminant function analysis. 1, *Aegilops tauschii* ssp. *taschii*_I; 2, *Aegilops tauschii* ssp. *taschii*_II; 3, *Aegilops tauschii* ssp. *strangulata*_I; 4, *Aegilops tauschii* ssp. *strangulata*_II.

Discriminant function analysis (Fisher’s method) based on BLUP values was used to show the distance of four *Ae. tauschii* subspecies (*Ae. tauschii* ssp. *tauschii_I, Ae. tauschii* ssp. *tauschii_II, Ae. tauschii* ssp. *strangulata_I*, and *Ae. tauschii* ssp. *strangulata_II*). The results were concordant with those of genotypic comparison (see Supplementary Table S4) for 291 out of 322 accessions, and as a result, a large proportion (90.4%) of *Ae. tauschii* accessions was classified correctly (Fig. 1 and Supplementary Table S5).

Cluster analysis (Ward’s method) was performed using the squared Euclidean distance matrix also based on BLUP values, and all accessions were divided into four clusters (see Supplementary Table S6 and S7). Cluster I included 113 accessions from 13 different areas of origin and 3 different subspecies, while the most frequent subspecies type was *Ae. tauschii* ssp. *tauschii_II*. Cluster II included 78 accessions from 16 different areas of origin and 4 different subspecies; the most frequent subspecies was *Ae. tauschii* ssp. *tauschii_I*. Clusters III and IV comprised 44 and 87 accessions, respectively, that originated from 3 (Cluster III) and 13 (Cluster IV) different areas. Cluster III included 3 and Cluster IV included 4 different subspecies, whereas the most frequent subspecies was *Ae. tauschii* ssp. *strangulata_II* in Cluster III and *Ae. tauschii* ssp. *strangulata_I* in Cluster IV (see Supplementary Table S6).

It was observed that *Ae. tauschii* accessions from different areas of origin were grouped in the same cluster, while accessions from the same area of origin were grouped into different clusters. For instance, all the accessions from Iran (65 accessions) were grouped into four clusters, suggesting the high levels of genetic diversity in each centre of origin. We also observed that Cluster I had a closer relationship with Cluster II, and the main subspecies in both clusters was *Ae. tauschii* ssp. *tauschii*. Cluster III had a closer relationship with Cluster IV, and the main subspecies in both clusters was *Ae. tauschii* ssp*. strangulata* (see Supplementary Table S7). Overall, this analysis showed that there was no relationship between the morphological traits and the centres of origin, revealing high levels of genetic diversity among the accessions.

### Marker-trait association analysis

The Bonferroni-corrected threshold (-lg*p* > 3.84) was used as a cut-off to identify marker-trait associations (MTAs). Using the mean phenotypic values from 2012 and 2013, 12,444 significant SNPs were detected by the GLM and 28 significant SNPs by the MLM (Table 3). Of these, 18 SNPs were detected by both methods. The GLM detected significantly more markers than the MLM because it is much less stringent, as shown by the quantile-quantile (Q–Q) plot (see Supplementary Fig. S1 1e to 29e, 1h to 29h).

In 2012, the GLM detected significant SNPs for all traits, while the MLM for only six traits (SL, AL1, SPN, GW1, GW2, and PW2). The average *r*^*2*^ values that ranged from 2.04 to 9.35% provided an estimate of phenotypic variation explained by SNPs. In 2013, the GLM detected significant SNPs for all traits, while the MLM only for 13 traits (IL1, FL, FW, SNN, SPL, GL2, GW1, GW2, GT1, GT2, LL2, LW1, and LW2). The average *r*^*2*^ values ranged from 2.74 to 7.09% (Table 3).

We also detected associations between SNPs and BLUP values (see Supplementary Fig. S2). A total of 7,809 significant SNPs were detected by the GLM and 10 significant SNPs by the MLM. Only six significant SNPs were detected by both methods. The *r*^*2*^ values ranged from 2.05 to 8.65% (see Supplementary Table S8).

### Significant loci and putative candidate gene

In this study, a total of 15 significant SNPs associated with 10 traits and 21 putative genes were identified (Table 4). Of these SNPs, four were located on chromosome (chr) 2D, two on chr 3D, five on chr 4D, two on chr 5D, one on chr 6D, and one on chr 7D. Location information for four significant SNPs inferred from the genetic map constructed by Luo *et al.*^3^ differed from that inferred as the best hit from the International Wheat Genome Sequencing Consortium (IWGSC). SNP markers *GBQ4KXB02HJM7P_431, contig11810_520, GBUVHFX01CI5PL_126*, and *GBF1XID02IP0NJ_181* were located on chr 2D, 3D, 6D, and 7D, respectively, according to the genetic map, and on chr 2BL, 3B, 6BS, and 7AS, respectively, according to the best hit from the IWGSC (Table 4).

The study traits in 2013 were associated with a higher number of significant SNPs than those in 2012, and only GW1 was associated with significant SNPs in both years. Based on BLUP values, only GW1 and GW2 were associated with 3 significant SNPs each, and a total of seven candidate genes (*116F2, 115G1, 1J9.1, 1J9.2, Rht-D1b, Ig1,* and *TSAlike*) were identified (Table 4).

In 2012, SPN was associated with the highest number of significant SNPs, and a total of four candidate genes (*LR34, cytochrome P450, glutathione-S-transferase 2*, and *glutathione-S-transferase 1*) were identified. In 2013, SNN was associated with the highest number of significant SNPs, and a total of five candidate genes (*LR34, cytochrome P450, ZCCT2, ZCCT1*, and *SNF2P*) were identified. GT1 and GT2 were associated with the same number of significant SNPs, and the same candidate gene (*CKX2.5*) was identified. Similarly, GW1 and GW2 were also associated with the same number of significant SNPs and two candidate genes (*116F2* and *115G1*) were identified (Table 4).

### Pleiotropy and multigenic effect revealed by GWAS

Significant association of the same SNPs with multiple traits might be the result of pleiotropy. We observed that a SNP at 55.616 cM on chr 2D, a SNP at 132.198 cM on chr 4D, and a SNP at 151.266 cM on chr 5D were significantly associated with both GW1 and GW2. Also, a SNP at 113.167 cM on chr 5D was significantly associated with both GT1 and GT2 (Table 4). These associations were also supported by Pearson’s correlation analysis based on BLUP values (see Supplementary Table S3, *r* = 0.988^**^ for GW1 and GW2; *r* = 0.975^**^ for GT1 and GT2; *P* < 0.01).

Furthermore, several different SNPs were significantly associated with the same trait. SNP markers *GBQ4KXB02HJM7P_431, GDEEGVY02FLOCP_398*, and *GDRF1KQ02F8V30_278* were significantly associated with GW1 and GW2. SNP markers *GBB4FNX02JQNSU_161* and *GBUVHFX01CI5PL_126* were significantly associated with SNN and SNP markers *contig15239_471, contig11810_520, GCE8AKX01ALM0H_152*, and *F5MV3MU01BU5XD_286* were significantly associated with SPN (Table 4). These results suggested that some morphological traits were not controlled by a single gene but were quantitative.

## Discussion

Hexaploid wheat (*T. aestivum*) originated by the hybridisation of *T. turgidum* (AABB) with *Ae. tauschii* (DD)^4^, a cross that most probably occurred south or west of the Caspian Sea^4^,^25^. The distribution centre of *Ae. tauschii* is along the southern shores of the Caspian Sea and in Azerbaijan, and this species has mainly spread eastwards from the centre of origin^26^, probably due to its diverse adaptability. The study of genetic diversity in *Ae. tauschii* collections may help us in transferring desirable traits to common wheat.

In this study, *Ae. tauschii* accessions showed significant (*P* < 0.001) levels of diversity, as revealed by ANOVA of all morphological traits, and the majority of traits were highly inheritable, showing a broad variation among the accessions. It is well-known that the genotype, environment, and their interaction play an important role in morphological traits. In the two different years, the means of some morphological traits were significantly higher or lower, suggesting that traits were probably affected much more strongly by environmental factors (i.e. year) than by genotype. Discriminant function analysis based on BLUP values was not congruent with the classification based on the genotypic comparison or the areas of origin^27^, and 9.6% of the accessions were misclassified. Cluster analysis using BLUP values grouped accessions into four clusters, while accessions in the same cluster were not from the same subspecies^28^ or from the same area of origin. In each cluster, *Ae. tauschii* ssp. *tauschii* and ssp. *strangulata* did not separate from each other entirely. Cluster I included 110 accessions from *Ae. tauschii* ssp. *tauschii* against 3 from ssp. *Strangulate*. Cluster II included 66 accessions from *Ae. tauschii* ssp. *tauschii* versus 12 from ssp. *Strangulate*. Cluster III and Cluster IV were 1 VS 43 and 27 VS 60 respectively. The intermediate forms and hybrids between the two subspecies reported by Kihara *et al.*^29^ reveal possible events of migration that probably led to a decrease in genetic differentiation and may explain the results of this study. Jaaska^30^ reported that *Ae. tauschii* ssp. *tauschii* and ssp. *strangulata* were not closely related to each other; however, it seems that intraspecies branching outs probably occurred at the same time with big changes in the genetic structure of *Ae. tauschii* collections.

We identified many significant SNPs and related candidate genes associated with morphological traits in *Ae. tauschii* by employing the GWAS approach. It is known that linkage mapping can also detect QTLs using different segregating populations tested in different environments. Although, few QTLs have been identified in *Ae. tauschii*, a large number of QTLs related to agronomical traits have been identified in common wheat by the conventional mapping approach. Since the D-genome of *Ae. tauschii* and of common wheat are homologous, the identification of QTLs in *Ae. tauschii* by the GWAS approach may offer useful information for understanding the genetic mechanism of agronomic traits in wheat too.

In the present study, significant SNPs identified by the GLM or the MLM were distributed on chr 2D–7D. We identified a locus related to SL on chr 4D that was also reported by Sourdille *et al.*^31^, but they found that it was located in *Xgwm261* of chr 2DS. We identified four loci related to SPN on chr 2D, 3D, and 4D. These results were partially consistent with those of McCartney *et al.*^32^, who reported a QTL related to SPN on chr 4DL between *Xbarc48* and *Xgwm194* and with those of Rasheed *et al.*^33^ who reported a genomic region related to horizontal principal component 4 (HPC4) trait on 3D (53.86 cM) in synthetic hexaploid wheat. The physical maps of wheat and *Ae. tauschii* are yet to be finished, and therefore little information on chromosome locations can be provided. Hence, the loci identified herein as being associated with morphological traits cannot be directly compared with QTL reported by other researchers.

Previous genetic research has uncovered many genes that affect important agronomic traits, but only a few have been practically used in plant breeding. For instance, it is known that numerous *Rht* (reduced height) genes affect plant height; however, only the *Rht-B1b (Rht1), Rht-D1b (Rht2)*, and *Rht8c* have been used extensively in agriculture^34^. Other important agronomic genes include the *Ppd* (response to photoperiod) genes, the *Vrn* (response to vernalisation) genes, and the *Eps* (earliness per se) genes^35^. These genes play a vital role in the processes involved inplant growth and they are homologous between different plant species. In this study, we identified a few candidate genes associated with phenotypic traits. These genes are partially homologous to *Hordeum vulgare* and *Zea mays* and highly homologous to different species of *Triticum* or *Aegilops*. These genes included enzyme genes, such as *Acc-2, CKX2.5, Ig1, TSAlike, Acc-1, BAM3, LR34, cytochrome P450, glutathione-S-transferase 2,* and *glutathione-S-transferase 1*; hormone response genes, such as *Aux/IAA* gene family and *ERF1*; regulatory element genes, such as *1J9.1* and *1J9.2*; and other genes, such as *AXAH3, 116F2, 115G1, Rht-D1b, ZCCT2, ZCCT1,* and *SNF2P*. Additionally, the semi-dwarfing gene *Rht-D1b* was identified to affect GW2 in our study, indicating that it may probably control more than one agronomic trait. Pleiotropic and multigenic effects were also observed in this study, such as SNPs *GBQ4KXB02HJM7P_431* (at 55.616 cM on chr 2D), *GDEEGVY02FLOCP_398* (at 132.198 cM on chr 4D),) and *GDRF1KQ02F8V30_278* (at 151.266 cM on chr 5D) that are associated with both GW1 and GW2 traits; SNP *GBB4FNX01BXV4Q_52* (at 113.167 cM on chr 5D) is associated with both GT1 and GT2 traits. Pleiotropic or closely linked genes^36^ allowed us to unravel the origin of genetic correlations among the morphological traits, while multigenic effects revealed that the traits of *Ae. tauschii* were complex and affected by polygenes.

Our findings are a tool that can assist in genetic dissection of D genome of bread wheat. When the desired gene resides in D genome of *Ae. tauschii*, homologous pairing is expected between the donor and the recipient bread wheat chromosome (of the D genome) and no pairing induction is required. The most effective way is that skilful use of tetraploid wheat, which has AB genome, is hybridised with *Ae. tauschii*, and the chromosomes of the F_1_ hybrid are doubled using colchicine treatment. The product is a fertile synthetic hexaploid ABD genotype fully homologous to bread wheat. Such wheat–*Ae. tauschii* hybrids have a high level of sterility. Homologous chromosomes of the hexaploid wheat will readily recombine in a hybrid, and such synthetic lines can serve as a gene pool derived from *Ae. tauschii* that is ready for screening for any desired trait and allow for an easy transfer of responsible genes^37^.

In summary, we performed genome-wide association studies for morphological traits in a population containing 322 *Ae. tauschii* accessions using 7,185 polymorphic SNP markers. Fifteen significant markers were detected by both GLM and MLM. At significant loci and flanking regions, we identified candidate genes for morphological traits including enzyme genes, hormone response genes, and other genes that may affect morphological traits. Additionally, discriminant function analysis and cluster analysis showed that there was no correlation between the morphological traits and the centres of origin and revealed high levels of genetic diversity among the tested populations. The identified SNPs and genes offer essential knowledge for cloning genes related to morphological traits in *Ae. tauschii* and wheat. These findings provide useful information for further unlocking of genetic mechanism of morphological traits in *Ae. tauschii*, followed by agronomic traits in wheat.

## Methods

### Plant Material

A total of 322 *A. tauschii* accessions used in this study were obtained from the Triticeae Research Institute of Sichuan Agricultural University (SAU). Detailed information for each accession is given in Supplementary Table S9 available online.

### Phenotypic evaluation

*Ae. tauschii* accessions were evaluated in the field at Wenjiang, Chengdu, China, during the growing season (April–June) in 2012 and 2013. All accessions were grown during the planting season (October 2011 and 2012). Every accession was planted in three rows, each row with five plants, the length of each row was 1.5 m, and spacing between plants and between rows was 0.3 m. Thus, each accession comprised 15 plants from three replications, and each replication contained five plants. Those 15 plants of each accession were selected to investigating morphological traits. A total of 29 morphological traits were investigated including plant height (PH), spike length (SL), internode length 1 (IL1), internode length 2 (IL2), internode length 3 (IL3), internode length 4 (IL4), flag leaf length (FL), flag leaf width (FW), leaf number (LN), stem node number (SNN), awn length 1 (AL1), awn length 2 (AL2), spikelet length (SPL), spikelet width (SPW), spikelet number (SPN), glume length 1 (GL1), glume length 2 (GL2), glume width 1 (GW1), glume width 2 (GW2), glume thickness 1 (GT1), glume thickness 2 (GT2), lemma length 1 (LL1), lemma length 2 (LL2), lemma width 1 (LW1), lemma width 2 (LW2), palea length 1 (PL1), palea length 2 (PL2), palea width 1 (PW1), and palea width 2 (PW2). Measurements were conducted using a straightedge and a Vernier calliper. A brief description of each trait is summarised in Supplementary Table S10 available online.

Descriptive statistics, ANOVA, correlation analysis, and heritability estimates of all traits were conducted in SAS 9.2 (SAS Institute Inc., Cary, NC) using 2-year data. Broad-sense heritability was defined as H = V_G_/(V_G_ + V_E_), where V_G_ and V_E_ are the estimates of genetic and environmental variance, respectively^38^. Phenotypic BLUP was estimated taking into account the genotype by environment interaction^39^ and was used to perform correlation analysis, discriminant function analysis, cluster analysis, and further association analysis. Discriminant function analysis (Fisher’s method^40^) and cluster analysis (Ward’s method^41^) were conducted using SPSS 20.0 (IBM Corp., Armonk, NY).

### 10K Infinium iSelect SNP array and SNP genotyping

A total of 7,185 polymorphic SNP markers in the array were uniquely mapped on the genetic map and the physical map of *Ae. tauschii* built from bacterial artificial chromosome clones^3^. SNPs were assayed according to the manufacturer’s protocol (Illumina Inc., San Diego, CA) at the Genome Centre, University of California, Davis, USA. Normalised Cy3 and Cy5 fluorescence intensities for each DNA sample were graphed using GenomeStudio software (Illumina Inc., San Diego, CA), resulting in genotype clustering for each SNP marker. Detailed information on SNP genotyping of *Ae. tauschii* accessions has been described in our previous study^28^.

### Population structure

The Bayesian inference program STRUCTURE 2.3.3^42^,^43^ was used to assess population structure using a set of 7,185 polymorphic SNP markers mapped on the genetic map of *Ae. tauschii*^3^. We used the linkage ancestry model and the allele frequency correlated model. A total of 100 burn-in iterations followed by 100 Markov Chain Monte Carlo (MCMC) iterations for *K* = 1–10 clusters were used to identify the optimal range of *K*. For each *K*, five independent runs were produced. The optimal value of *K* was determined using the delta *K* method^44^. Here, *K* = 4 was used, and the whole panel was divided into Subpopulation (Subp) 1 (*Ae. tauschii* ssp. *taschii*_I), Subp 2 (*Ae. tauschii* ssp. *taschii*_II), Subp 3 (*Ae. tauschii* ssp. *strangulata*_I), and Subp 4 (*Ae. tauschii* ssp. *strangulata*_I) based on our previous study^28^.

### Marker-trait associations

MTAs of 6,905 SNP markers with minor allele frequency (MAF) > 0.05 were evaluated based on the phenotypic mean data from 2012 and 2013, as well as BLUP values using Tassel 2.1^41^,^42^ Two models, 1) GLM adjusted using the Q-matrix and 2) the MLM adjusted using both the Q- and kinship (K)-matrix were employed to reduce errors from population structure. Bonferroni-corrected thresholds at α = 1 were used as cut-offs. When the number of markers was 6,905 SNPs at α = 1, the Bonferroni-corrected threshold for the p value was 144.823 × 10^–6^ with a corresponding –log_10_(*p*) value of 3.84. Significant markers were demonstrated with a Manhattan plot generated in R 3.03 (http://www.r-project.org/). Significant p-values (observed p-values against cumulative p-values in a negative log_10_ scale) were demonstrated with a Q–Q plot also generated in R.

### Putative candidate genes analysis

Putative candidate genes were proposed for each significant MTA using the corresponding extending SNP marker sequence from the National Centre for Biotechnology Information (NCBI, http://www.ncbi.nlm.nih.gov/) GenBank non-redundant database, and the extending SNP marker sequence derived from a 5-kb increase around each SNP marker that was performed by BLAST in the IWGSC (http://www.wheatgenome.org/).

## Additional Information

**How to cite this article**: Liu, Y. *et al.* Genome-wide association study of 29 morphological traits in *Aegilops tauschii*. *Sci. Rep.* **5**, 15562; doi: 10.1038/srep15562 (2015).

## Supplementary Material

Supplementary Information Table S3

Supplementary Information Table S5

Supplementary Information Table S7

Supplementary Information Table S8

Supplementary Information Table S9

Supplementary Information Table S10

Supplementary Information

## Figures and Tables

**Table 1:** Analysis of variance for the tested 29 morphological traits in year 2012 and 2013.

Variables	Type III sum of square			Mean square			F value			P value		
	Year	Genotype	Year × Genotype	Year	Genotype	Year × Genotype	Year	Genotype	Year × Genotype	Year	Genotype	Year × Genotype
df	1	359	359	1	359	359	1	359	359	1	321	321
PH	185213.27	371880.14	151193.06	185213.27	1158.51	471.01	5635.15	35.25	14.33	***	***	***
SL	55.50	6896.99	2347.92	55.50	21.49	7.31	55.58	21.51	7.32	***	***	***
IL1	44.10	3167.07	537.73	44.10	9.87	1.68	146.29	32.73	5.56	***	***	***
IL2	16.79	2720.03	348.10	16.79	8.47	1.08	78.34	39.55	5.06	***	***	***
IL3	26.77	2541.50	348.25	26.77	7.92	1.08	139.61	41.30	5.66	***	***	***
IL4	47.35	2334.23	376.34	47.35	7.27	1.17	237.01	36.40	5.87	***	***	***
FL	3457.23	18897.64	7028.47	3457.23	58.87	21.90	907.35	15.45	5.75	***	***	***
FW	295.05	3531.32	1194.05	295.05	11.00	3.72	617.32	23.02	7.78	***	***	***
LN	61.92	1224.84	879.35	61.92	3.82	2.74	243.28	14.99	10.76	***	***	***
SNN	11.60	1060.50	333.89	11.60	3.30	1.04	56.27	16.03	5.05	***	***	***
AL1	383.77	143047.12	35647.56	383.77	445.63	111.05	25.54	29.65	7.39	***	***	***
AL2	0.74	128910.61	30065.93	0.74	401.59	93.66	0.05	29.71	6.93	ns	***	***
SPL	4.57	1137.68	189.90	4.57	3.54	0.59	35.42	27.46	4.58	***	***	***
SPW	19.51	1234.97	108.13	19.51	3.85	0.34	473.60	93.39	8.18	***	***	***
SPN	333.11	10444.88	1291.79	333.11	32.54	4.02	517.19	50.52	6.25	***	***	***
GL1	0.70	713.23	99.04	0.70	2.22	0.31	11.20	35.64	4.95	***	***	***
GL2	0.13	683.21	103.22	0.13	2.13	0.32	2.04	33.08	5.00	ns	***	***
GW1	41.94	1267.02	62.95	41.94	3.95	0.20	1251.11	117.76	5.85	***	***	***
GW2	16.80	1295.54	66.48	16.80	4.04	0.21	492.42	118.32	6.07	***	***	***
GT1	5.87	177.99	27.32	5.87	0.55	0.09	519.23	49.02	7.52	***	***	***
GT2	3.00	160.23	23.01	3.00	0.50	0.07	245.23	40.81	5.86	***	***	***
LL1	18.45	1028.93	161.55	18.45	3.21	0.50	182.00	31.62	4.97	***	***	***
LL2	1.96	1138.84	261.77	1.96	3.55	0.82	18.58	33.62	7.73	***	***	***
LW1	26.76	580.20	59.37	26.76	1.81	0.18	1006.51	67.97	6.96	***	***	***
LW2	5.95	569.31	56.83	5.95	1.77	0.18	222.64	66.37	6.62	***	***	***
PL1	48.39	1383.29	155.25	48.39	4.31	0.48	487.30	43.39	4.87	***	***	***
PL2	19.41	1401.72	265.74	19.41	4.37	0.83	203.54	45.78	8.68	***	***	***
PW1	22.42	513.86	71.81	22.42	1.60	0.22	529.50	37.80	5.28	***	***	***
PW2	6.56	474.20	72.67	6.56	1.48	0.23	204.07	45.92	7.04	***	***	***

df: degree of freedom; PH: plant height; SL: spike length; IL1: internode length1; IL2: internode length 2; IL3: internode length 3; IL4: internode length 4; FL: flag leaf length; FW: flag leaf width; LN: leaf numbers; SNN: stem node numbers; AL1: awn length1; AL2: awn length 2; SPL: spikelet length; SPW: spikelet width; SPN: spikelet numbers; GL1: glume length1; GL2: glume length 2; GW1: glume width 1; GW2: glume width 2; GT1: glume thickness 1; GT2: glume thickness 2; LL1: lemma length 1; LL2: lemma length 2; LW1: lemma width 1; LW2: lemma width 2; PL1: palea length 1; PL2: palea length 2; PW1: palea width 1; PW2: palea width 2. *, ** and *** significant at *P* < 0.05, *P* < 0.01 and *P* < 0.001, respectively; NS: not significant.

**Table 2:** Range, mean, standard deviation (Sd), coefficients of variation (CV%) for the tested 29 morphological traits in year 2012 and 2013.

Variables	Range		Mean		Sd		CV (%)	
	2012	2013	2012	2013	2012	2013	2012	2013
PH	39.56–115.88	32.28–89.46	73.70	57.03	14.87	11.84	20.17	20.75
SL	8.02–17.90	8.40–21.28	13.24	13.53	1.59	1.99	11.99	14.73
IL1	7.00–13.59	5.85–14.69	9.88	10.14	1.08	1.23	10.89	12.11
IL2	6.90–12.50	6.14–12.01	9.16	9.33	1.02	1.08	11.11	11.63
IL3	6.94–12.05	5.77–11.74	8.95	9.15	0.97	1.07	10.82	11.64
IL4	6.84–12.02	5.87–11.68	8.83	9.10	0.92	1.04	10.47	11.47
FL	6.02–24.46	5.97–24.30	15.65	13.32	2.99	3.07	19.10	23.05
FW	6.03–13.00	5.70–11.50	9.24	8.56	1.39	1.18	15.03	13.80
LN	4.00–7.40	3.00–7.80	5.65	5.35	0.70	0.98	12.41	18.34
SNN	3.00–6.40	3.00–6.80	4.66	4.80	0.58	0.81	12.51	16.81
AL1	11.80–57.29	16.00–60.80	35.69	36.42	7.92	8.10	22.19	22.25
AL2	15.01–58.67	8.36–65.00	36.94	36.89	7.25	8.05	19.64	21.81
SPL	5.95–10.46	6.14–10.73	8.08	8.00	0.68	0.69	8.37	8.58
SPW	2.80–5.81	2.59–5.76	4.04	3.87	0.69	0.67	16.99	17.43
SPN	9.40–18.67	7.50–18.2	14.30	13.59	2.09	1.97	14.62	14.47
GL1	5.14–8.45	5.02–8.22	6.63	6.60	0.55	0.52	8.22	7.80
GL2	4.75–8.49	5.13–8.06	6.70	6.69	0.54	0.50	8.08	7.53
GW1	2.56–5.63	2.23–5.42	3.77	3.51	0.68	0.67	17.98	18.99
GW2	2.57–5.71	2.24–5.36	3.71	3.55	0.67	0.68	18.21	19.30
GT1	0.88–2.21	0.76–2.07	1.41	1.31	0.27	0.27	18.93	20.31
GT2	0.78–2.19	0.78–1.97	1.34	1.27	0.25	0.25	18.65	19.85
LL1	5.49–8.97	5.53–9.23	7.26	7.10	0.64	0.64	8.83	9.05
LL2	5.40–9.88	5.73–9.70	7.42	7.37	0.73	0.66	9.86	9.01
LW1	2.13–4.36	1.99–4.17	3.16	2.96	0.48	0.46	15.06	15.72
LW2	2.13–4.32	2.00–4.14	3.05	2.96	0.46	0.47	15.03	15.94
PL1	5.44–9.47	4.88–9.21	7.09	6.82	0.73	0.73	10.29	10.67
PL2	5.49–10.00	5.41–9.65	7.22	7.05	0.77	0.75	10.66	10.69
PW1	1.93–3.83	1.70–3.81	2.79	2.61	0.44	0.45	15.90	17.39
PW2	1.67–3.84	1.67–3.69	2.70	2.60	0.42	0.44	15.61	17.07

PH: plant height; SL: spike length; IL1: internode length1; IL2: internode length 2; IL3: internode length 3; IL4: internode length 4; FL: flag leaf length; FW: flag leaf width; LN: leaf numbers; SNN: stem node numbers; AL1: awn length1; AL2: awn length 2; SPL: spikelet length; SPW: spikelet width; SPN: spikelet numbers; GL1: glume length1; GL2: glume length 2; GW1: glume width 1; GW2: glume width 2; GT1: glume thickness 1; GT2: glume thickness 2; LL1: lemma length 1; LL2: lemma length 2; LW1: lemma width 1; LW2: lemma width 2; PL1: palea length 1; PL2: palea length 2; PW1: palea width 1; PW2: palea width 2.

**Table 3:** GWAS of the investigated 29 morphological traits in years 2012 and 2013 detected by GLM and MLM.

Year	Trait	GLM					MLM					No.Shared^c^
		No.sig^a^	Average -lg(*P*)	Range -lg(*P*)	Average R^2^ (%)^b^	Range R^2^ (%)^b^	No.sig^a^	Average -lg(*P*)	Range -lg(*P*)	Average R^2^ (%)^a^	Range R^2^ (%)^b^	
2012	PH	306	5.29	4.00–11.23	5.13	3.71–11.03						
	SL	33	5.06	4.02–7.49	4.67	3.60–7.13	1	4.12		5.05		1
	IL1	175	5.83	4.00–14.79	6.68	4.40–17.01						
	IL2	146	5.78	4.02–12.81	5.23	3.48–11.72						
	IL3	83	5.27	4.01–9.95	4.41	3.25–8.54						
	IL4	49	4.81	4.01–7.55	3.91	3.17–6.28						
	FL	166	5.40	4.01–6.74	5.45	3.79–7.81						
	FW	386	7.04	4.01–14.93	6.51	3.46–13.54						
	LN	12	4.74	4.02–6.06	4.26	3.55–5.56						
	SNN	11	4.81	4.15–5.96	3.68	3.12–4.64						
	AL1	332	8.33	4.02–13.10	9.35	4.21–18.45	2	4.02	4.01–4.04	4.87	4.81–4.92	
	AL2	240	6.03	4.00–12.14	6.93	4.34–13.92						
	SPL	154	5.45	4.00–9.90	5.20	3.70–9.65						
	SPW	696	7.21	4.02–28.90	4.11	1.96–15.45						
	SPN	20	4.62	4.01–5.76	2.04	1.75–2.60	4	4.30	4.03–4.94	5.29	4.89–6.21	4
	GL1	172	5.50	4.00–10.01	5.47	3.83–10.20						
	GL2	166	5.72	4.01–10.71	5.93	3.99–11.44						
	GW1	644	9.51	4.00–36.14	6.23	2.39–21.43	4	4.46	4.21–5.03	5.46	5.11–6.23	3
	GW2	656	10.19	4.01–39.01	6.65	2.47–22.76	1	4.02		4.80		1
	GT1	389	6.61	4.00–19.21	3.77	2.18–10.84						
	GT2	421	6.88	4.00–20.14	3.69	2.02–10.64						
	LL1	143	5.33	4.01–9.13	4.70	3.42–8.35						
	LL2	143	5.34	4.00–9.32	5.17	3.72–9.25						
	LW1	537	8.16	4.00–29.06	5.04	2.36–16.85						
	LW2	465	7.32	4.00–22.43	4.22	2.19–12.56						
	PL1	265	5.84	4.00–14.58	3.98	2.61–10.01						
	PL2	156	5.71	4.02–12.01	4.88	3.30–10.44						
	PW1	516	7.94	4.01–26.46	4.73	2.08–15.02						
	PW2	399	6.83	4.01–17.99	4.22	2.36–11.04	1	4.42		5.30		
2013	PH	139	5.29	4.01–8.56	5.03	3.71–8.38						
	SL	42	4.84	4.01–7.43	4.92	3.98–7.79						
	IL1	275	5.80	4.00–15.14	6.80	4.45–17.72	1	4.24		5.22		1
	IL2	131	5.92	4.00–12.49	5.26	3.39–11.19						
	IL3	78	5.49	4.04–9.01	4.22	2.99–7.27						
	IL4	65	5.18	4.00–9.50	3.86	2.89–7.50						
	FL	3	4.20	4.00–4.36	3.18	3.01–3.31	1	4.29		5.30		1
	FW	22	5.20	4.07–7.97	5.35	4.04–8.32	1	4.53		5.62		
	LN	13	4.62	4.01–6.04	5.21	4.43–6.93						
	SNN	24	4.99	4.15–7.48	3.73	3.02–5.71	2	4.70	4.34–5.06	5.87	5.34–6.40	2
	AL1	239	5.95	4.01–8.80	6.74	4.27–11.48						
	AL2	150	4.45	4.00–7.18	5.19	4.52–8.49						
	SPL	15	4.59	4.09–5.68	3.92	3.42–4.91	1	5.53		7.09		
	SPW	363	7.32	4.01–21.42	4.54	2.37–13.42						
	SPN	11	4.94	4.23–6.50	2.74	2.30–3.68						
	GL1	47	4.79	4.00–5.94	5.12	4.14–6.74						
	GL2	33	4.57	4.05–6.54	5.08	4.37–8.11	1	4.14		5.06		
	GW1	540	9.05	4.00–31.45	5.96	2.40–19.25	2	4.34	4.17–4.50	5.42	4.06–5.77	1
	GW2	545	9.10	4.00–32.95	5.94	2.39–19.84	1	4.29		5.50		1
	GT1	166	5.40	4.01–10.48	3.32	2.38–6.58	1	4.01		4.88		1
	GT2	221	5.85	4.00–12.56	3.63	2.39–7.92	1	4.29		4.27		1
	LL1	40	4.90	4.01–7.19	4.19	3.35–6.31						
	LL2	11	4.61	4.15–5.41	4.18	3.69–4.94	1	4.04		4.91		1
	LW1	317	6.95	4.03–15.95	4.82	2.61–11.48	1	4.44		5.42		
	LW2	369	7.29	4.02–18.91	4.70	2.47–12.32	1	4.02		4.83		
	PL1	79	4.87	4.02–8.77	2.87	2.30–5.42						
	PL2	20	4.79	4.03–6.69	3.31	2.67–4.64						
	PW1	290	6.80	4.00–15.14	4.68	2.64–10.53						
	PW2	315	6.79	4.00–17.70	4.56	2.57–11.84						
Total		12444					28					18

^a^the total number of significant association SNPs detected by GLM and MLM models at the threshold of –log_10_(*p*) = 3.84,respectively.

^b^R2 value showing % the explained phenotypic variation.

^c^the number of significant SNPs detected by both the two models.

PH: plant height; SL: spike length; IL1: internode length1; IL2: internode length 2; IL3: internode length 3; IL4: internode length 4; FL: flag leaf length; FW: flag leaf width; LN: leaf numbers; SNN: stem node numbers; AL1: awn length1; AL2: awn length 2; SPL: spikelet length; SPW: spikelet width; SPN: spikelet numbers; GL1: glume length1; GL2: glume length 2; GW1: glume width 1; GW2: glume width 2; GT1: glume thickness 1; GT2: glume thickness 2; LL1: lemma length 1; LL2: lemma length 2; LW1: lemma width 1; LW2: lemma width 2; PL1: palea length 1; PL2: palea length 2; PW1: palea width 1; PW2: palea width 2.

**Table 4:** SNPs significantly associated with morphological traits and candidate genes/flanking genes.

Shared-Marker^a^	Chromosome		locus (cM)^d^	Trait	GLM LOG_10_P	MLM LOG_10_P	Flanking gene/ Gene Name
	D genome_Chr^b^	Chr_arm_IWGSC^c^					
GB5Y7FA01BCN94_233	2D	2DS	39.302	GW2-2012	14.25	4.02	Acc-1
GBQ4KXB02HJM7P_431	2D	2BL	55.616	GW1-2012	13.71	4.21	116F2
				GW1-2013	14.1	4.5	115G1
				GW2-2013	13.5	4.29	
				GW1-BLUP	15.55	5.22	
				GW2-BLUP	14.94	4.96	
GDEEGVY01EBF5V_261	2D	2DS	83.234	IL1-2013	7.38	4.24	Aux/IAA family protein gene
contig15239_471	2D	2DL	103.299	SPN-2012	5.03	4.07	LR34
							cytochrome P450
contig11810_520	3D	3B	57.498	SPN-2012	5.76	4.94	\
GCE8AKX01ALM0H_152	3D	3DL	95.542	SPN-2012	5.04	4.03	\
GBB4FNX02JQNSU_161	4D	4DS	33.619	SNN-2013	5.2	4.34	LR34
							cytochrome P450
F5MV3MU01BU5XD_286	4D	4DL	71.459	SPN-2012	4.76	4.18	glutathione-S-transferase 2
							glutathione-S-transferase 1
GDEEGVY02FSE3Z_84	4D	4DL	84.069	FL-2013	4.24	4.29	AXAH3
GBB4FNX01EWVYF_161	4D	4DL	104.181	SL-2012	6.09	4.12	BAM3
GDEEGVY02FLOCP_398	4D	4DL	132.198	GW1-2012	29.02	4.34	1J9.1
				GW1-BLUP	29.86	4.59	1J9.2
				GW2-BLUP	29.67	4.28	Rht-D1b
GBB4FNX01BXV4Q_52	5D	5DL	113.167	GT1-2013	8.62	4.01	CKX2.5
				GT2-2013	8.6	4.29	
GDRF1KQ02F8V30_278	5D	5DL	151.266	GW1-2012	34.97	5.03	Ig1
				GW1-BLUP	33.06	4.65	TSAlike
				GW2-BLUP	35.09	4.34	
GBUVHFX01CI5PL_126	6D	6BS	59.136	SNN-2013	5.62	5.06	ZCCT2
							ZCCT1
							SNF2P
GBF1XID02IP0NJ_181	7D	7AS	76.223	LL2-2013	4.67	4.04	Acc-2
							ERF1
**Shared-Marker**^a^	**Flanking gene/Gene Annonation**				**GenBank Accession**	**References**	
GB5Y7FA01BCN94_233	Triticum aestivum clone BAC 122F14 plastid acetyl-CoA carboxylase (Acc-1) gene, nuclear gene for plastid product				EU660902.1	Chaoupska *et al.* 2008	
GBQ4KXB02HJM7P_431	Triticum monococcum BAC clones 116F2 and 115G1 gene sequence				AF459639.1	SanMiguel *et al.* 2002	
	Triticum monococcum BAC clones 116F2 and 115G2 gene sequence				AF459639.1	SanMiguel *et al.* 2002	
GDEEGVY01EBF5V_261	Aegilops speltoides auxin-responsive Aux/IAA family protein gene, exons 3 through 6 and partial cds				FJ236275.1	Haudry *et al.* 2008	
contig15239_471	Triticum aestivum cultivar Chinese Spring hexose carrier, LR34, cytochrome P450, lectin receptor kinases, and cytochrome P450 genes				FJ436983.1	Wicker *et al.* 2009; Krattinger *et al.* 2009	
	Triticum aestivum cultivar Chinese Spring hexose carrier, LR34, cytochrome P450, lectin receptor kinases, and cytochrome P451 genes				FJ436983.1	Wicker *et al.* 2009; Krattinger *et al.* 2009	
contig11810_520	\				\	\	
GCE8AKX01ALM0H_152	\				\	\	
GBB4FNX02JQNSU_161	Triticum aestivum cultivar Chinese Spring hexose carrier, LR34, cytochrome P450, lectin receptor kinases, and cytochrome P450 genes				FJ436983.1	Wicker *et al.* 2009; Krattinger *et al.* 2009	
	Triticum aestivum cultivar Chinese Spring hexose carrier, LR34, cytochrome P450, lectin receptor kinases, and cytochrome P451 genes				FJ436983.1	Wicker *et al.* 2009; Krattinger *et al.* 2009	
F5MV3MU01BU5XD_286	Aegilops tauschii glutathione-S-transferase 2 and glutathione S-transferase 1 genes				AY013753.1	Xu *et al.* 2002	
	Aegilops tauschii glutathione-S-transferase 2 and glutathione S-transferase 1 genes				AY013753.1	Xu *et al.* 2002	
GDEEGVY02FSE3Z_84	Hordeum vulgare arabinoxylan arabinofuranohydrolase (AXAH3) gene				JQ303077.1	Laidlaw *et al.* 2012	
GBB4FNX01EWVYF_161	Hordeum vulgare subsp. vulgare mRNA for beta-amylase (BAM3 gene)				FN179395.1	Radchuk *et al.* 2009	
GDEEGVY02FLOCP_398	Triticum aestivum clone BAC 1J9 Tmemb_185A domain-containing protein (1J9.1), EamA domain-containing protein (1J9.2), and Rht-D1b (Rht-D1b) genes				HQ435325.1	Duan *et al.* 2012	
	Triticum aestivum clone BAC 1J9 Tmemb_185A domain-containing protein (1J9.1), EamA domain-containing protein (1J9.2), and Rht-D1b (Rht-D1b) genes				HQ435325.1	Duan *et al.* 2012	
	Triticum aestivum clone BAC 1J9 Tmemb_185A domain-containing protein (1J9.1), EamA domain-containing protein (1J9.2), and Rht-D1b (Rht-D1b) genes				HQ435325.1	Duan *et al.* 2012	
**Shared-Marker**^a^	**Flanking gene/Gene Annonation**				**GenBank Accession**	**References**	
GBB4FNX01BXV4Q_52	Triticum aestivum clone BAC 400M29 cytokinin oxidase/dehydrogenase gene				JN381556.1	Mameauxs *et al.* 2012	
GDRF1KQ02F8V30_278	Zea mays indole-3-glycerol phosphate lyase (Igl) gene,and putative tryptophan synthase alpha (TSAlike) gene				AF271383.1	Frey *et al.* 2000	
	Zea mays indole-3-glycerol phosphate lyase (Igl) gene,and putative tryptophan synthase alpha (TSAlike) gene				AF271383.1	Frey *et al.* 2000	
GBUVHFX01CI5PL_126	Triticum monococcum phosphatidylserine decarboxylase, ZCCT2, ZCCT1, and SNF2P genes				AY485644.1	Yan *et al.* 2004	
	Triticum monococcum phosphatidylserine decarboxylase, ZCCT2, ZCCT1, and SNF2P genes				AY485644.1	Yan *et al.* 2004	
	Triticum monococcum phosphatidylserine decarboxylase, ZCCT2, ZCCT1, and SNF2P genes				AY485644.1	Yan *et al.* 2004	
GBF1XID02IP0NJ_181	Triticum aestivum clone BAC 1354M21 cytosolic acetyl-CoA carboxylase (Acc-2) gene				EU660892.1	Chaoupska *et al.* 2008	
	Triticum turgidum subsp. durum ethylene response factor 1 (ERF1) gene				KJ689812.1	Makhloufi *et al.* 2014	

^a^The shared-markers were significant in both GLM and MLM model at the threshhold of −log_10_(*p*) = 3.84.

^b^Chromosomal location information in diploid ancestors Aegilops tauschii.

^c^Chromosomal location information in polyploid wheat that display by the best hit on IWGSC.

^d^Genetic distance in diploid Aegilops tauschii.
