# Supplementary material for: Genome-wide association study of 29 morphological traits in Aegilops tauschii
Source: Sci Rep. 2015 Oct 27;5:15562. doi: 10.1038/srep15562 (PMC4622089; doi:10.1038/srep15562)
Supplement: Supplementary Information [file srep15562-s7.pdf]

Genome-wide association studies of 29 morphological traits in *Aegilops tauschii*

Yaxi Liu[1, 3*], Lang Wang[1, 3], Shuangshuang Mao[1], Kun Liu[1], Yanli Lu[2], Jirui Wang[1], Yuming Wei[1] and Youliang Zheng[1*]

[1] Triticeae Research Institute, Sichuan Agricultural University, Wenjiang, Chengdu 611130, China

[2] Maize Research Institute, Sichuan Agricultural University, Wenjiang, Chengdu 611130, China

[3] Yaxi Liu and Lang Wang contributed equally to this work.

[*]Corresponding authors: Yaxi Liu, Phone: +86-28-86290951, Fax: +86-28-82650350, e-mail: liuyaxi@sicau.edu.cn ; Youliang Zheng, Phone: +86-28-86290951, Fax: +86-28-82650350, e-mail: ylzheng@sicau.edu.cn

Supplementary Fig. S1 1 to 29 Phenotypic distribution and genome-wide association scan for all investigated morphological traits. (a) A histogram showing the distribution of each phenotype of year 2012 and 2013 in the *Aegilops tauschii* diversity panel. (b) Boxplot showing the mean, median and range of phenotypic variation of each *Aegilops tauschii* subpopulation independently. (c) Quantile-Quantile (Q-Q) plot showing the expected null distribution of *p* values, assuming no associations, represented as a solid black line; distribution of *p* values observed using the general linear model (GLM) represented as a dark goldenrod plot; distribution of *p* values observed using mixed linear model (MLM) represented as a dark green plot. (d) A Manhattan plot for the seven chromosomes carrying the significant markers detected by GLM and MLM models in year 2012 and 2013, *p* value is converted into $-\log_{10}(p)$, threshold of 3.84 are indicated by horizontal dashed lines.

1

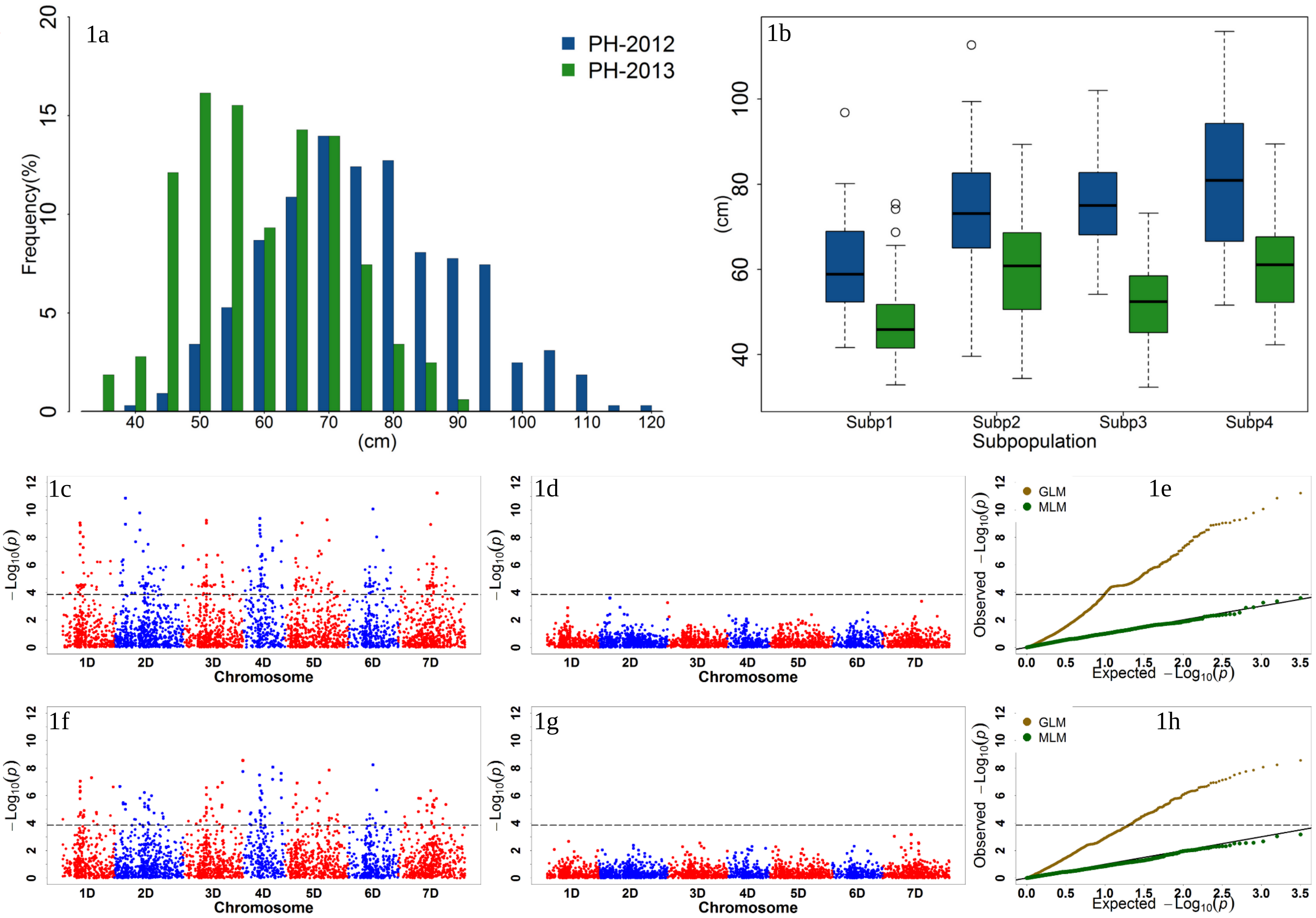
1a
PH-2012
PH-2013
Frequency(%)
(cm)
1b
(cm)
Subp1
Subp2
Subp3
Subp4
Subpopulation
1c
1d
1e
1f
1g
1h
Chromosome
1D
2D
3D
4D
5D
6D
7D
GLM
MLM
Expected −Log10(p)
Observed −Log10(p)

2

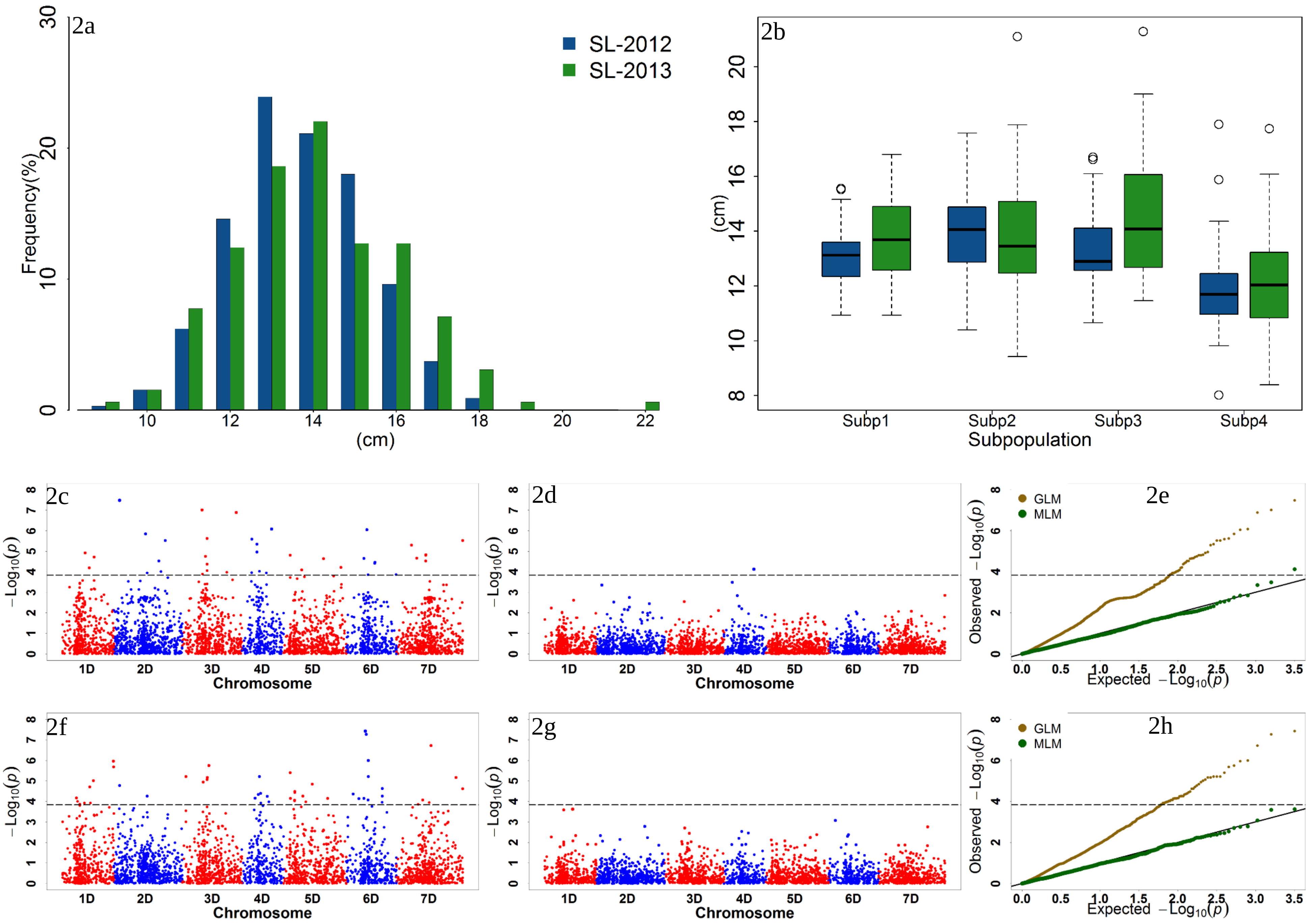
2a
SL-2012
SL-2013
Frequency(%)
(cm)
2b
(cm)
Subp1
Subp2
Subp3
Subp4
Subpopulation
2c
2d
2e
2f
2g
2h
Chromosome
1D
2D
3D
4D
5D
6D
7D
GLM
MLM
Observed −Log10(p)
Expected −Log10(p)

3

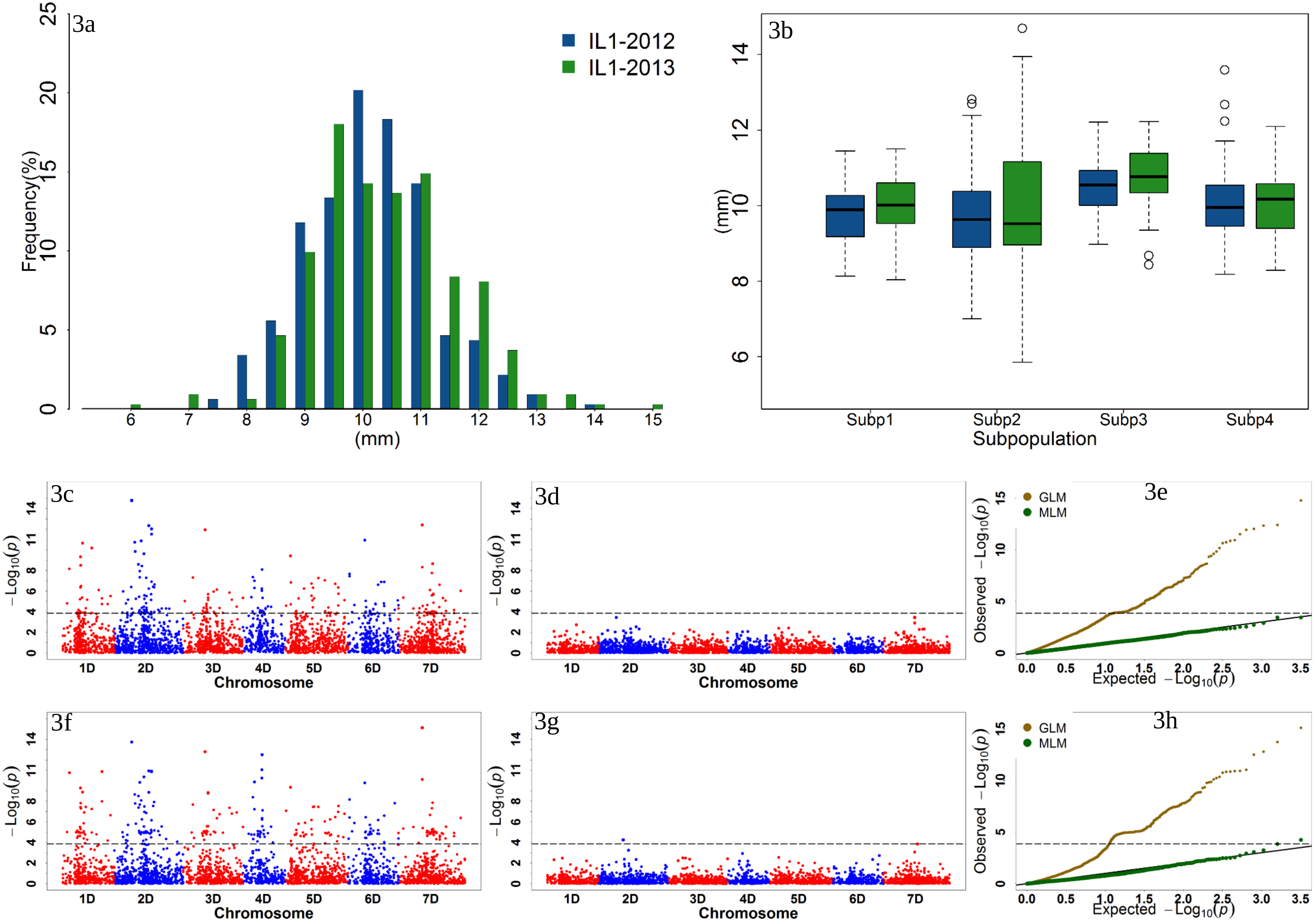

3a
IL1-2012
IL1-2013
Frequency(%)
0
5
10
15
20
25
6
7
8
9
10
11
12
13
14
15
(mm)
3b
(mm)
6
8
10
12
14
Subp1
Subp2
Subp3
Subp4
Subpopulation
3c
3d
3e
3f
3g
3h
−Log10(p)
0
2
4
6
8
11
14
1D
2D
3D
4D
5D
6D
7D
Chromosome
GLM
MLM
Observed −Log10(p)
Expected −Log10(p)
0.0
0.5
1.0
1.5
2.0
2.5
3.0
3.5

4

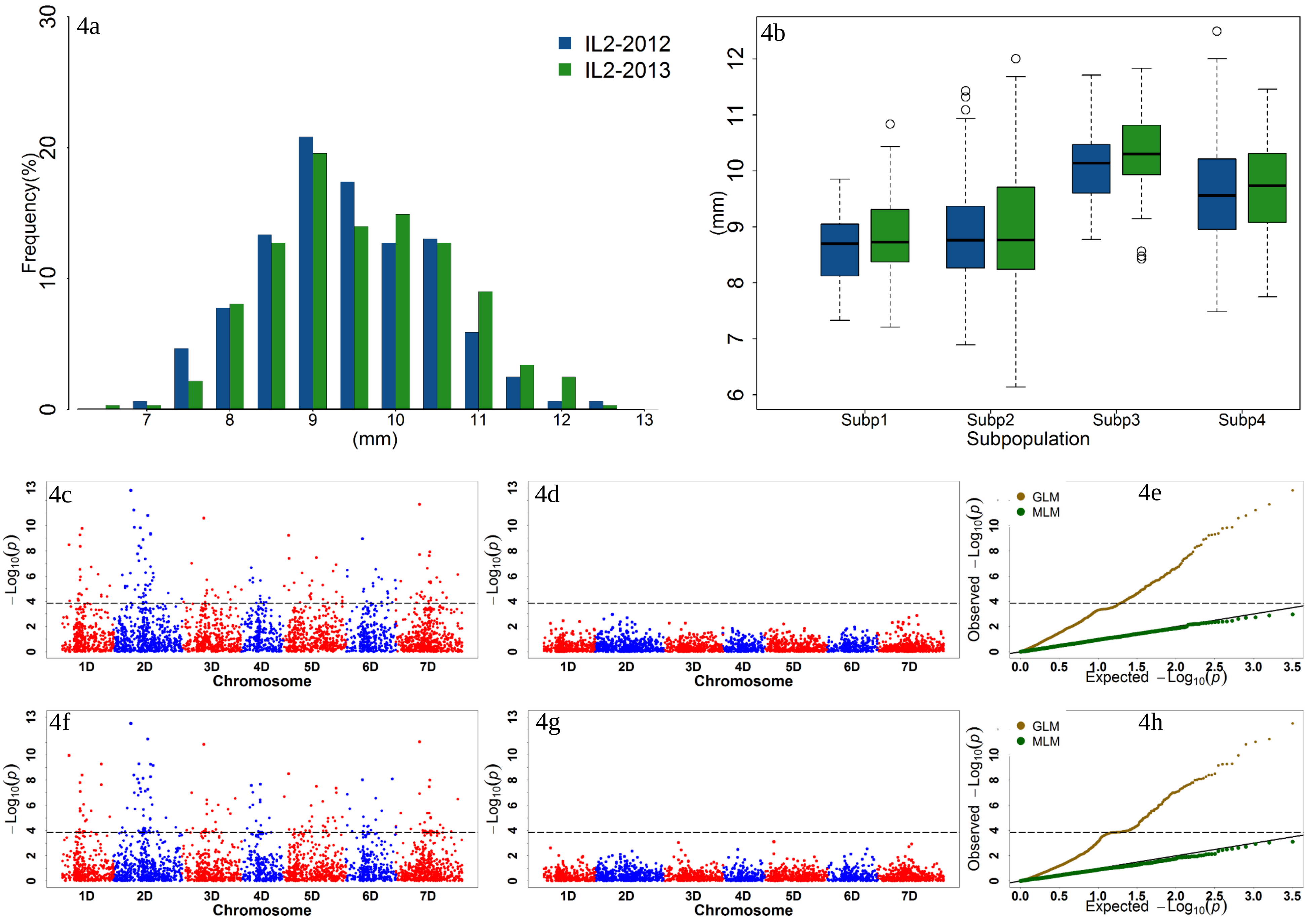
4a
IL2-2012
IL2-2013
Frequency(%)
(mm)
4b
(mm)
Subp1
Subp2
Subp3
Subp4
Subpopulation
4c
4d
4e
4f
4g
4h
GLM
MLM
Chromosome
1D
2D
3D
4D
5D
6D
7D
Observed
Expected

5

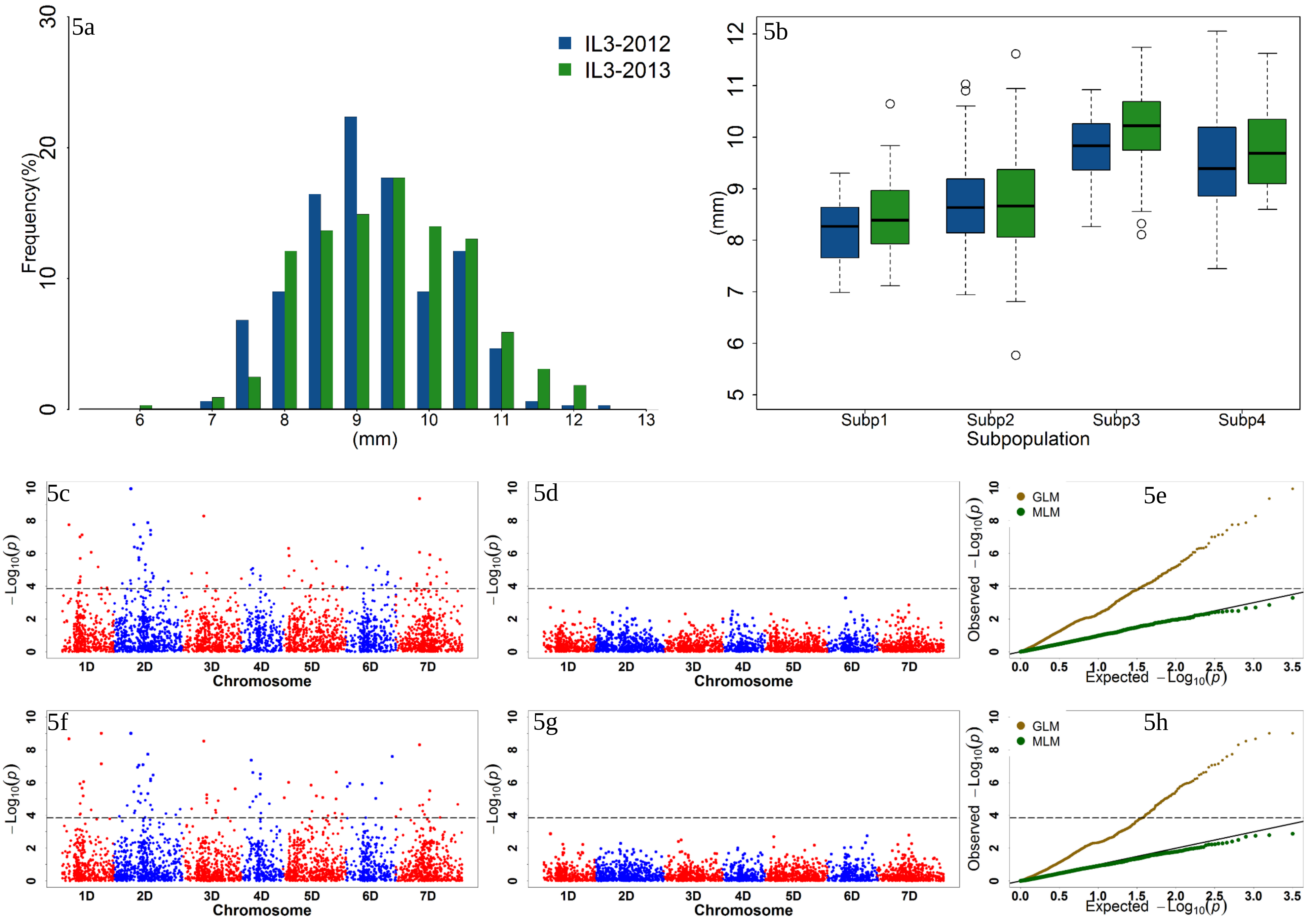

5a
IL3-2012
IL3-2013
Frequency(%)
(mm)
5b
(mm)
Subp1
Subp2
Subp3
Subp4
Subpopulation
5c
5d
5e
5f
5g
5h
1D
2D
3D
4D
5D
6D
7D
Chromosome
GLM
MLM
Observed $-\mathrm{Log}_{10}(p)$
Expected $-\mathrm{Log}_{10}(p)$

6

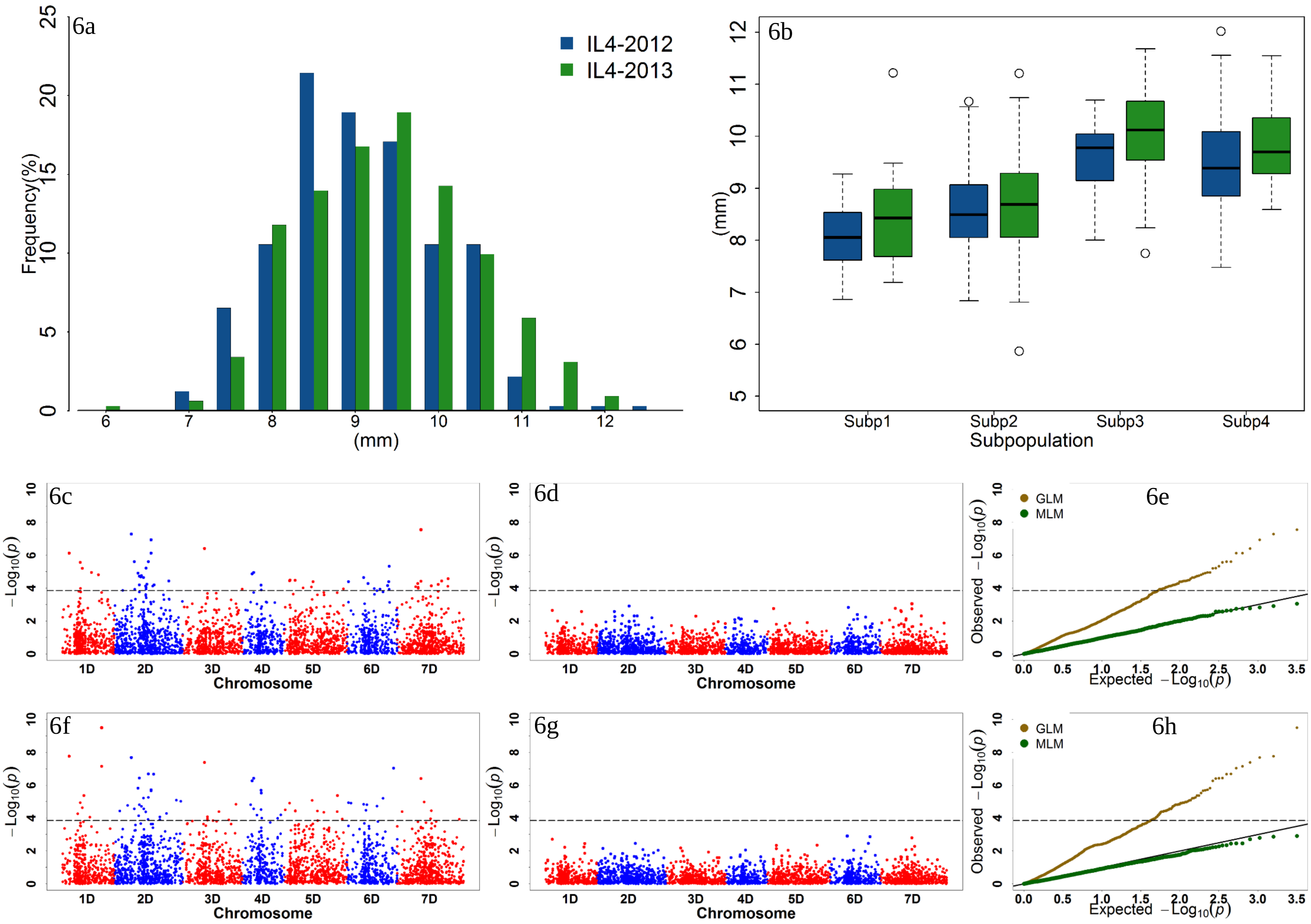

6a
IL4-2012
IL4-2013
Frequency(%)
(mm)
6b
(mm)
Subp1
Subp2
Subp3
Subp4
Subpopulation
6c
6d
6e
6f
6g
6h
GLM
MLM
Chromosome
1D
2D
3D
4D
5D
6D
7D
Observed
Expected

7

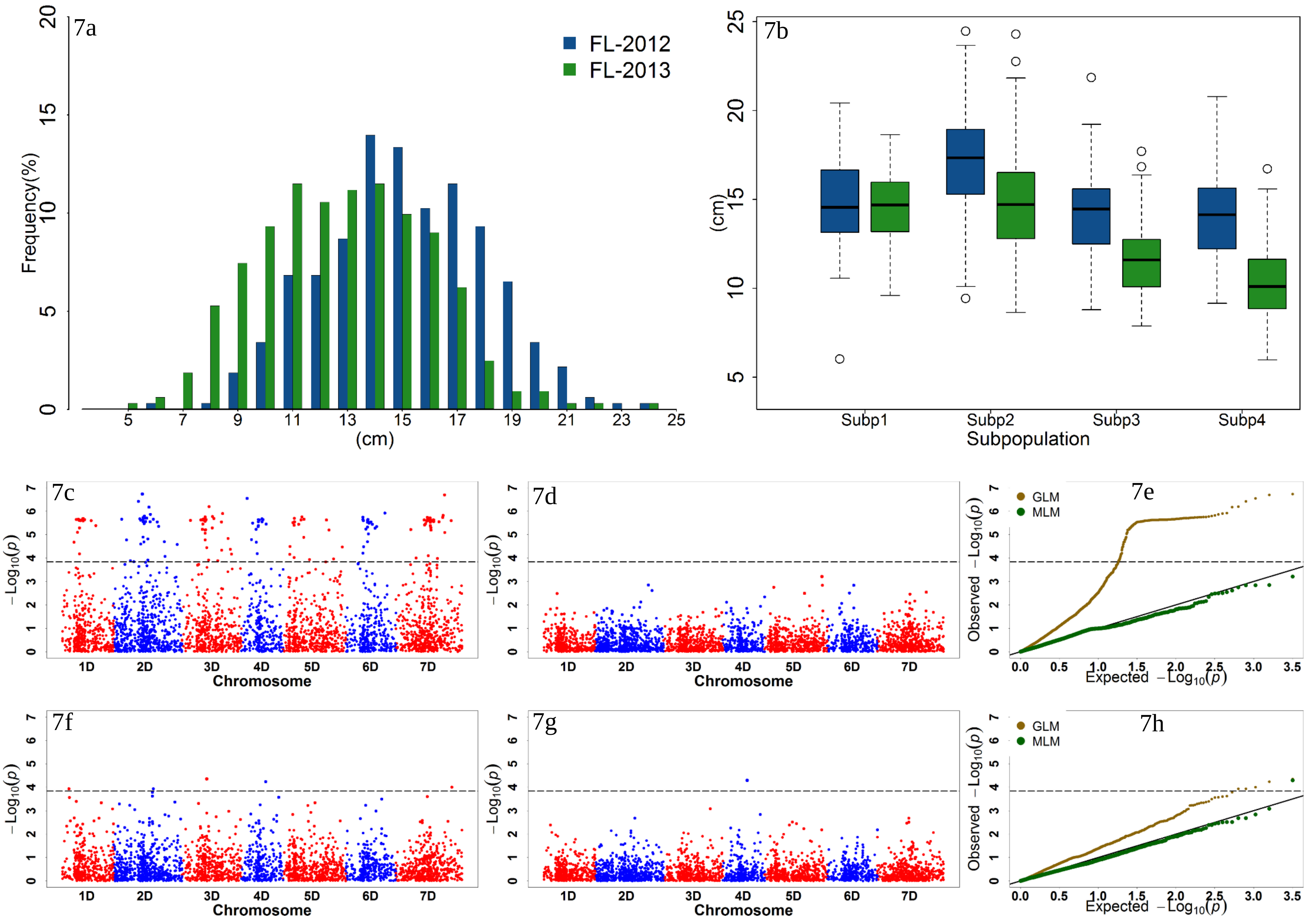
7a
FL-2012
FL-2013
Frequency(%)
(cm)
7b
(cm)
Subp1
Subp2
Subp3
Subp4
Subpopulation
7c
7d
7e
7f
7g
7h
GLM
MLM
1D
2D
3D
4D
5D
6D
7D
Chromosome
$-\mathrm{Log}_{10}(p)$
Observed $-\mathrm{Log}_{10}(p)$
Expected $-\mathrm{Log}_{10}(p)$

8

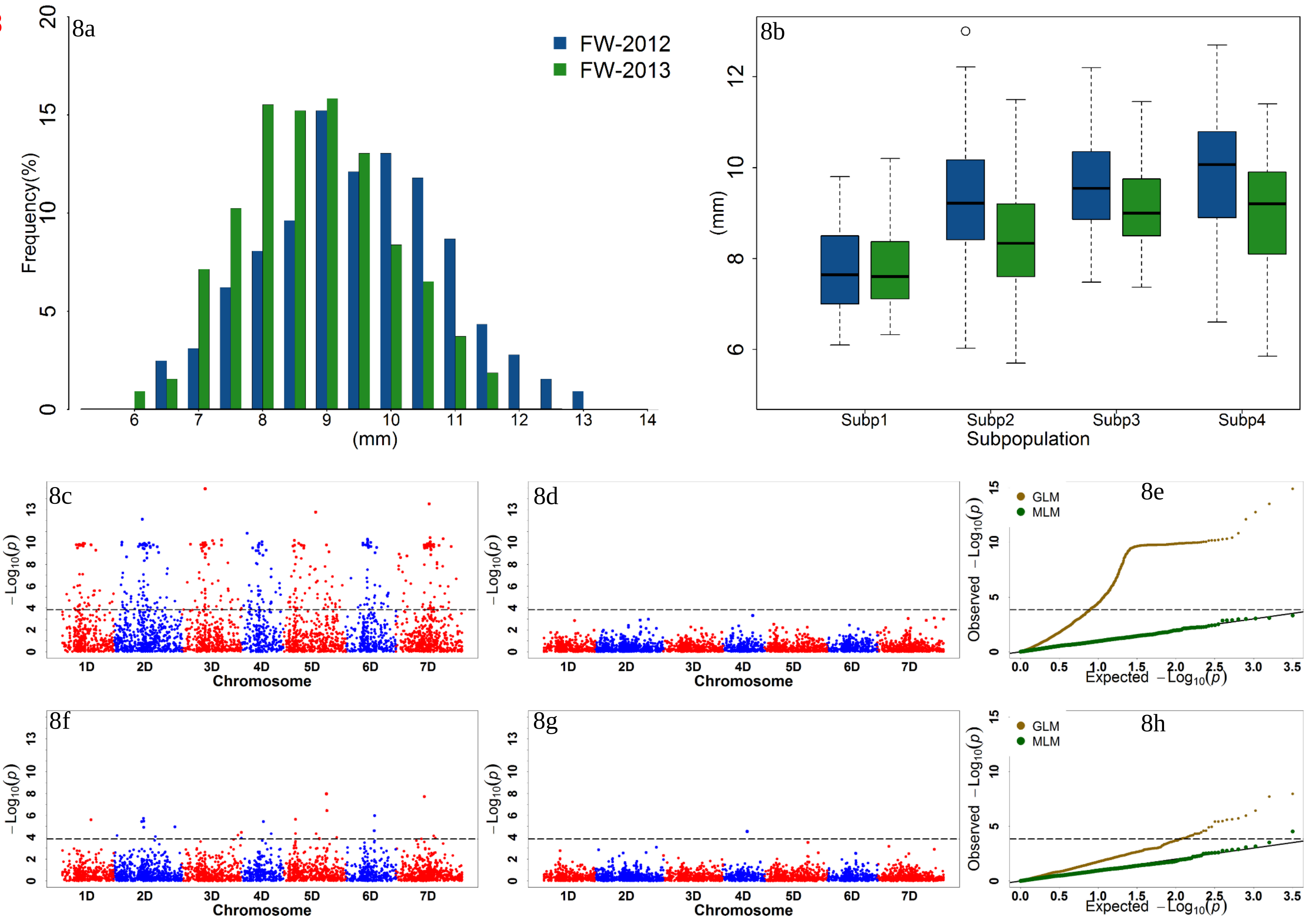
8a
FW-2012
FW-2013
Frequency(%)
(mm)
8b
(mm)
Subp1
Subp2
Subp3
Subp4
Subpopulation
8c
8d
8e
8f
8g
8h
1D
2D
3D
4D
5D
6D
7D
Chromosome
GLM
MLM
Observed −Log10(p)
Expected −Log10(p)

9

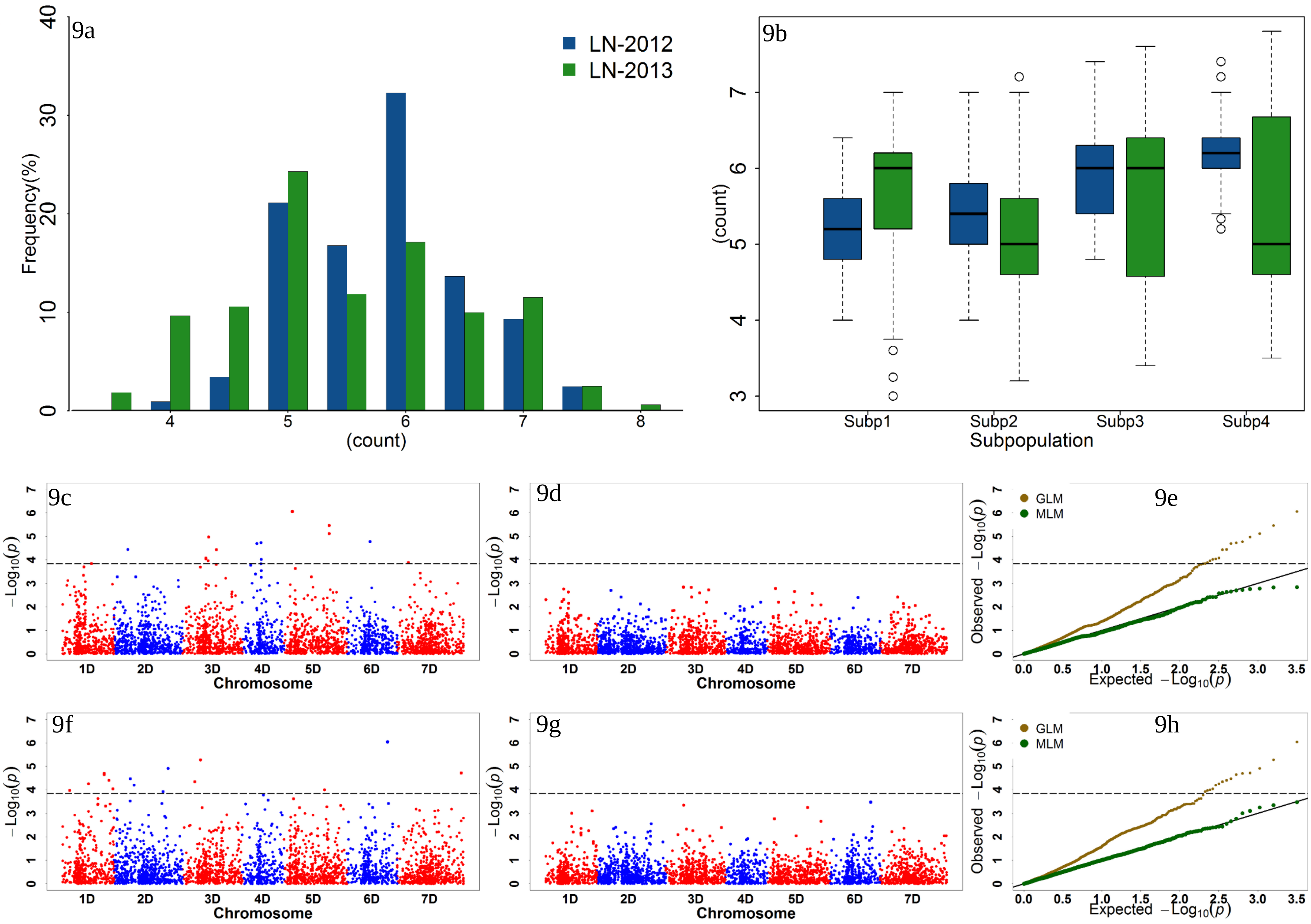
9a
LN-2012
LN-2013
Frequency(%)
(count)
9b
(count)
Subp1
Subp2
Subp3
Subp4
Subpopulation
9c
Chromosome
9d
Chromosome
9e
GLM
MLM
Observed −Log10(p)
Expected −Log10(p)
9f
Chromosome
9g
Chromosome
9h
GLM
MLM
Observed −Log10(p)
Expected −Log10(p)

10

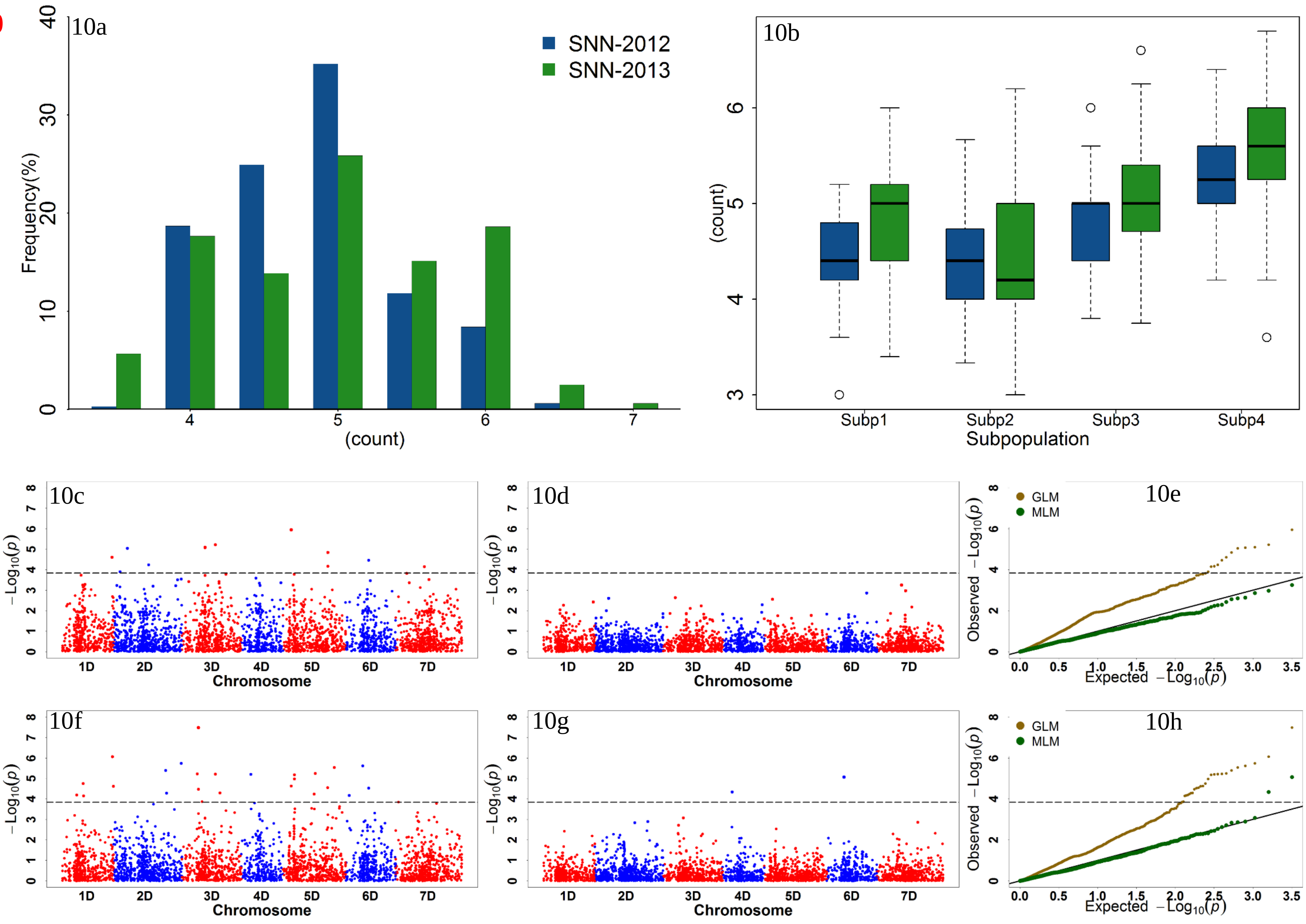
10a
SNN-2012
SNN-2013
Frequency(%)
(count)
10b
(count)
Subp1
Subp2
Subp3
Subp4
Subpopulation
10c
10d
10e
10f
10g
10h
GLM
MLM
−Log10(p)
Chromosome
1D
2D
3D
4D
5D
6D
7D
Observed −Log10(p)
Expected −Log10(p)

11

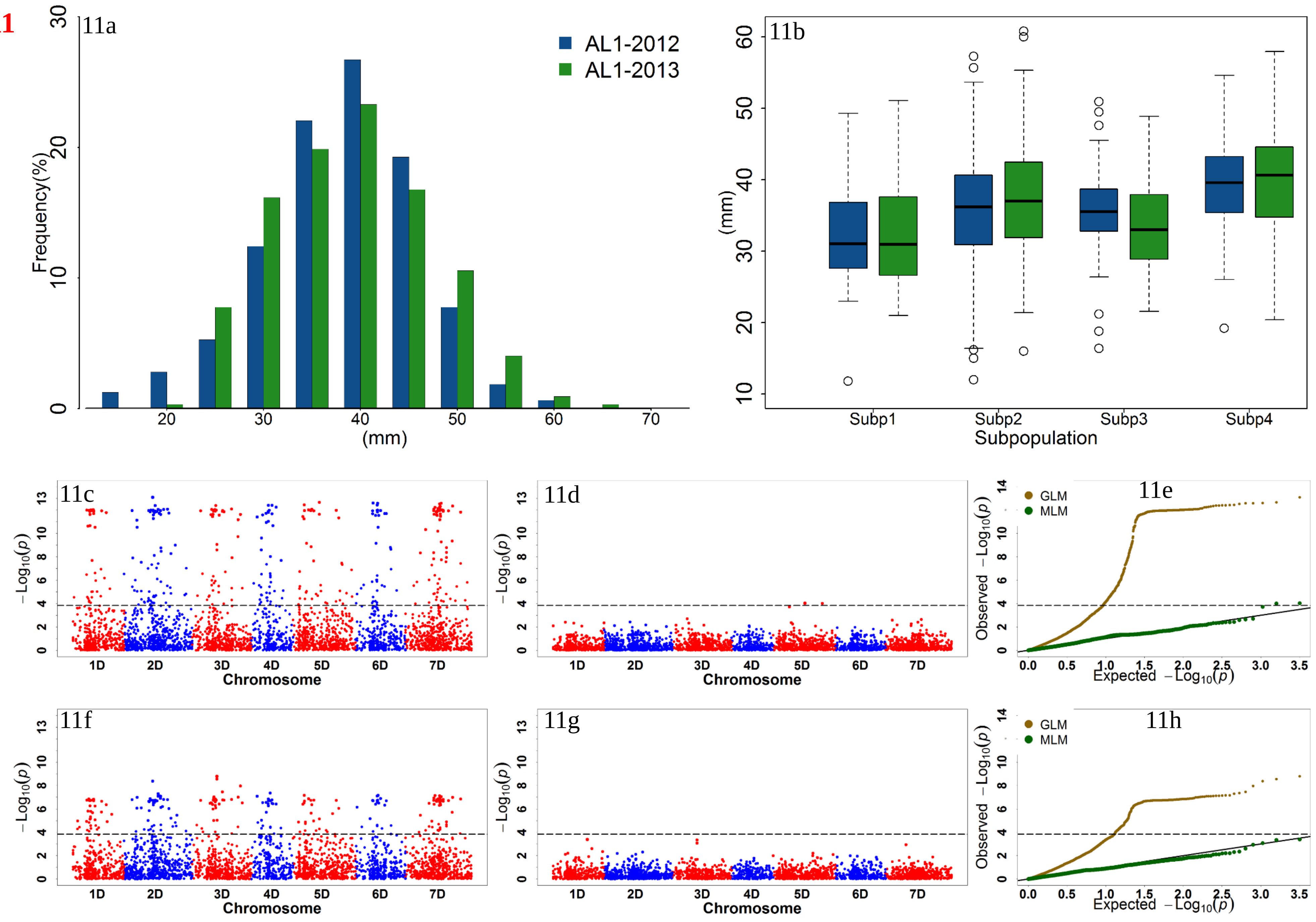
11a
AL1-2012
AL1-2013
Frequency(%)
(mm)
11b
(mm)
Subp1
Subp2
Subp3
Subp4
Subpopulation
11c
11d
11e
11f
11g
11h
GLM
MLM
Chromosome
1D
2D
3D
4D
5D
6D
7D
$-\mathrm{Log}_{10}(p)$
Observed $-\mathrm{Log}_{10}(p)$
Expected $-\mathrm{Log}_{10}(p)$

12

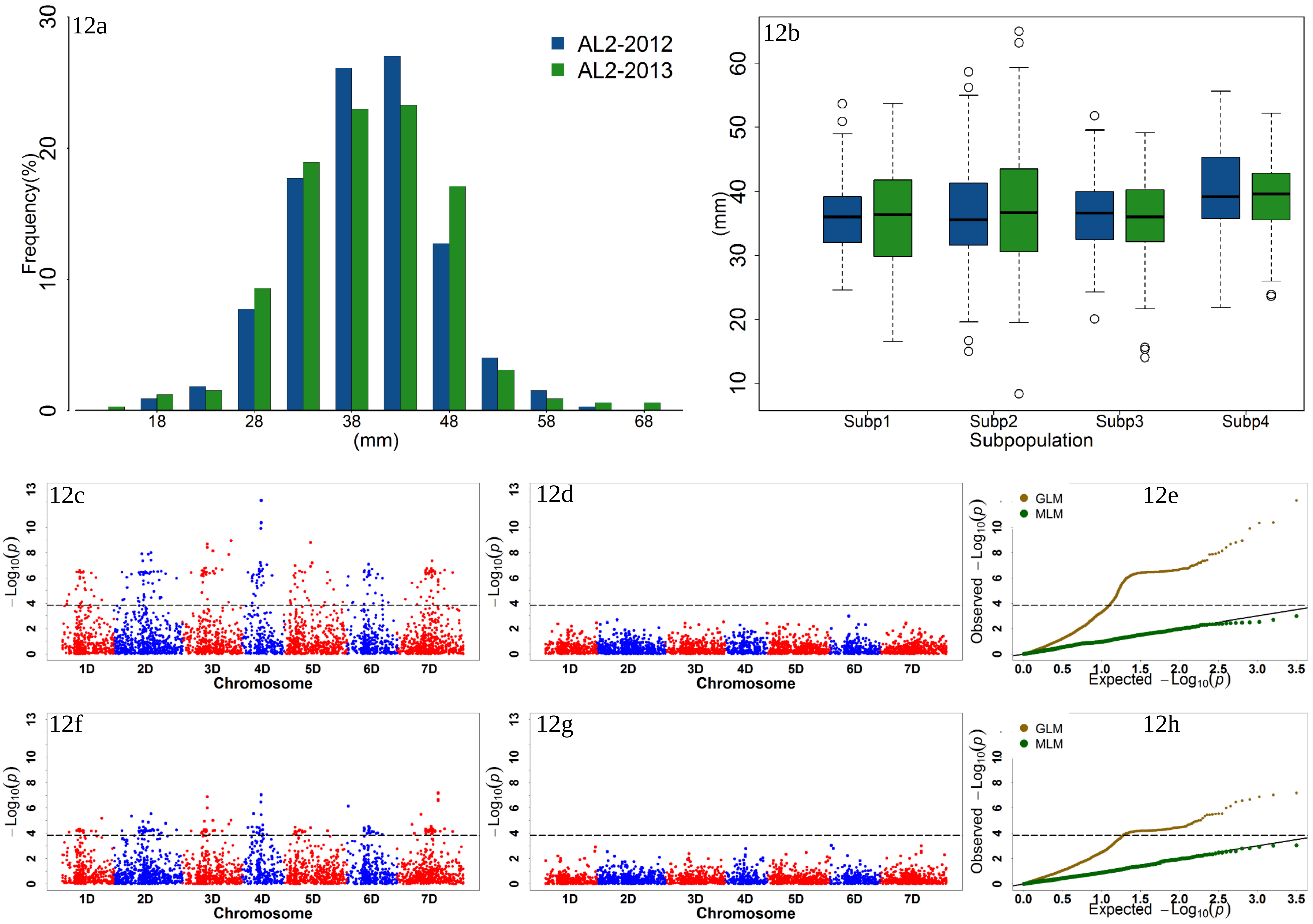

12a
AL2-2012
AL2-2013
Frequency(%)
0
10
20
30
18
28
38
48
58
68
(mm)
12b
(mm)
10
20
30
40
50
60
Subp1
Subp2
Subp3
Subp4
Subpopulation
12c
12d
12e
12f
12g
12h
GLM
MLM
1D
2D
3D
4D
5D
6D
7D
Chromosome
Observed −Log10(p)
Expected −Log10(p)

13

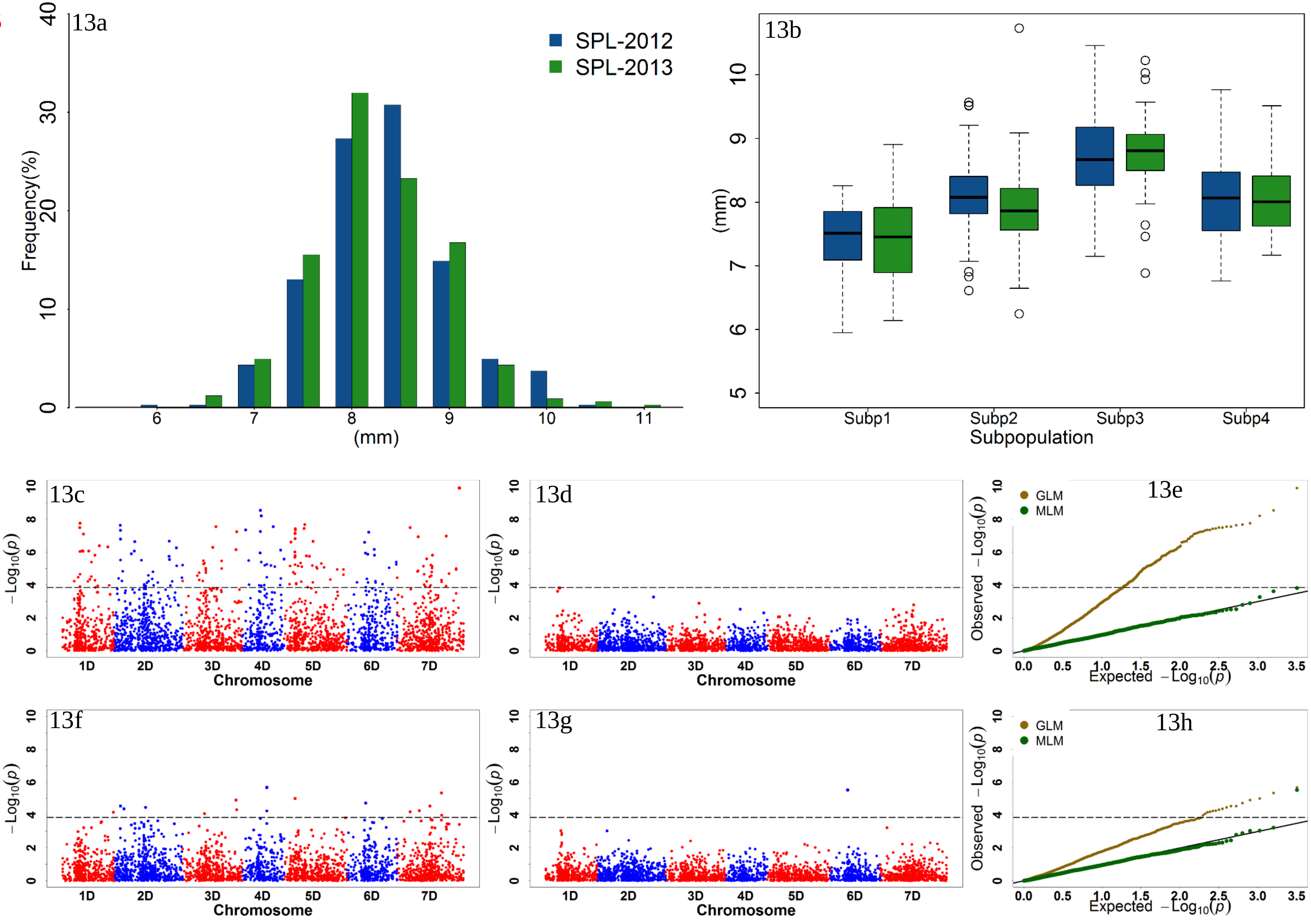
13a
SPL-2012
SPL-2013
Frequency(%)
0
10
20
30
40
6
7
8
9
10
11
(mm)
13b
(mm)
5
6
7
8
9
10
Subp1
Subp2
Subp3
Subp4
Subpopulation
13c
13d
13e
13f
13g
13h
−Log10(p)
Chromosome
1D
2D
3D
4D
5D
6D
7D
GLM
MLM
Observed −Log10(p)
Expected −Log10(p)
0.0
0.5
1.0
1.5
2.0
2.5
3.0
3.5

14

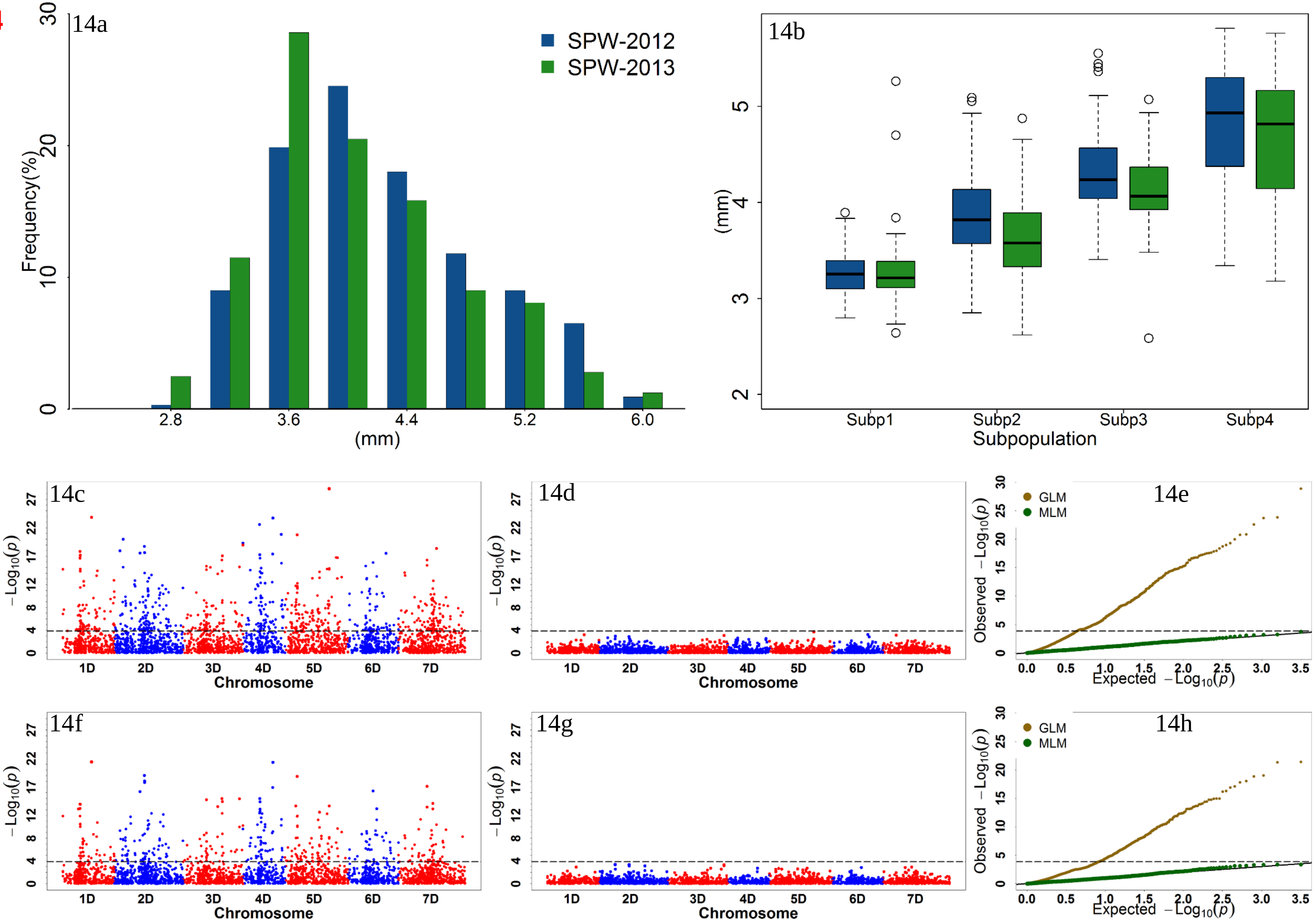
14a
SPW-2012
SPW-2013
Frequency(%)
(mm)
14b
(mm)
Subp1
Subp2
Subp3
Subp4
Subpopulation
14c
14d
14e
GLM
MLM
14f
14g
14h
1D
2D
3D
4D
5D
6D
7D
Chromosome
$-\log_{10}(p)$
Observed $-\log_{10}(p)$
Expected $-\log_{10}(p)$

15

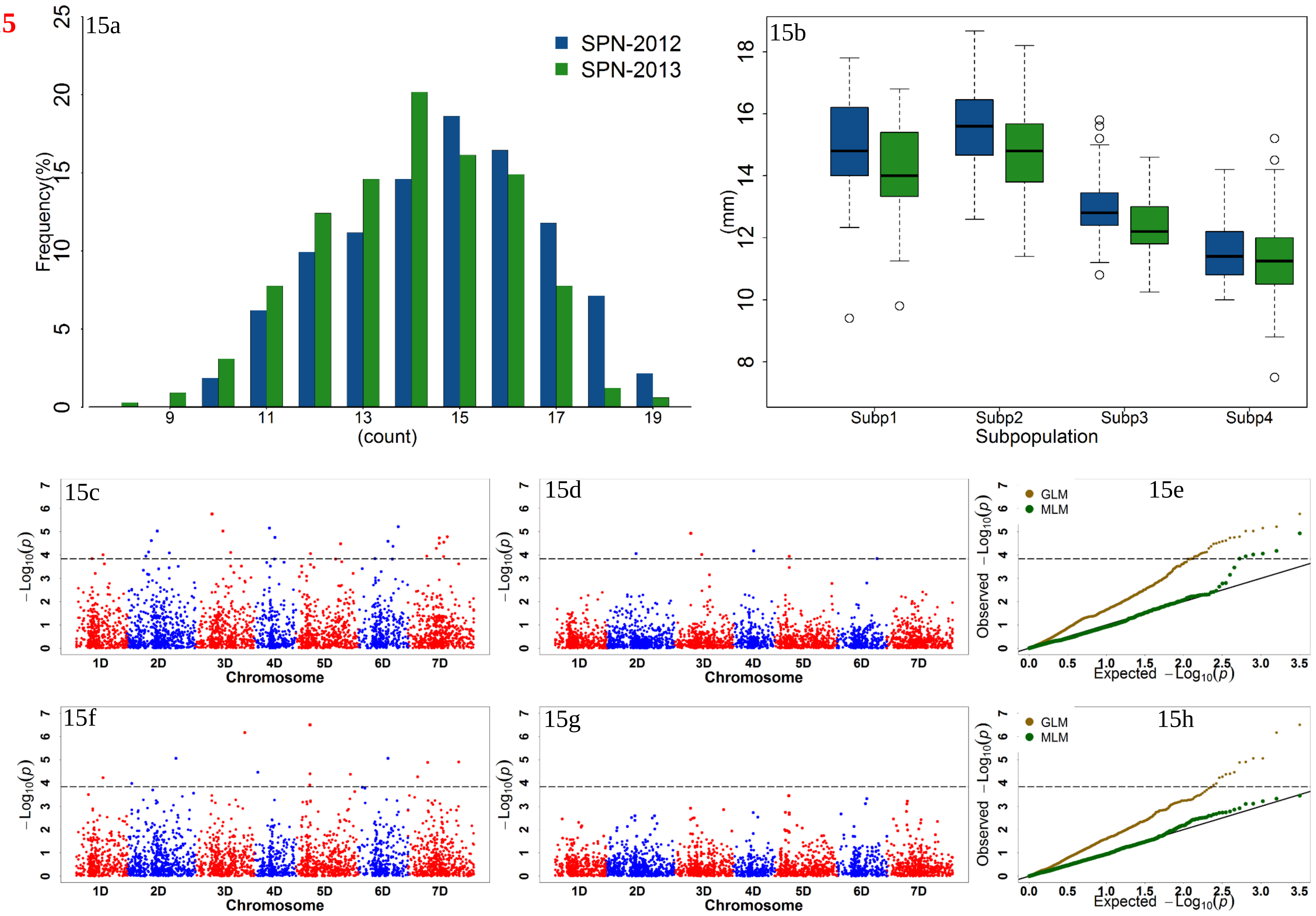
15a
SPN-2012
SPN-2013
Frequency(%)
(count)
15b
(mm)
Subp1
Subp2
Subp3
Subp4
Subpopulation
15c
15d
15e
15f
15g
15h
Chromosome
1D
2D
3D
4D
5D
6D
7D
GLM
MLM
Observed −Log10(p)
Expected −Log10(p)

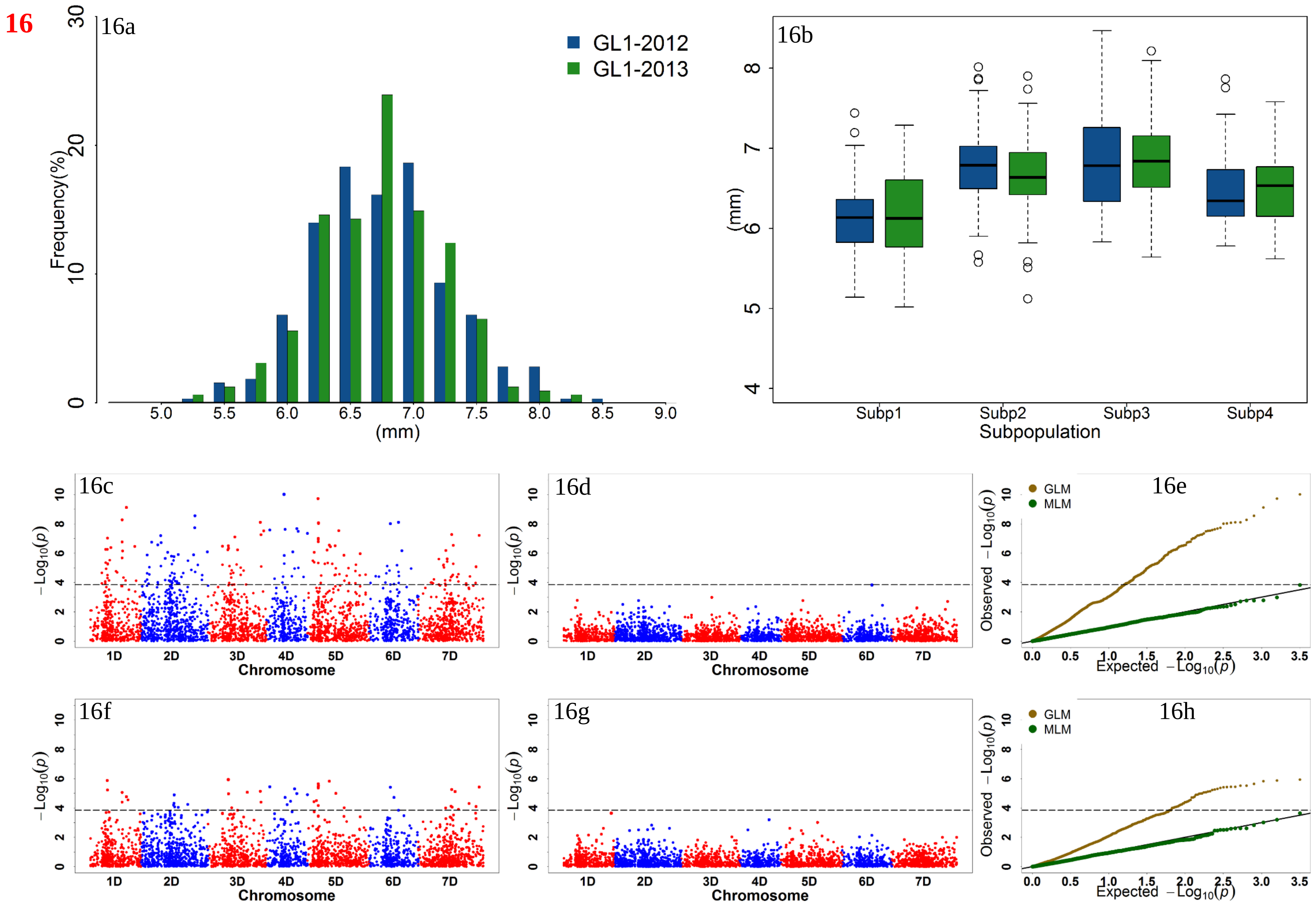
16
16a
GL1-2012
GL1-2013
Frequency(%)
(mm)
16b
(mm)
Subp1
Subp2
Subp3
Subp4
Subpopulation
16c
16d
16e
16f
16g
16h
Chromosome
1D
2D
3D
4D
5D
6D
7D
GLM
MLM
Observed −Log10(p)
Expected −Log10(p)

17

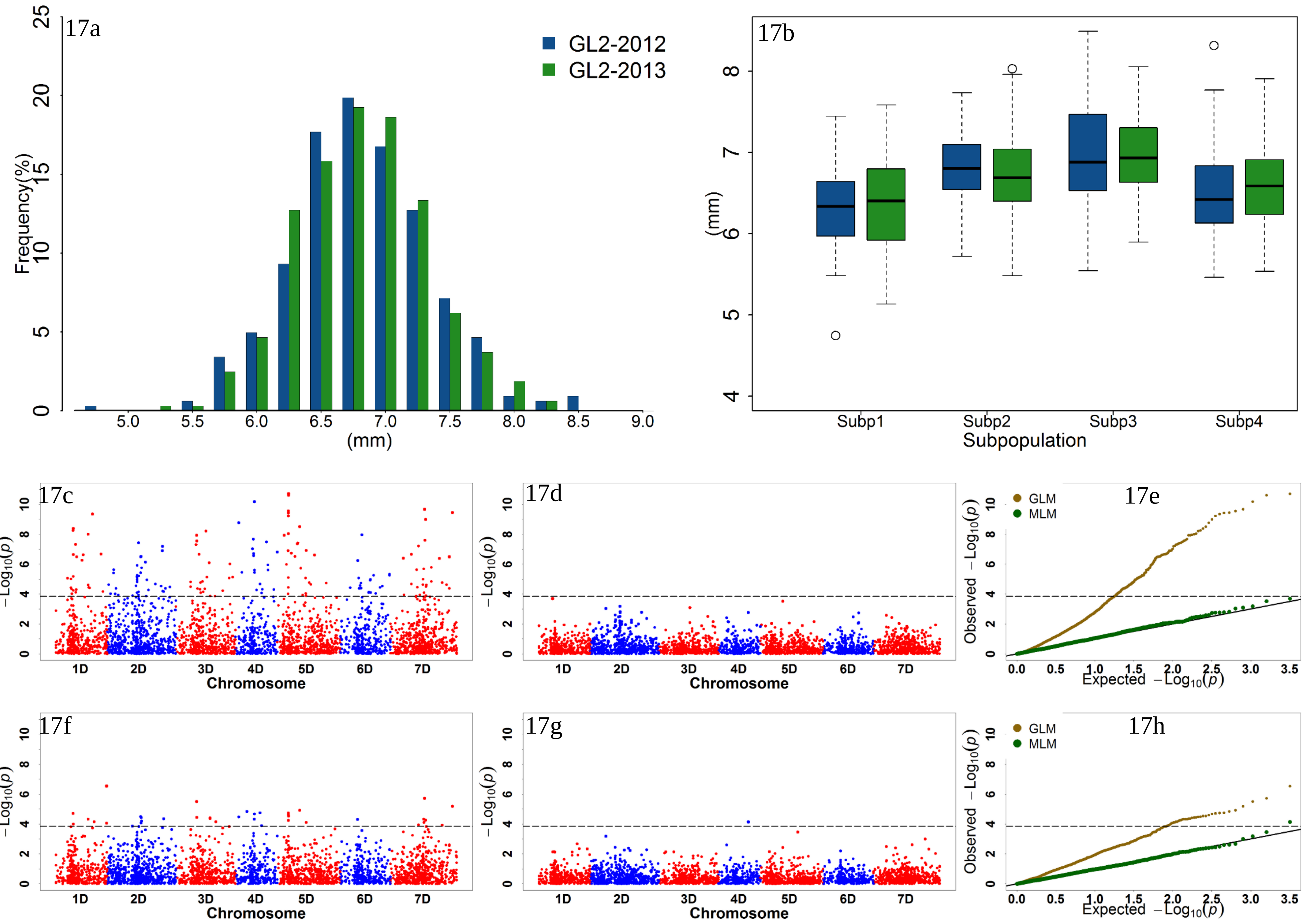
17a
GL2-2012
GL2-2013
Frequency(%)
0
5
10
15
20
25
5.0
5.5
6.0
6.5
7.0
7.5
8.0
8.5
9.0
(mm)
17b
(mm)
4
5
6
7
8
Subp1
Subp2
Subp3
Subp4
Subpopulation
17c
17d
17e
GLM
MLM
17f
17g
17h
1D
2D
3D
4D
5D
6D
7D
Chromosome
$-\mathrm{Log}_{10}(p)$
Observed $-\mathrm{Log}_{10}(p)$
Expected $-\mathrm{Log}_{10}(p)$
0
2
4
6
8
10
0.0
0.5
1.0
1.5
2.0
2.5
3.0
3.5

18

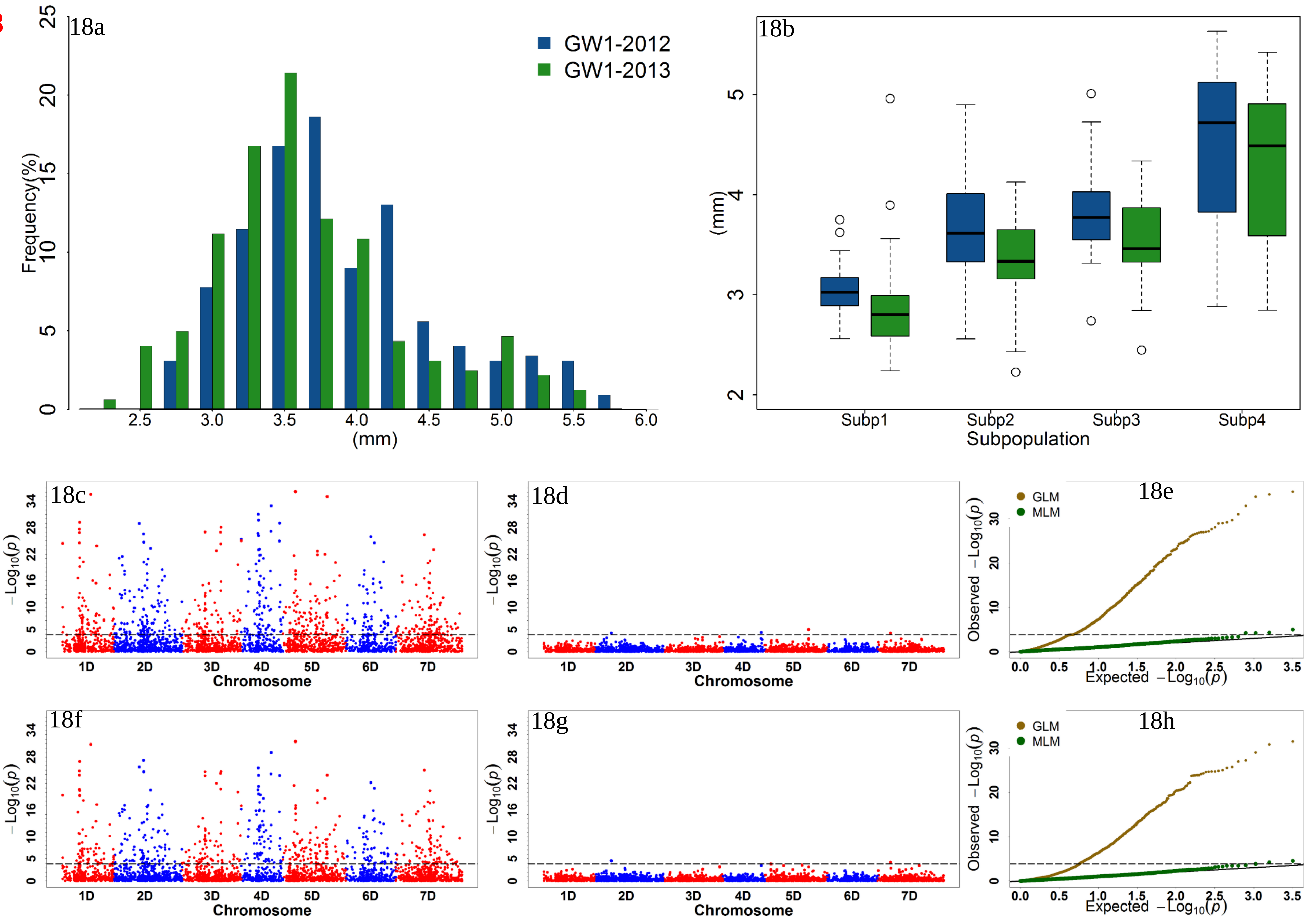
18a
GW1-2012
GW1-2013
Frequency(%)
(mm)
18b
(mm)
Subp1
Subp2
Subp3
Subp4
Subpopulation
18c
18d
18e
GLM
MLM
Chromosome
Observed −Log10(p)
Expected −Log10(p)
18f
18g
18h

19

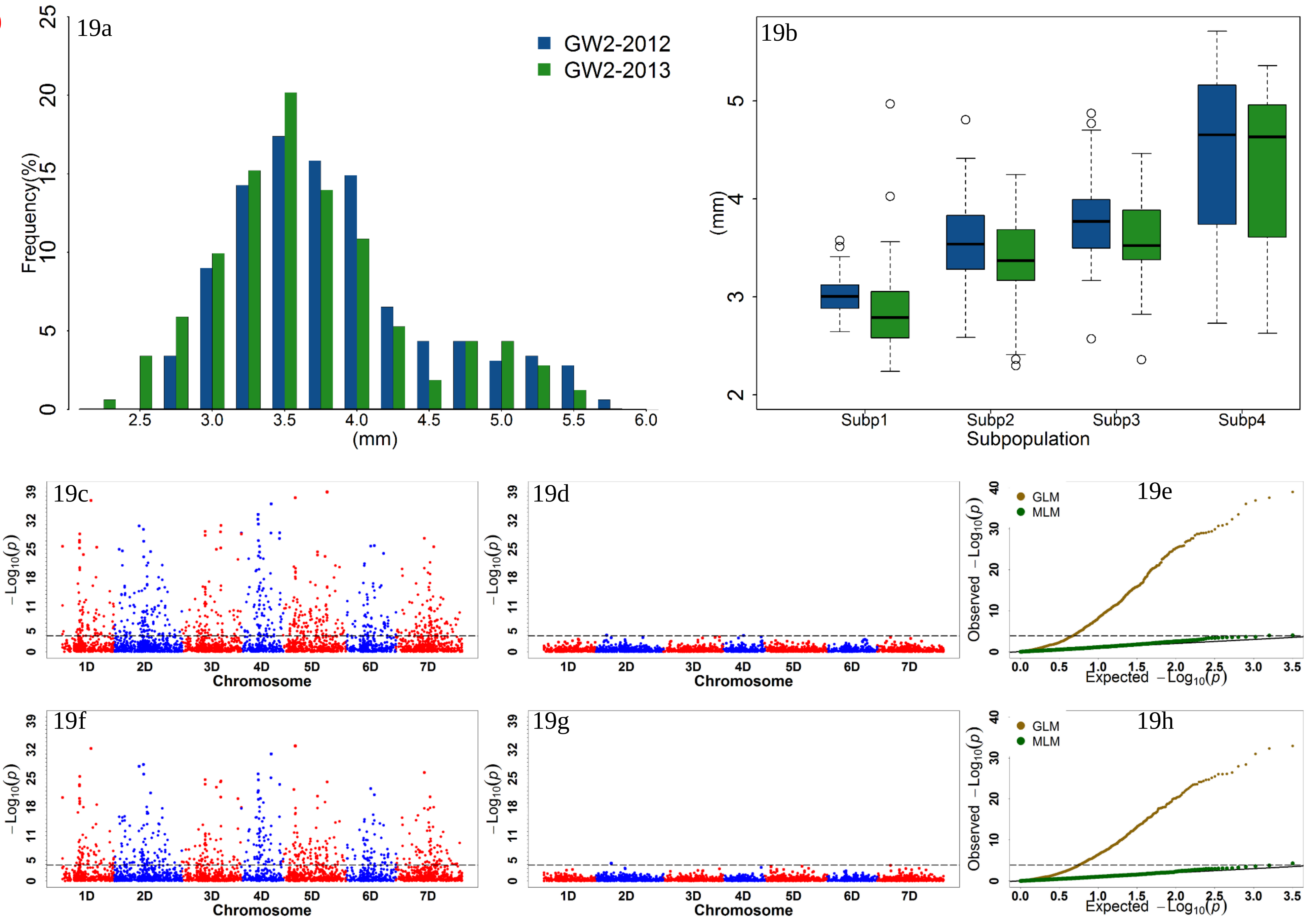

19a
GW2-2012
GW2-2013
Frequency(%)
(mm)
19b
(mm)
Subp1
Subp2
Subp3
Subp4
Subpopulation
19c
19d
19e
19f
19g
19h
−Log10(p)
Chromosome
1D
2D
3D
4D
5D
6D
7D
GLM
MLM
Observed −Log10(p)
Expected −Log10(p)

20

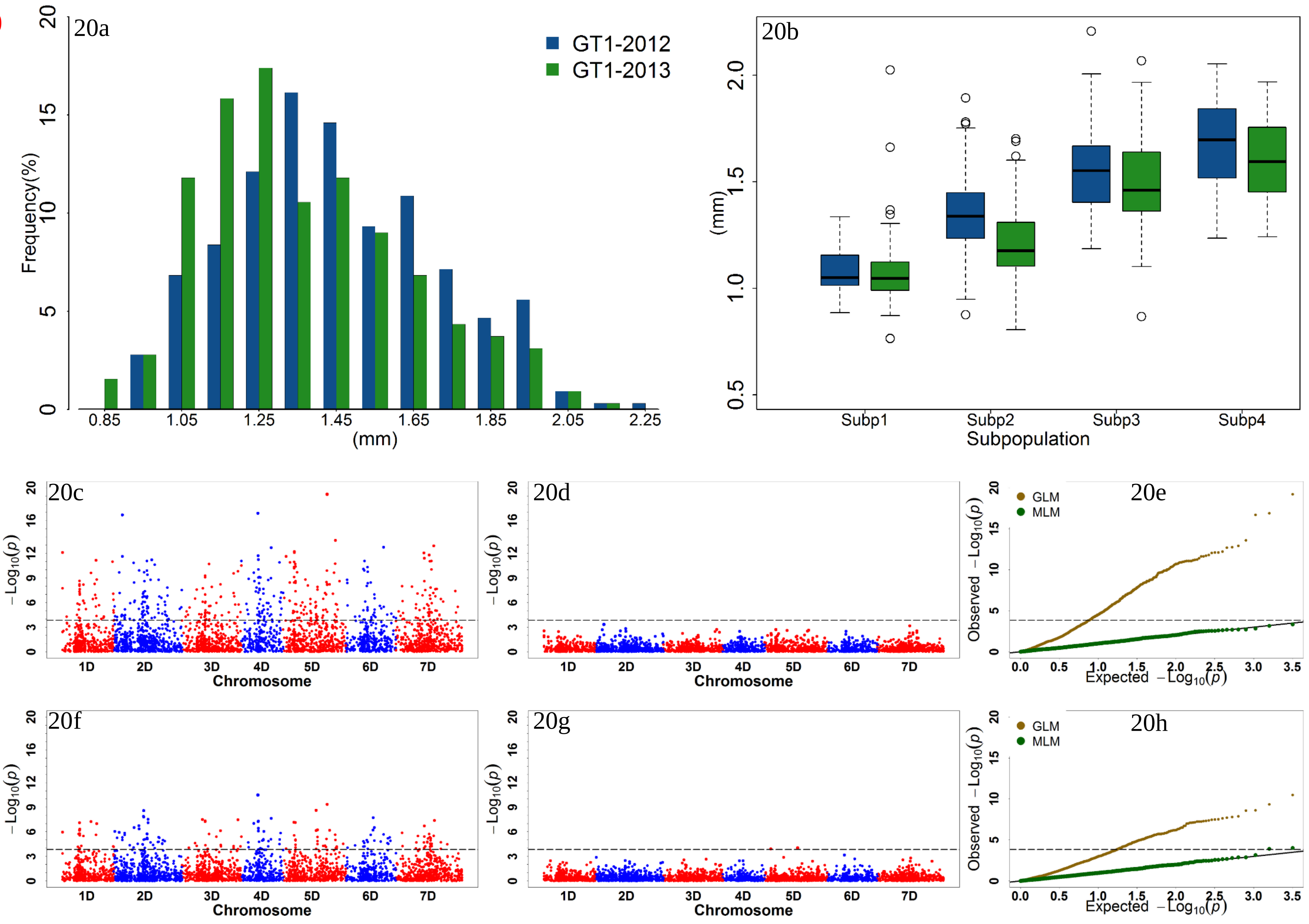
20a
GT1-2012
GT1-2013
Frequency(%)
(mm)
0.85
1.05
1.25
1.45
1.65
1.85
2.05
2.25
20b
(mm)
Subp1
Subp2
Subp3
Subp4
Subpopulation
20c
20d
20e
GLM
MLM
Chromosome
1D
2D
3D
4D
5D
6D
7D
Observed
Expected
20f
20g
20h

21

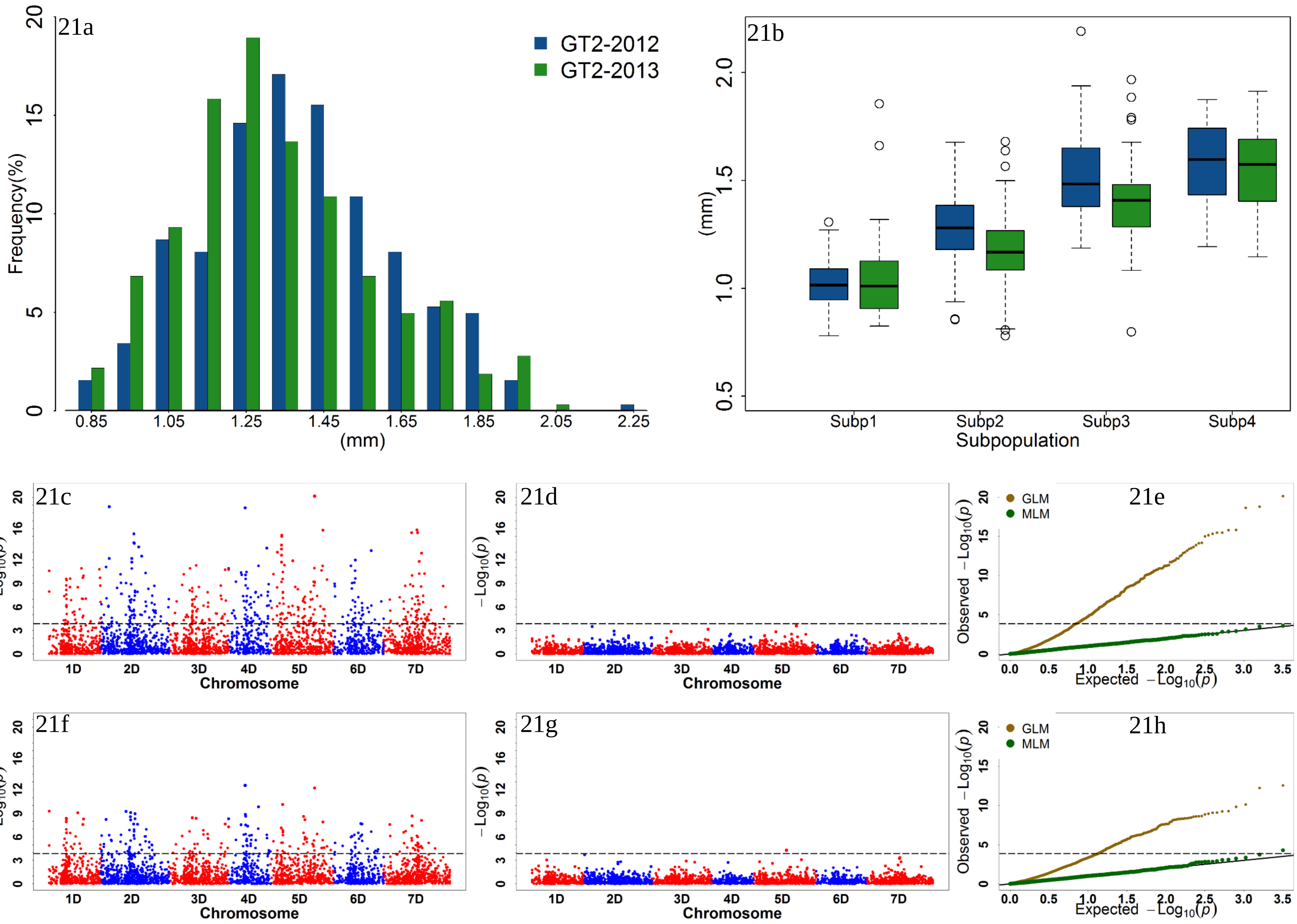
21a
GT2-2012
GT2-2013
Frequency(%)
(mm)
0.85
1.05
1.25
1.45
1.65
1.85
2.05
2.25
21b
(mm)
Subp1
Subp2
Subp3
Subp4
Subpopulation
21c
21d
21e
21f
21g
21h
GLM
MLM
$-\mathrm{Log}_{10}(p)$
Chromosome
1D
2D
3D
4D
5D
6D
7D
Observed $-\mathrm{Log}_{10}(p)$
Expected $-\mathrm{Log}_{10}(p)$

22

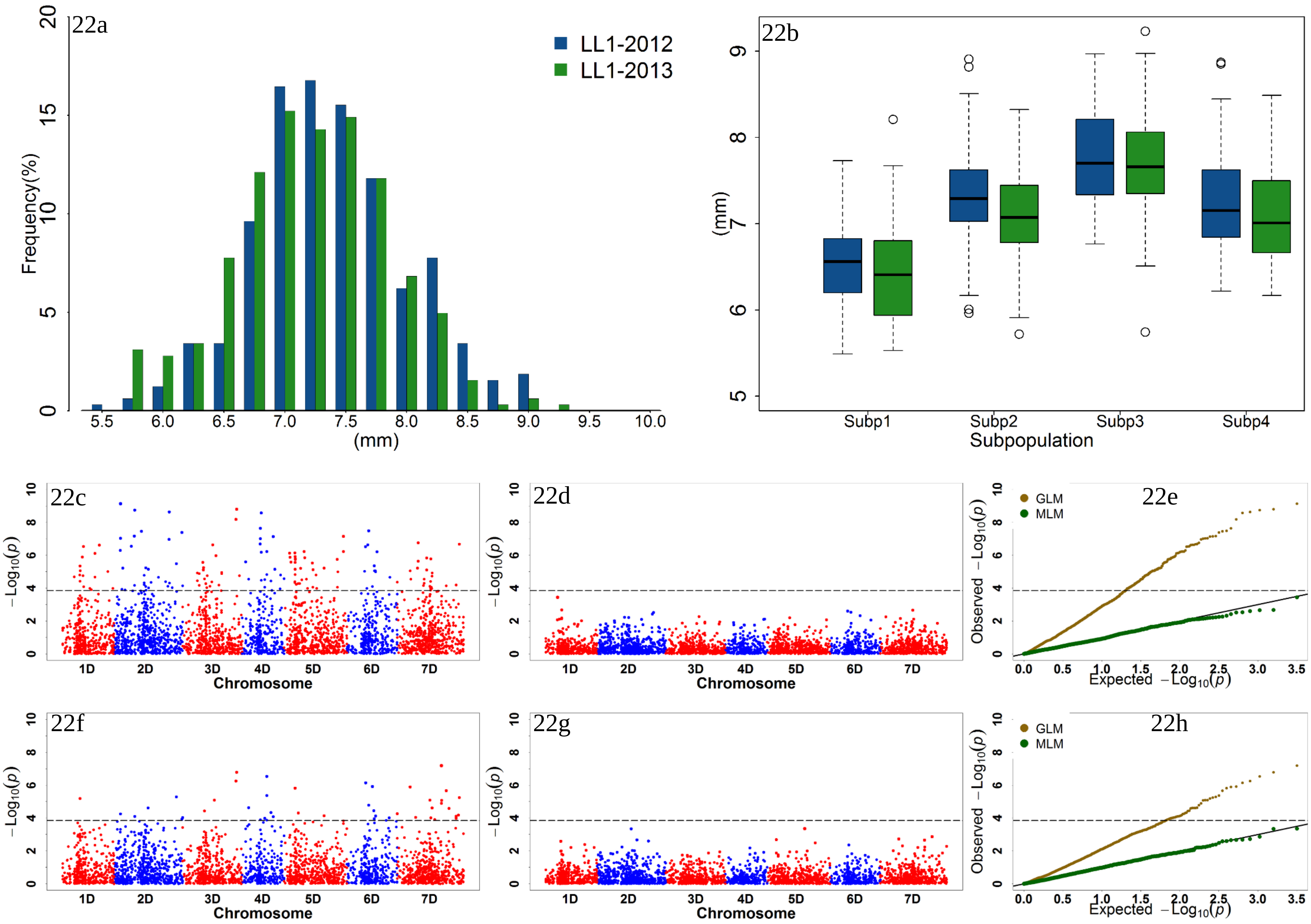
22a
LL1-2012
LL1-2013
Frequency(%)
(mm)
22b
(mm)
Subp1
Subp2
Subp3
Subp4
Subpopulation
22c
22d
22e
22f
22g
22h
$-\log_{10}(p)$
Chromosome
1D
2D
3D
4D
5D
6D
7D
GLM
MLM
Observed $-\log_{10}(p)$
Expected $-\log_{10}(p)$

23

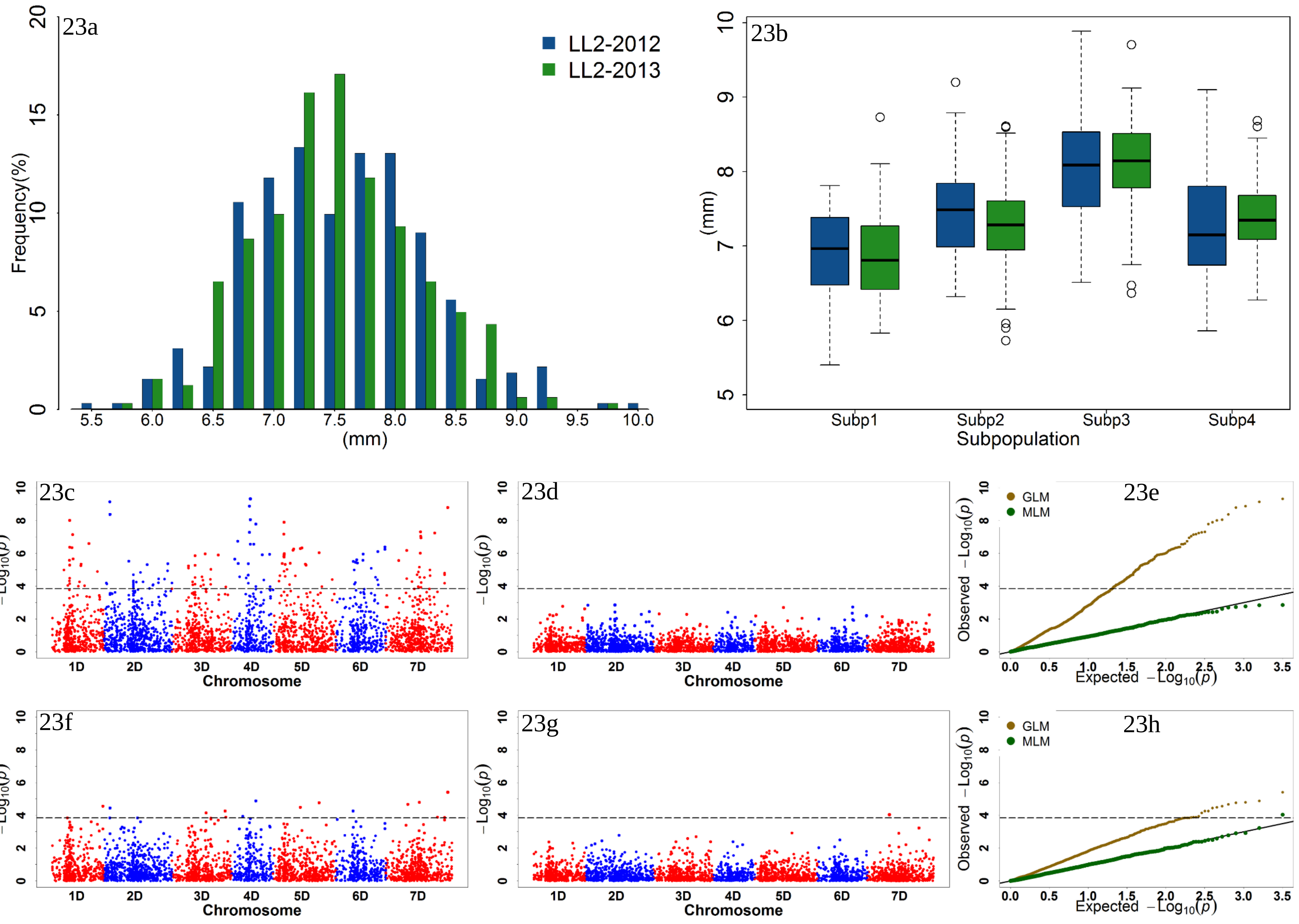

23a
LL2-2012
LL2-2013
Frequency(%)
(mm)
23b
(mm)
Subp1
Subp2
Subp3
Subp4
Subpopulation
23c
23d
23e
GLM
MLM
Chromosome
1D
2D
3D
4D
5D
6D
7D
Observed −Log10(p)
Expected −Log10(p)
23f
23g
23h

24

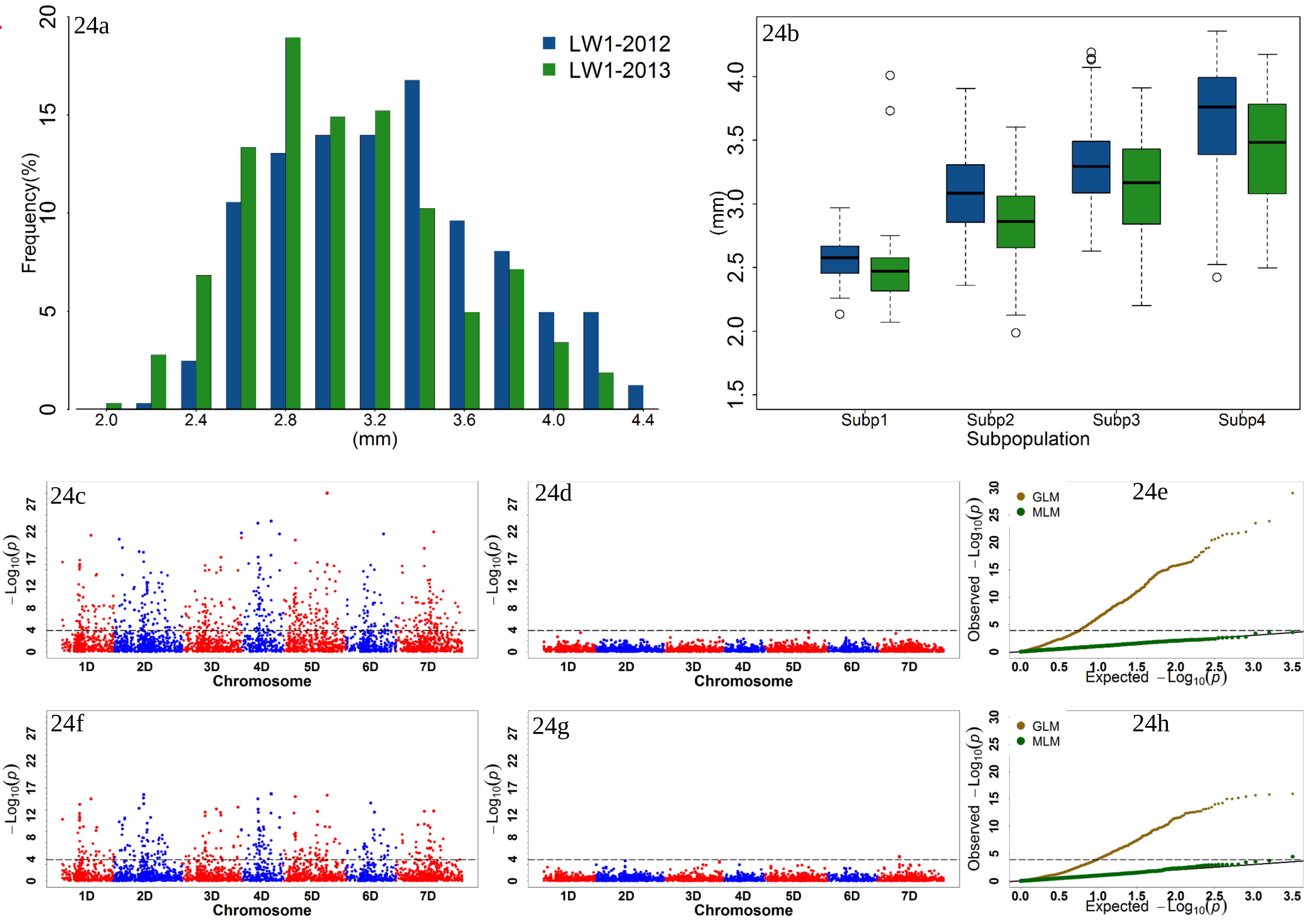
24a
LW1-2012
LW1-2013
Frequency(%)
(mm)
24b
(mm)
Subp1
Subp2
Subp3
Subp4
Subpopulation
24c
24d
24e
24f
24g
24h
Chromosome
GLM
MLM
Observed $-\log_{10}(p)$
Expected $-\log_{10}(p)$

25

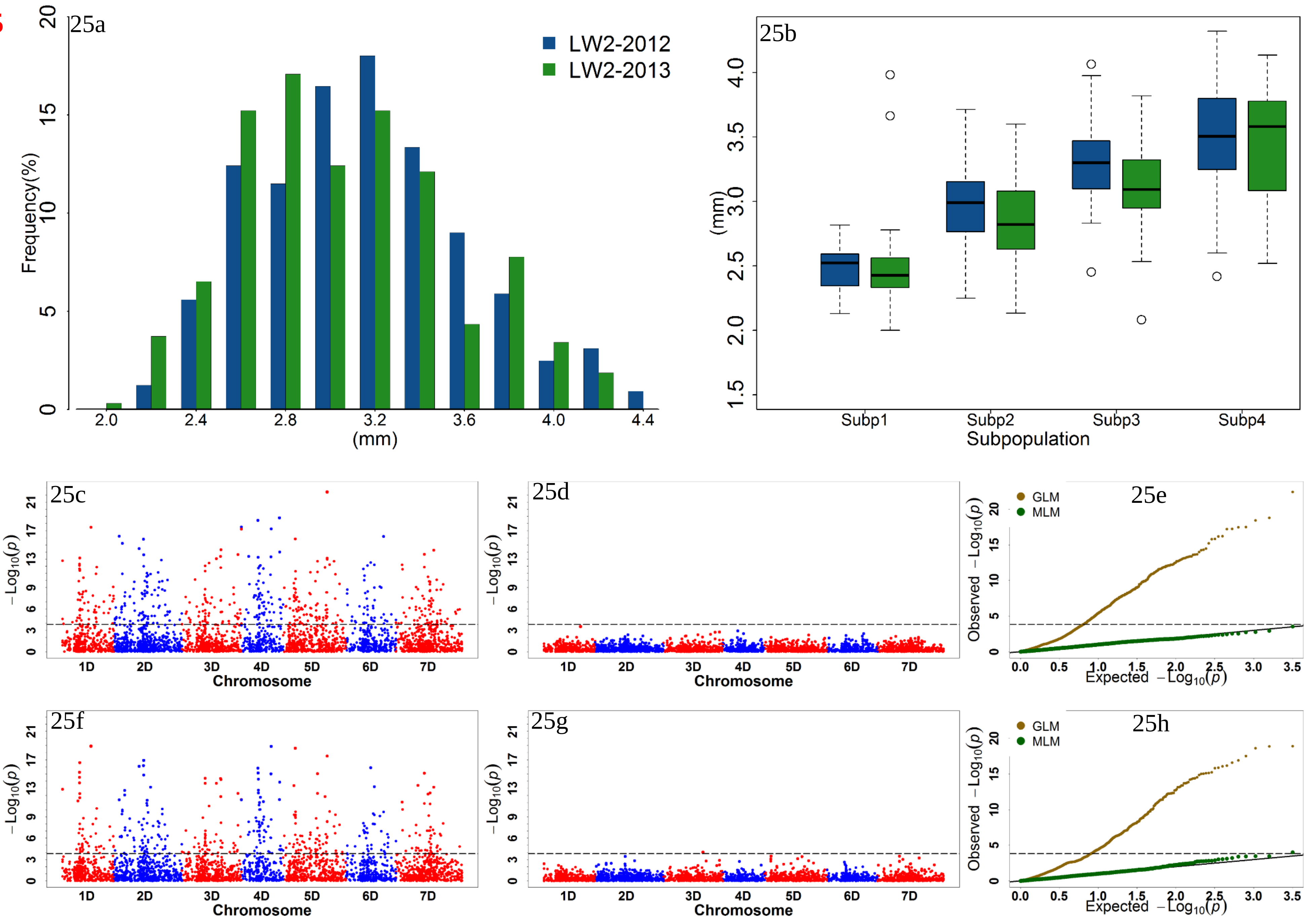
25a
LW2-2012
LW2-2013
Frequency(%)
(mm)
25b
(mm)
Subp1
Subp2
Subp3
Subp4
Subpopulation
25c
25d
25e
GLM
MLM
Chromosome
Observed −Log10(p)
Expected −Log10(p)
25f
25g
25h

26

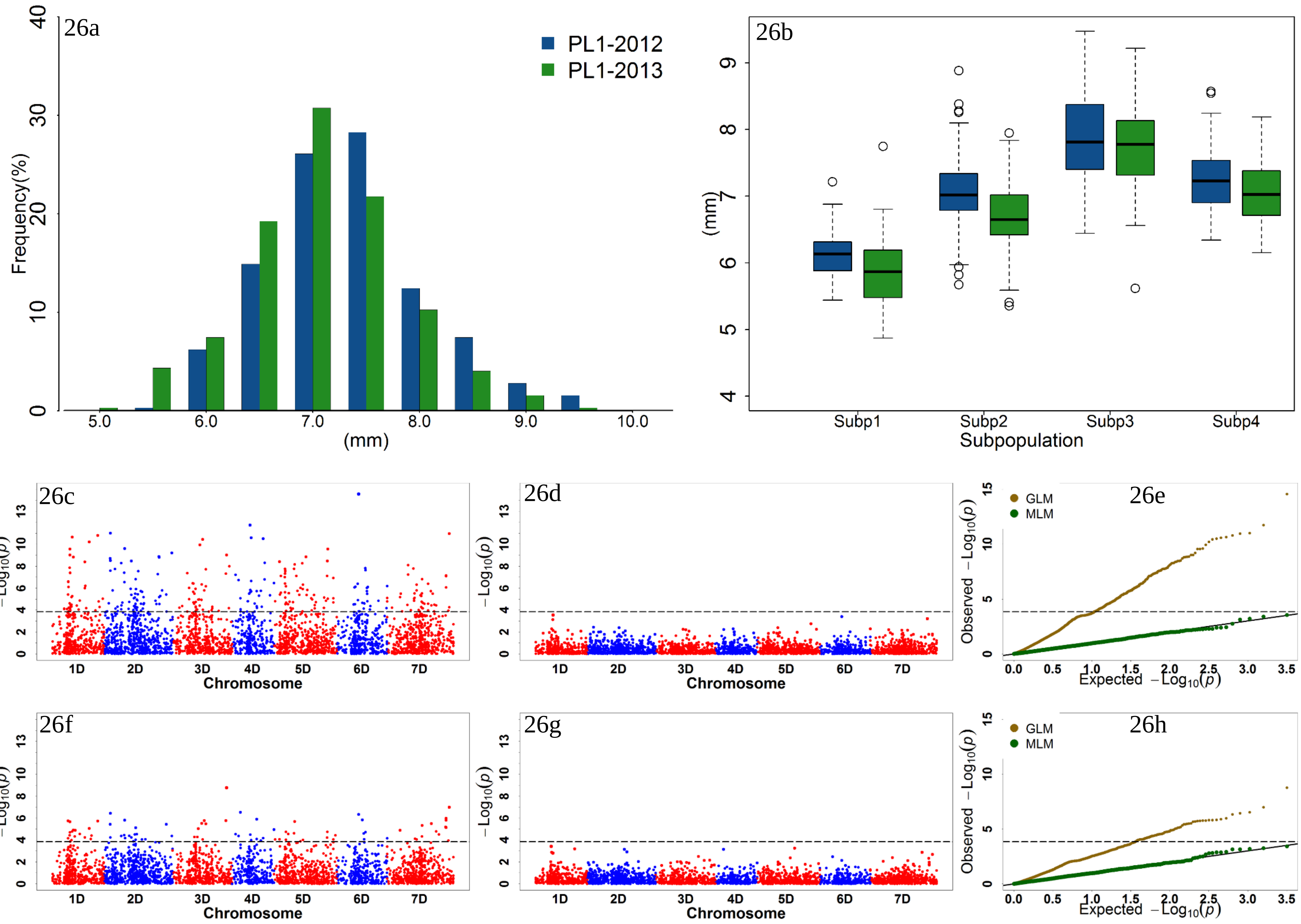
26a
PL1-2012
PL1-2013
Frequency(%)
(mm)
26b
(mm)
Subp1
Subp2
Subp3
Subp4
Subpopulation
26c
26d
26e
26f
26g
26h
Chromosome
1D
2D
3D
4D
5D
6D
7D
GLM
MLM
Observed −Log10(p)
Expected −Log10(p)

27

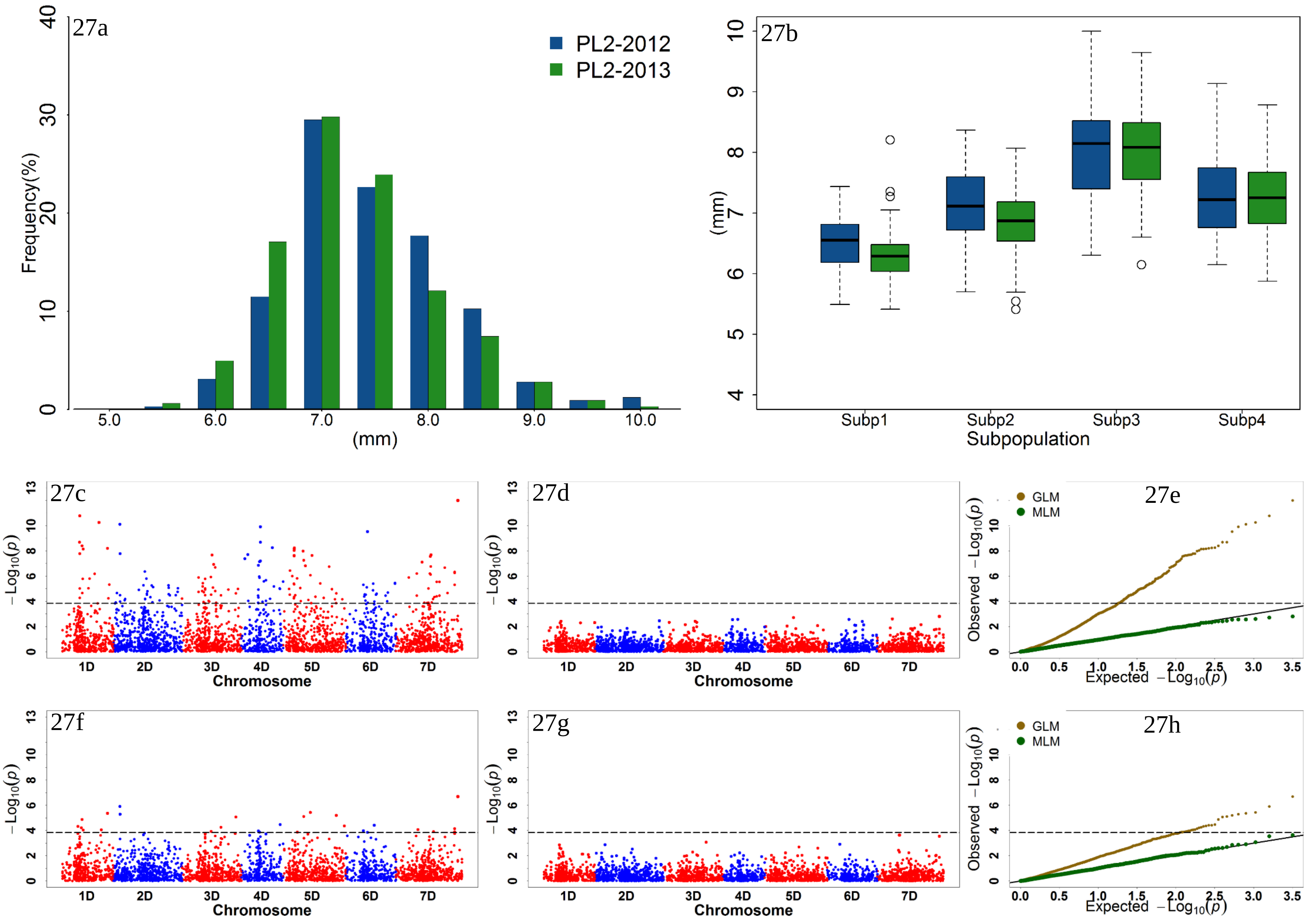

27a
PL2-2012
PL2-2013
Frequency(%)
(mm)
27b
(mm)
Subp1
Subp2
Subp3
Subp4
Subpopulation
27c
27d
27e
27f
27g
27h
Chromosome
1D
2D
3D
4D
5D
6D
7D
GLM
MLM
Observed $-\mathrm{Log}_{10}(p)$
Expected $-\mathrm{Log}_{10}(p)$

28

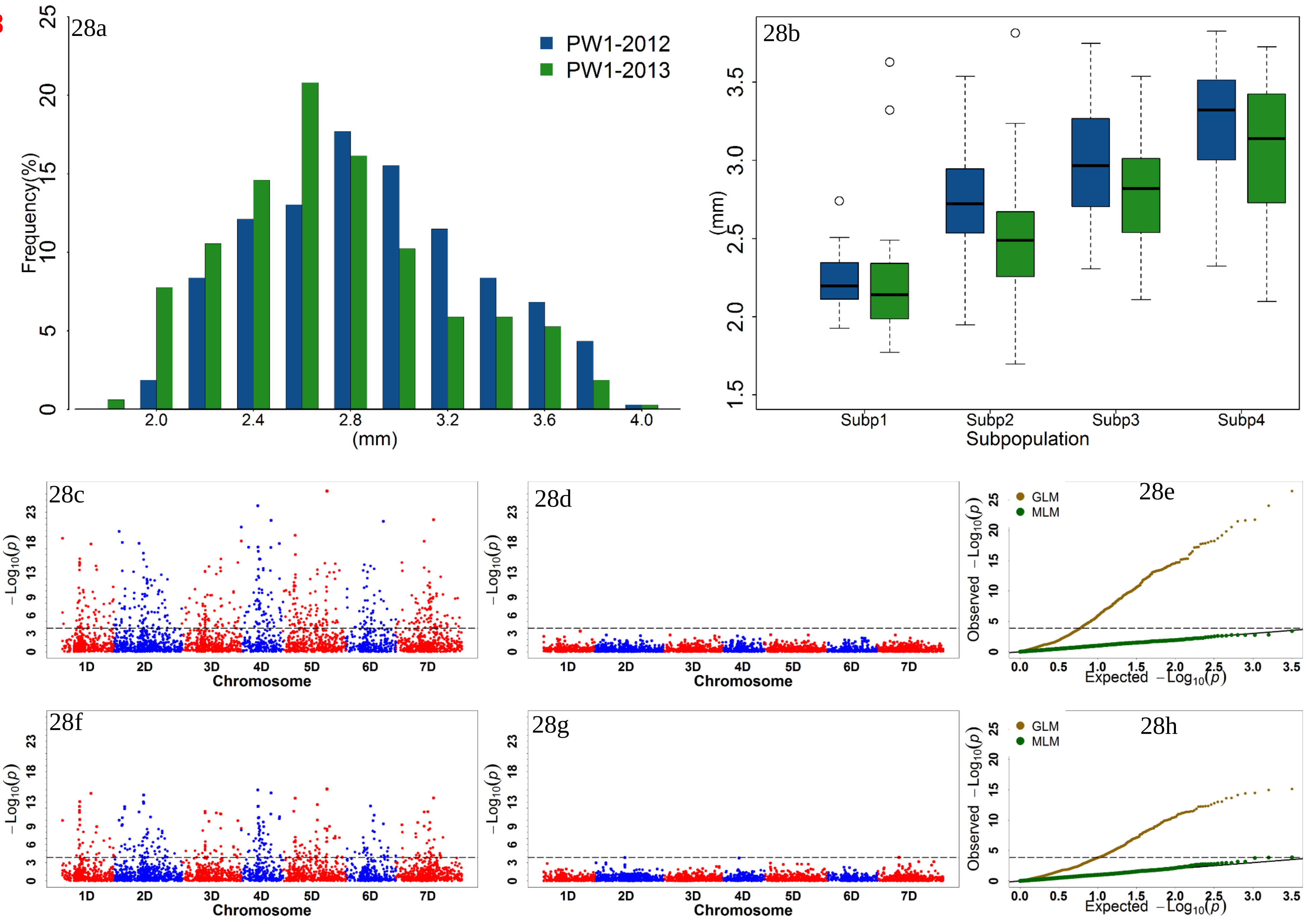
28a
PW1-2012
PW1-2013
Frequency(%)
(mm)
28b
(mm)
Subp1
Subp2
Subp3
Subp4
Subpopulation
28c
28d
28e
28f
28g
28h
Chromosome
1D
2D
3D
4D
5D
6D
7D
GLM
MLM
Observed −Log10(p)
Expected −Log10(p)

29

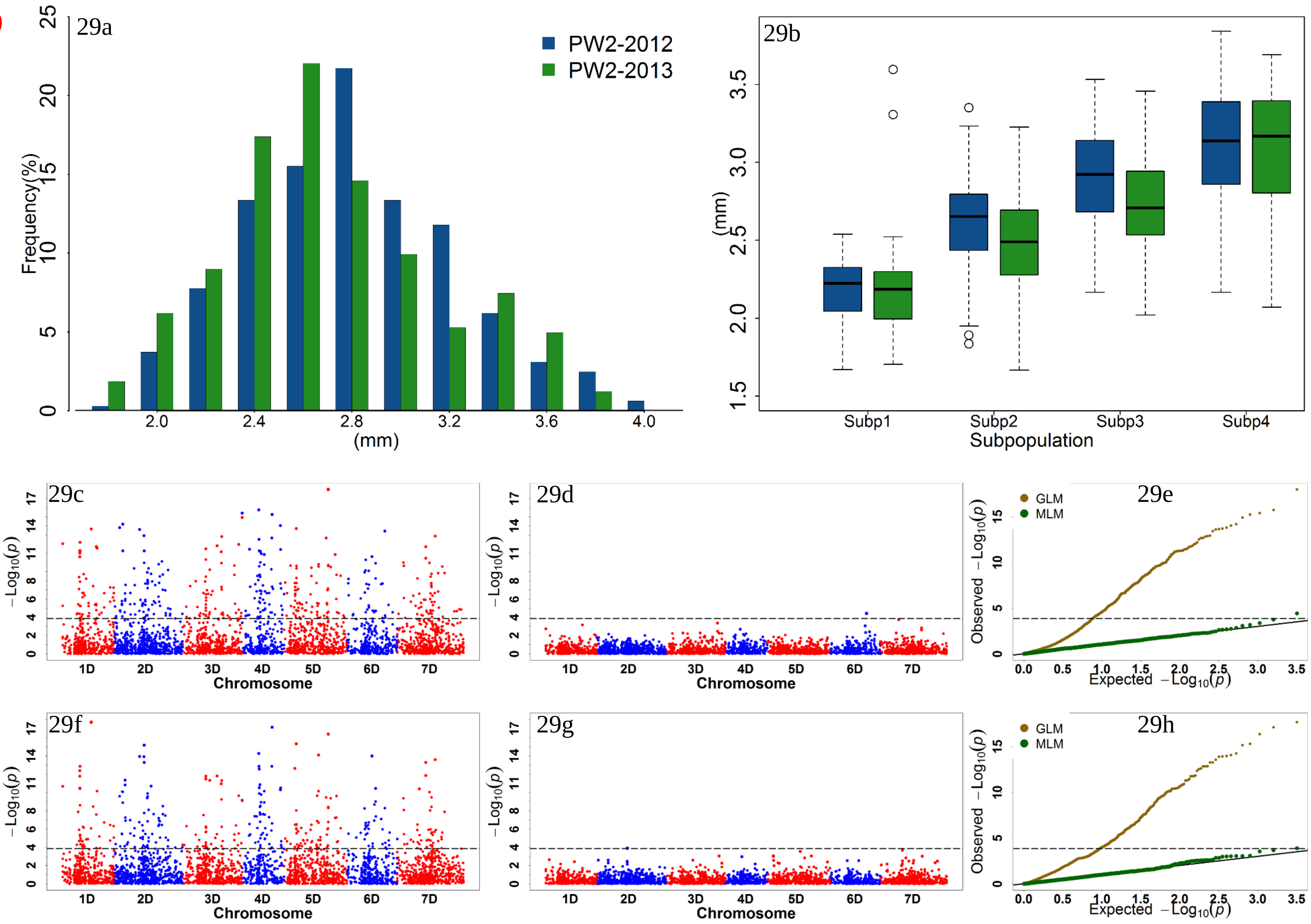
29a
PW2-2012
PW2-2013
Frequency(%)
0
5
10
15
20
25
2.0
2.4
2.8
3.2
3.6
4.0
(mm)
29b
(mm)
1.5
2.0
2.5
3.0
3.5
Subp1
Subp2
Subp3
Subp4
Subpopulation
29c
29d
29f
29g
$-\mathrm{Log}_{10}(p)$
0
2
4
6
8
11
14
17
1D
2D
3D
4D
5D
6D
7D
Chromosome
29e
29h
GLM
MLM
Observed $-\mathrm{Log}_{10}(p)$
Expected $-\mathrm{Log}_{10}(p)$
0.0
0.5
1.0
1.5
2.0
2.5
3.0
3.5

1: Summary of GWAS results for plant height (PH). (1a, 1b) Phenotype histogram and distribution of subpopulation in year 2012 and 2013. (1c, 1d) GLM model results for association of year 2012. (1e) Q-Q plots of GLM and MLM model in year 2012. (1f, 1g) MLM model results for association of year 2013. (1h) Q-Q plots of GLM and MLM model in year 2013.

2: Summary of GWAS results for spike length (SL). (2a, 2b) Phenotype histogram and distribution of subpopulation in year 2012 and 2013. (2c, 2d) GLM model results for association of year 2012. (2e) Q-Q plots of GLM and MLM model in year 2012. (2f, 2g) MLM model results for association of year 2013. (2h) Q-Q plots of GLM and MLM model in year 2013.

3: Summary of GWAS results for internode length 1 (IL1). (3a, 3b) Phenotype histogram and distribution of subpopulation in year 2012 and 2013. (3c, 3d) GLM model results for association of year 2012. (3e) Q-Q plots of GLM and MLM model in year 2012. (3f, 3g) MLM model results for association of year 2013. (3h) Q-Q plots of GLM and MLM model in year 2013.

4: Summary of GWAS results for internode length 2 (IL2). (4a, 4b) Phenotype histogram and distribution of subpopulation in year 2012 and 2013. (4c, 4d) GLM model results for association of year 2012. (4e) Q-Q plots of GLM and MLM model in year 2012. (4f, 4g) MLM model results for association of year 2013. (4h) Q-Q plots of GLM and MLM model in year 2013.

5: Summary of GWAS results for internode length 3 (IL3). (5a, 5b) Phenotype histogram and distribution of subpopulation in year 2012 and 2013. (5c, 5d) GLM model results for association of year 2012. (5e) Q-Q plots of GLM and MLM model in year 2012. (5f, 5g) MLM model results for association of year 2013. (5h) Q-Q plots of GLM and MLM model in year 2013.

6: Summary of GWAS results for internode length 4 (IL4). (6a, 6b) Phenotype histogram and distribution of subpopulation in year 2012 and 2013. (6c, 6d) GLM model results for association of year 2012. (6e) Q-Q plots of GLM and MLM model in year 2012. (6f, 6g) MLM model results for association of year 2013. (6h) Q-Q plots of GLM and MLM model in year 2013.

7: Summary of GWAS results for flag leaf length (FL). (7a, 7b) Phenotype histogram and

distribution of subpopulation in year 2012 and 2013. (7c, 7d) GLM model results for association of year 2012. (7e) Q-Q plots of GLM and MLM model in year 2012. (7f, 7g) MLM model results for association of year 2013. (7h) Q-Q plots of GLM and MLM model in year 2013.

8: Summary of GWAS results for flag leaf width (FW). (8a, 8b) Phenotype histogram and distribution of subpopulation in year 2012 and 2013. (8c, 8d) GLM model results for association of year 2012. (8e) Q-Q plots of GLM and MLM model in year 2012. (8f, 8g) MLM model results for association of year 2013. (8h) Q-Q plots of GLM and MLM model in year 2013.

9: Summary of GWAS results for leaf numbers (LN). (9a, 9b) Phenotype histogram and distribution of subpopulation in year 2012 and 2013. (9c, 9d) GLM model results for association of year 2012. (9e) Q-Q plots of GLM and MLM model in year 2012. (9f, 9g) MLM model results for association of year 2013. (9h) Q-Q plots of GLM and MLM model in year 2013.

10: Summary of GWAS results for stem node numbers (SNN). (10a, 10b) Phenotype histogram and distribution of subpopulation in year 2012 and 2013. (10c, 10d) GLM model results for association of year 2012. (10e) Q-Q plots of GLM and MLM model in year 2012. (10f, 10g) MLM model results for association of year 2013. (10h) Q-Q plots of GLM and MLM model in year 2013.

11: Summary of GWAS results for awn length 1 (AL1). (11a, 11b) Phenotype histogram and distribution of subpopulation in year 2012 and 2013. (11c, 11d) GLM model results for association of year 2012. (11e) Q-Q plots of GLM and MLM model in year 2012. (11f, 11g) MLM model results for association of year 2013. (11h) Q-Q plots of GLM and MLM model in year 2013.

12: Summary of GWAS results for awn length 1 (AL2). (12a, 12b) Phenotype histogram and distribution of subpopulation in year 2012 and 2013. (12c, 12d) GLM model results for association of year 2012. (12e) Q-Q plots of GLM and MLM model in year 2012. (12f, 12g) MLM model results for association of year 2013. (12h) Q-Q plots of GLM and MLM model in year 2013.

13: Summary of GWAS results for spikelet length (SPL). (13a, 13b) Phenotype histogram and distribution of subpopulation in year 2012 and 2013. (13c, 13d) GLM model results for association of year 2012. (13e) Q-Q plots of GLM and MLM model in year 2012. (13f, 13g) MLM model results for

association of year 2013. (13h) Q-Q plots of GLM and MLM model in year 2013.

14: Summary of GWAS results for spikelet width (SPW). (14a, 14b) Phenotype histogram and distribution of subpopulation in year 2012 and 2013. (14c, 14d) GLM model results for association of year 2012. (14e) Q-Q plots of GLM and MLM model in year 2012. (14f, 14g) MLM model results for association of year 2013. (14h) Q-Q plots of GLM and MLM model in year 2013.

15: Summary of GWAS results for spikelet numbers (SPN). (15a, 15b) Phenotype histogram and distribution of subpopulation in year 2012 and 2013. (15c, 15d) GLM model results for association of year 2012. (15e) Q-Q plots of GLM and MLM model in year 2012. (15f, 15g) MLM model results for association of year 2013. (15h) Q-Q plots of GLM and MLM model in year 2013.

16: Summary of GWAS results for glume length 1 (GL1). (16a, 16b) Phenotype histogram and distribution of subpopulation in year 2012 and 2013. (16c, 16d) GLM model results for association of year 2012. (16e) Q-Q plots of GLM and MLM model in year 2012. (16f, 16g) MLM model results for association of year 2013. (16h) Q-Q plots of GLM and MLM model in year 2013.

17: Summary of GWAS results for glume length 2 (GL2). (17a, 17b) Phenotype histogram and distribution of subpopulation in year 2012 and 2013. (17c, 17d) GLM model results for association of year 2012. (17e) Q-Q plots of GLM and MLM model in year 2012. (17f, 17g) MLM model results for association of year 2013. (17h) Q-Q plots of GLM and MLM model in year 2013.

18: Summary of GWAS results for glume width 1 (GW1). (18a, 18b) Phenotype histogram and distribution of subpopulation in year 2012 and 2013. (18c, 18d) GLM model results for association of year 2012. (18e) Q-Q plots of GLM and MLM model in year 2012. (18f, 18g) MLM model results for association of year 2013. (18h) Q-Q plots of GLM and MLM model in year 2013.

19: Summary of GWAS results for glume width 2 (GW2). (19a, 19b) Phenotype histogram and distribution of subpopulation in year 2012 and 2013. (19c, 19d) GLM model results for association of year 2012. (19e) Q-Q plots of GLM and MLM model in year 2012. (19f, 19g) MLM model results for association of year 2013. (19h) Q-Q plots of GLM and MLM model in year 2013.

20: Summary of GWAS results for glume thickness 1 (GT1). (20a, 20b) Phenotype histogram and distribution of subpopulation in year 2012 and 2013. (20c, 20d) GLM model results for association of year 2012. (20e) Q-Q plots of GLM and MLM model in year 2012. (20f, 20g) MLM model results for association of year 2013. (20h) Q-Q plots of GLM and MLM model in year 2013.

21: Summary of GWAS results for glume thickness 2 (GT2). (21a, 21b) Phenotype histogram and distribution of subpopulation in year 2012 and 2013. (21c, 21d) GLM model results for association of year 2012. (21e) Q-Q plots of GLM and MLM model in year 2012. (21f, 21g) MLM model results for association of year 2013. (21h) Q-Q plots of GLM and MLM model in year 2013.

22: Summary of GWAS results for lemma length 1 (LL1). (22a, 22b) Phenotype histogram and distribution of subpopulation in year 2012 and 2013. (22c, 22d) GLM model results for association of year 2012. (22e) Q-Q plots of GLM and MLM model in year 2012. (22f, 22g) MLM model results for association of year 2013. (22h) Q-Q plots of GLM and MLM model in year 2013.

23: Summary of GWAS results for lemma length 2 (LL2). (23a, 23b) Phenotype histogram and distribution of subpopulation in year 2012 and 2013. (23c, 23d) GLM model results for association of year 2012. (23e) Q-Q plots of GLM and MLM model in year 2012. (23f, 23g) MLM model results for association of year 2013. (23h) Q-Q plots of GLM and MLM model in year 2013.

24: Summary of GWAS results for lemma width 1 (LW1). (24a, 24b) Phenotype histogram and distribution of subpopulation in year 2012 and 2013. (24c, 24d) GLM model results for association of year 2012. (24e) Q-Q plots of GLM and MLM model in year 2012. (24f, 24g) MLM model results for association of year 2013. (24h) Q-Q plots of GLM and MLM model in year 2013.

25: Summary of GWAS results for lemma length 2 (LW2). (25a, 25b) Phenotype histogram and distribution of subpopulation in year 2012 and 2013. (25c, 25d) GLM model results for association of year 2012. (25e) Q-Q plots of GLM and MLM model in year 2012. (25f, 25g) MLM model results for association of year 2013. (25h) Q-Q plots of GLM and MLM model in year 2013.

26: Summary of GWAS results for palea length 1 (PL1). (26a, 26b) Phenotype histogram and distribution of subpopulation in year 2012 and 2013. (26c, 26d) GLM model results for association of

year 2012. (26e) Q-Q plots of GLM and MLM model in year 2012. (26f, 26g) MLM model results for association of year 2013. (26h) Q-Q plots of GLM and MLM model in year 2013.

27: Summary of GWAS results for palea length 2 (PL1). (27a, 27b) Phenotype histogram and distribution of subpopulation in year 2012 and 2013. (27c, 27d) GLM model results for association of year 2012. (27e) Q-Q plots of GLM and MLM model in year 2012. (27f, 27g) MLM model results for association of year 2013. (27h) Q-Q plots of GLM and MLM model in year 2013.

28: Summary of GWAS results for palea width 1 (PW1). (28a, 28b) Phenotype histogram and distribution of subpopulation in year 2012 and 2013. (28c, 28d) GLM model results for association of year 2012. (28e) Q-Q plots of GLM and MLM model in year 2012. (28f, 28g) MLM model results for association of year 2013. (28h) Q-Q plots of GLM and MLM model in year 2013.

29: Summary of GWAS results for palea width 2 (PW2). (29a, 29b) Phenotype histogram and distribution of subpopulation in year 2012 and 2013. (29c, 29d) GLM model results for association of year 2012. (29e) Q-Q plots of GLM and MLM model in year 2012. (29f, 29g) MLM model results for association of year 2013. (29h) Q-Q plots of GLM and MLM model in year 2013.

Supplementary Fig. S2 1 to 29 Phenotypic best linear unbiased predictor (BLUP) distribution and genome-wide association scan for all BLUP of tested traits. (a) A histogram showing the distribution of BLUP of each phenotype in the *Aegilops tauschii* diversity panel. (b) Boxplot showing the mean, median and range of phenotypic BLUP in each *Aegilops tauschii* subpopulation independently. (c) Quantile-Quantile (Q-Q) plot showing the expected null distribution of *p* values, assuming no associations, represented as a solid black line; distribution of *p* values observed using the general linear model (GLM) represented as a dark goldenrod plot; distribution of *p* values observed using mixed linear model (MLM) represented as a dark green plot. (d) A Manhattan plot for the seven chromosomes carrying the significant markers detected by GLM and MLM models, *p* value is converted into $-\log_{10}(p)$, threshold of 3.84 are indicated by horizontal dashed lines.

1

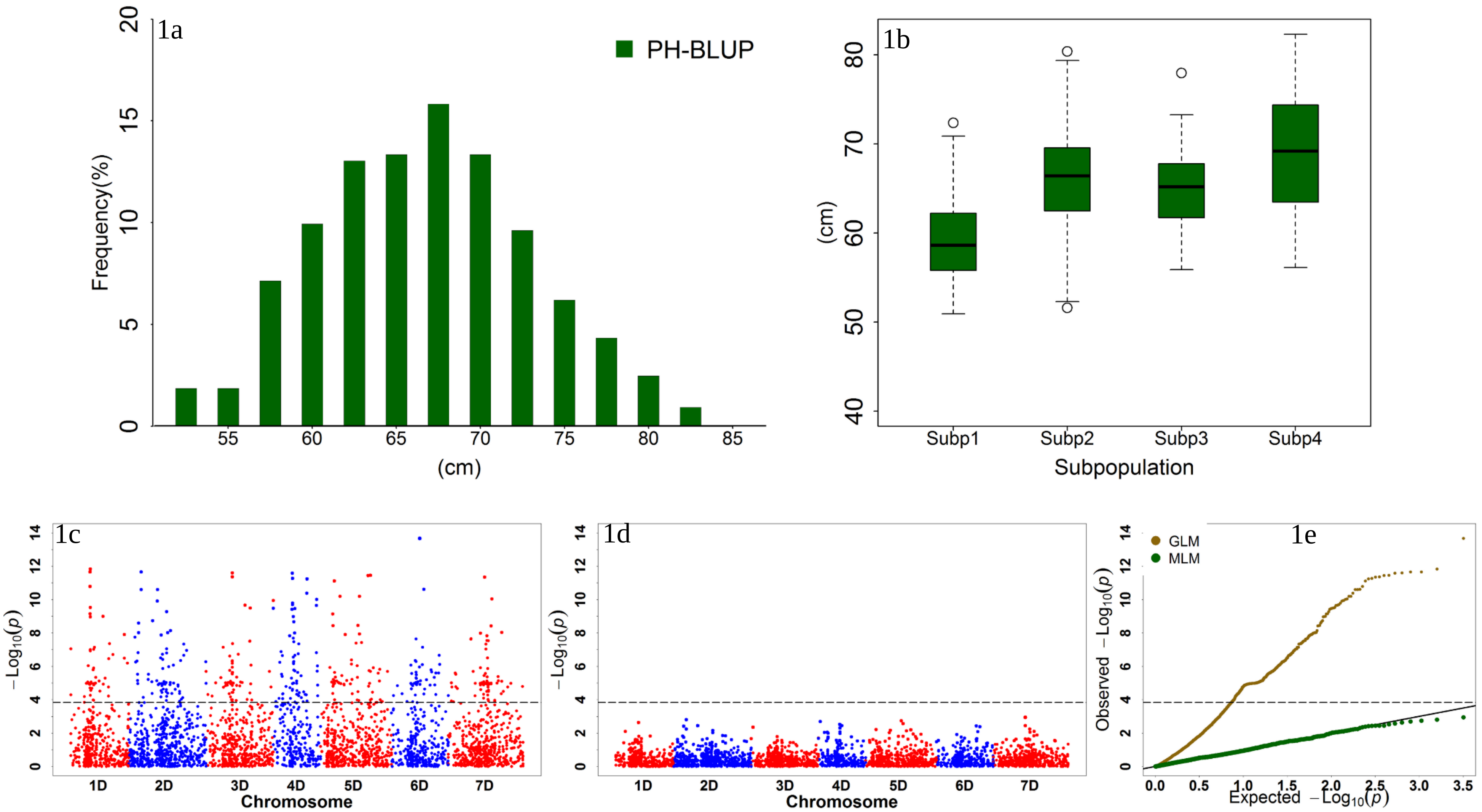

1: Summary of GWAS results for BLUP of plant height (PH-BLUP). (1a, 1b) Phenotypic BLUP histogram, distribution of each subpopulation. (1c) GLM results for association analysis. (1d) MLM results for association analysis. (1e) Q-Q plots of GLM and MLM.

2

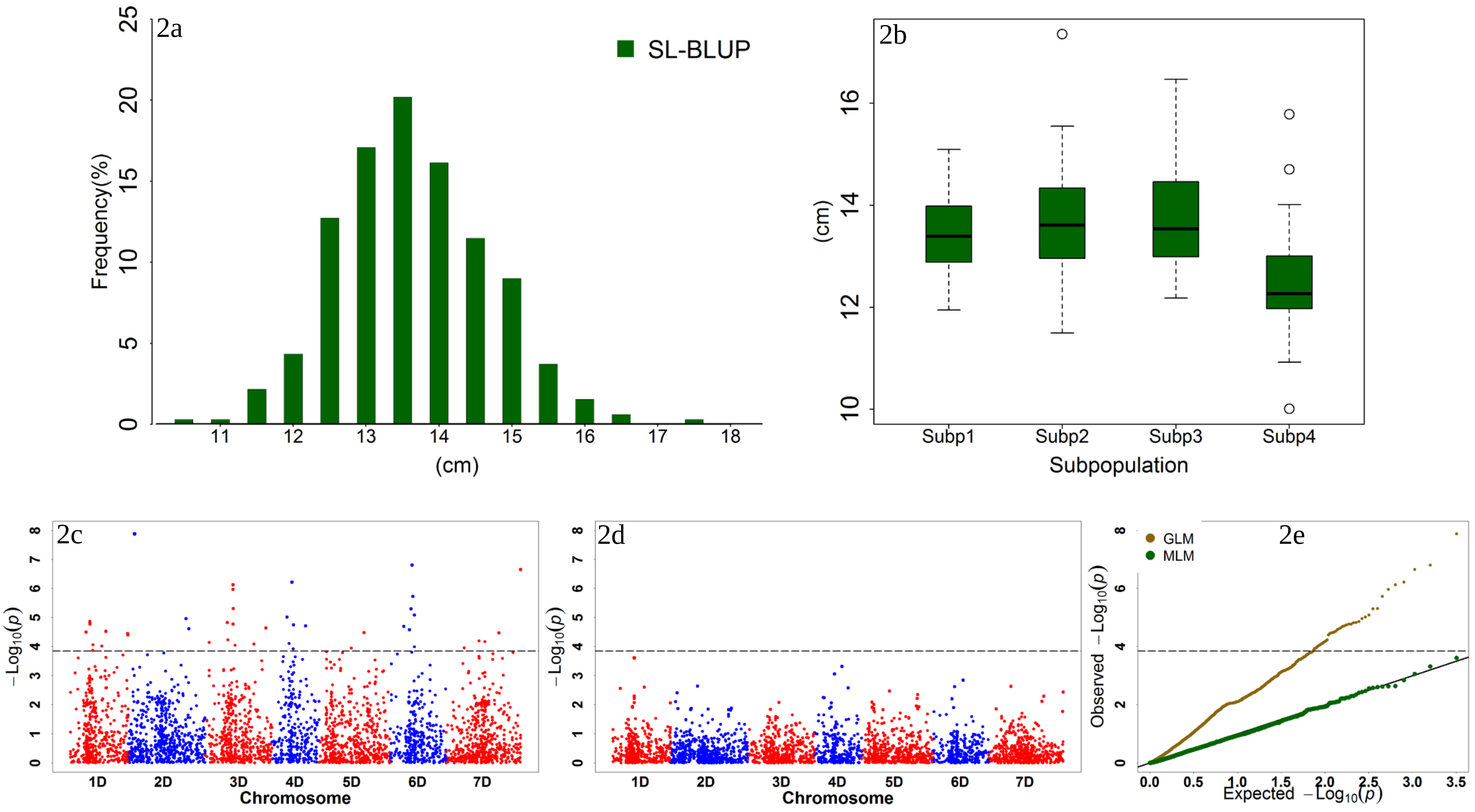

2: Summary of GWAS results for BLUP of spike length (SL-BLUP). (2a, 2b) Phenotypic BLUP histogram, distribution of each subpopulation. (2c) GLM results for association analysis. (2d) MLM results for association analysis. (2e) Q-Q plots of GLM and MLM.

3

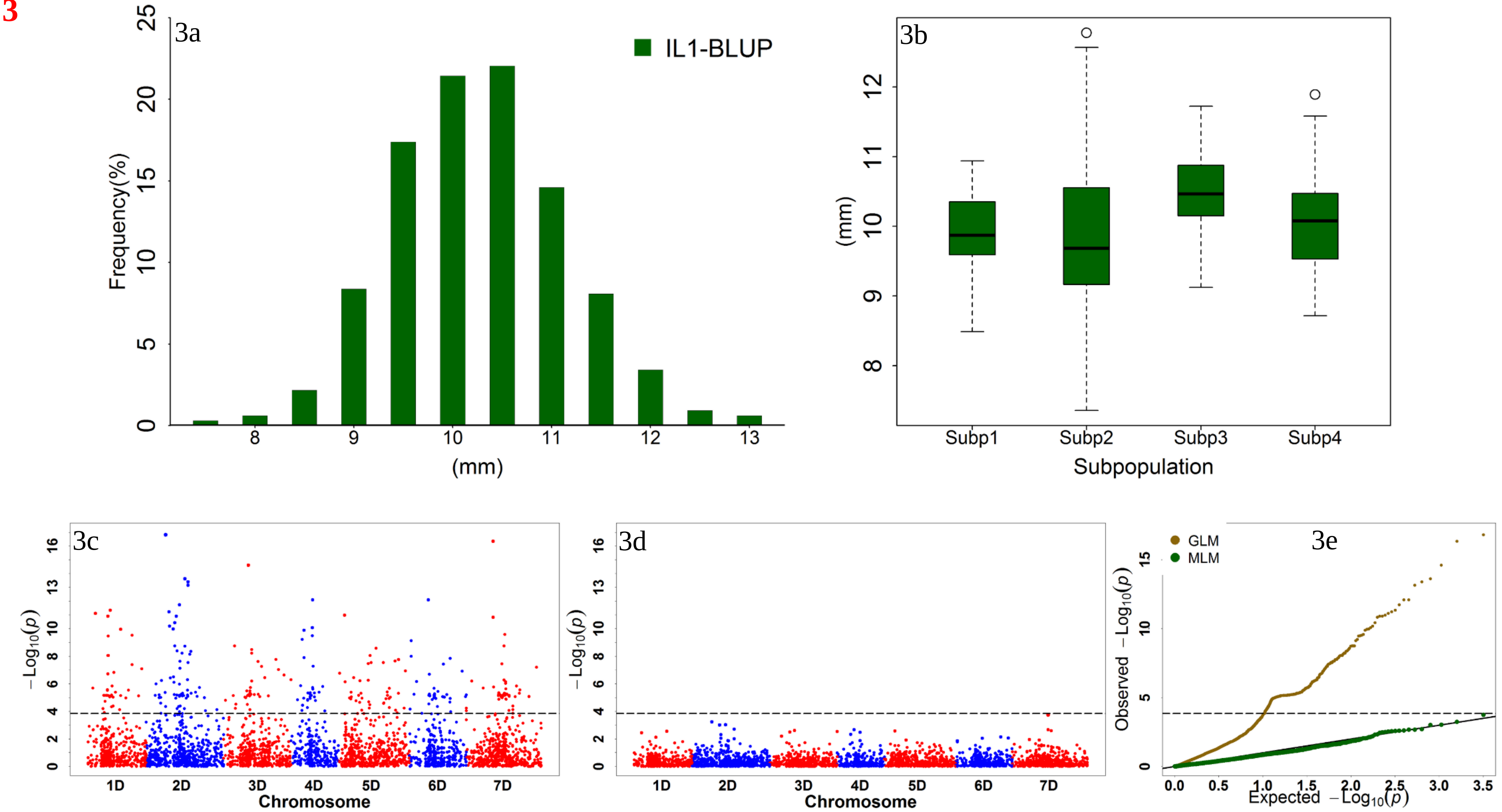

3: Summary of GWAS results for BLUP of internode length 1 (IL1-BLUP). (3a, 3b) Phenotypic BLUP histogram, distribution of each subpopulation. (3c) GLM results for association analysis. (3d) MLM results for association analysis. (3e) Q-Q plots of GLM and MLM.

4

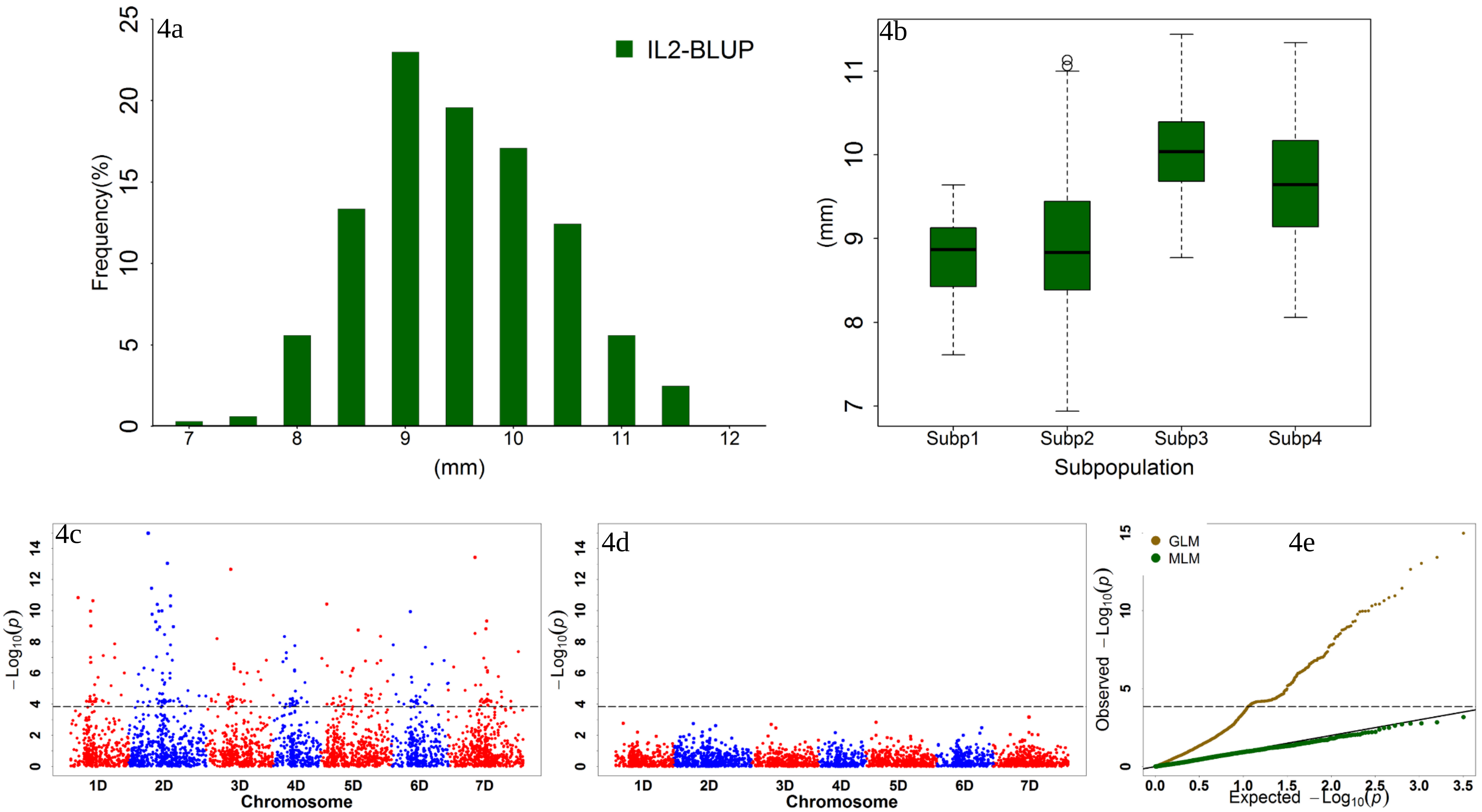

4: Summary of GWAS results for BLUP of internode length 2 (IL2-BLUP). (4a, 4b) Phenotypic BLUP histogram, distribution of each subpopulation. (4c) GLM results for association analysis. (4d) MLM results for association analysis. (4e) Q-Q plots of GLM and MLM.

5

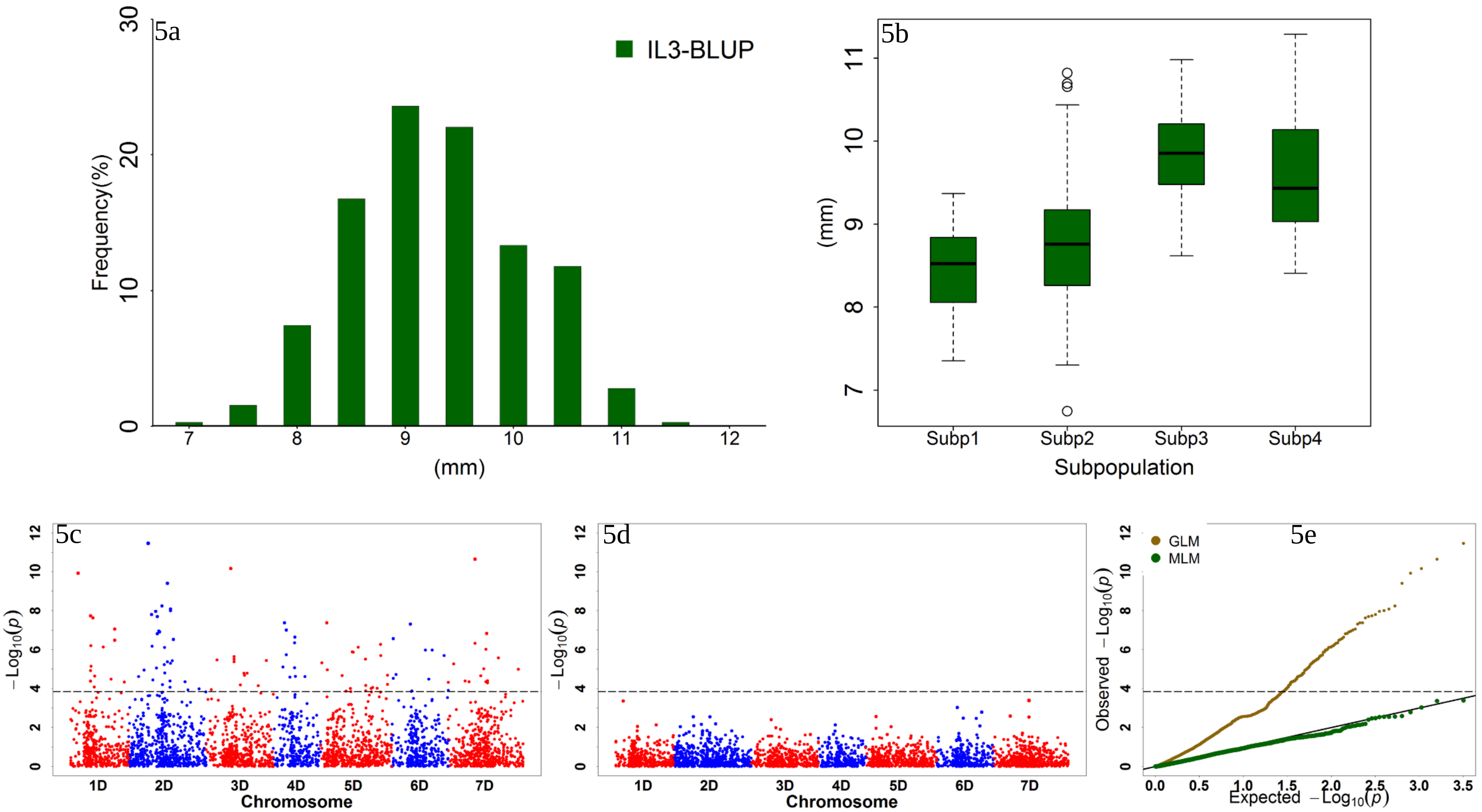

5: Summary of GWAS results for BLUP of internode length 3 (IL3-BLUP). (5a, 5b) Phenotypic BLUP histogram, distribution of each subpopulation. (5c) GLM results for association analysis. (5d) MLM results for association analysis. (5e) Q-Q plots of GLM and MLM.

6

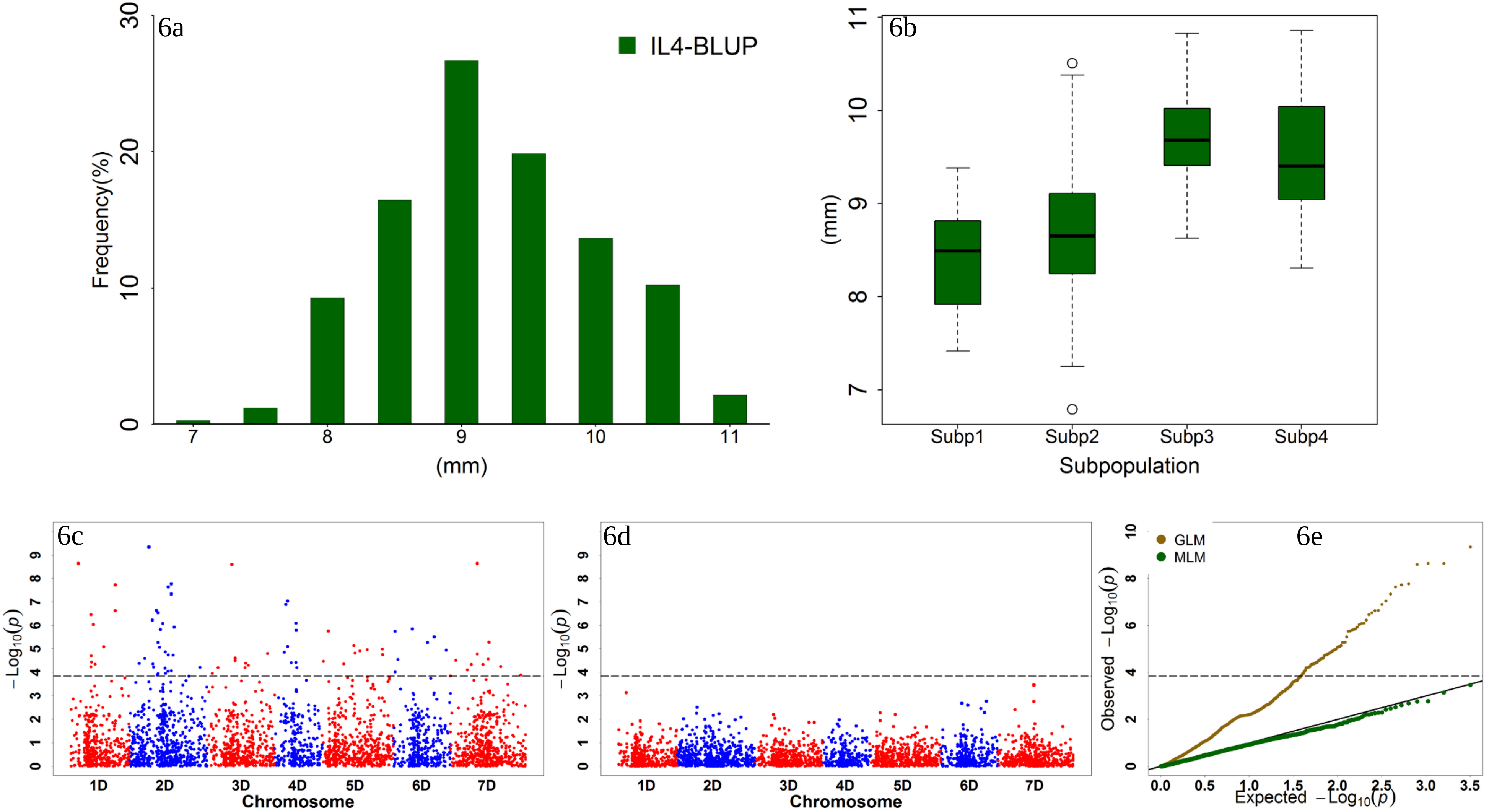

6: Summary of GWAS results for BLUP of internode length 4 (IL4-BLUP). (6a, 6b) Phenotypic BLUP histogram, distribution of each subpopulation. (6c) GLM results for association analysis. (6d) MLM results for association analysis. (6e) Q-Q plots of GLM and MLM.

7

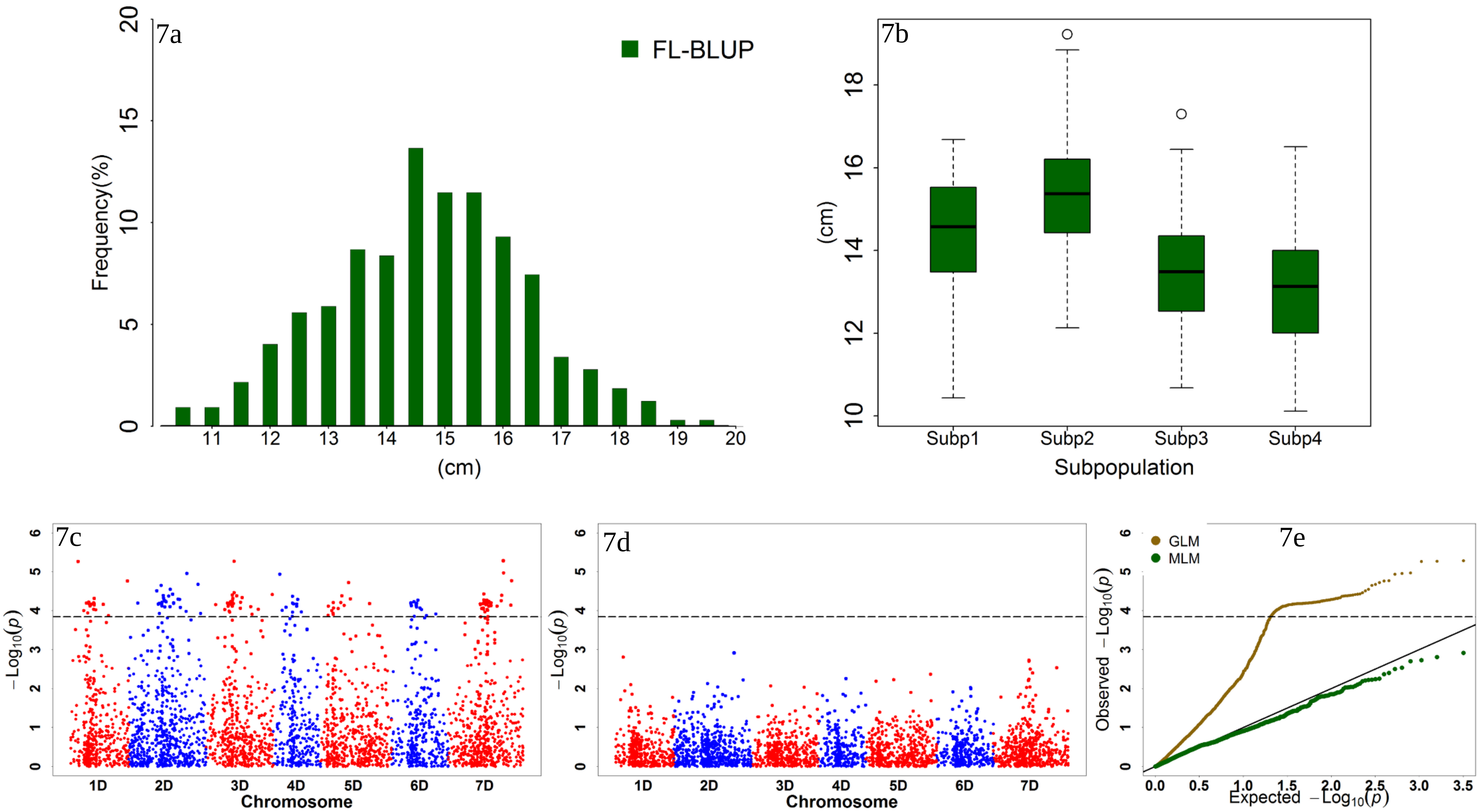

7: Summary of GWAS results for BLUP of flag leaf length (FL-BLUP). (7a, 7b) Phenotypic BLUP histogram, distribution of each subpopulation. (7c) GLM results for association analysis. (7d) MLM results for association analysis. (7e) Q-Q plots of GLM and MLM.

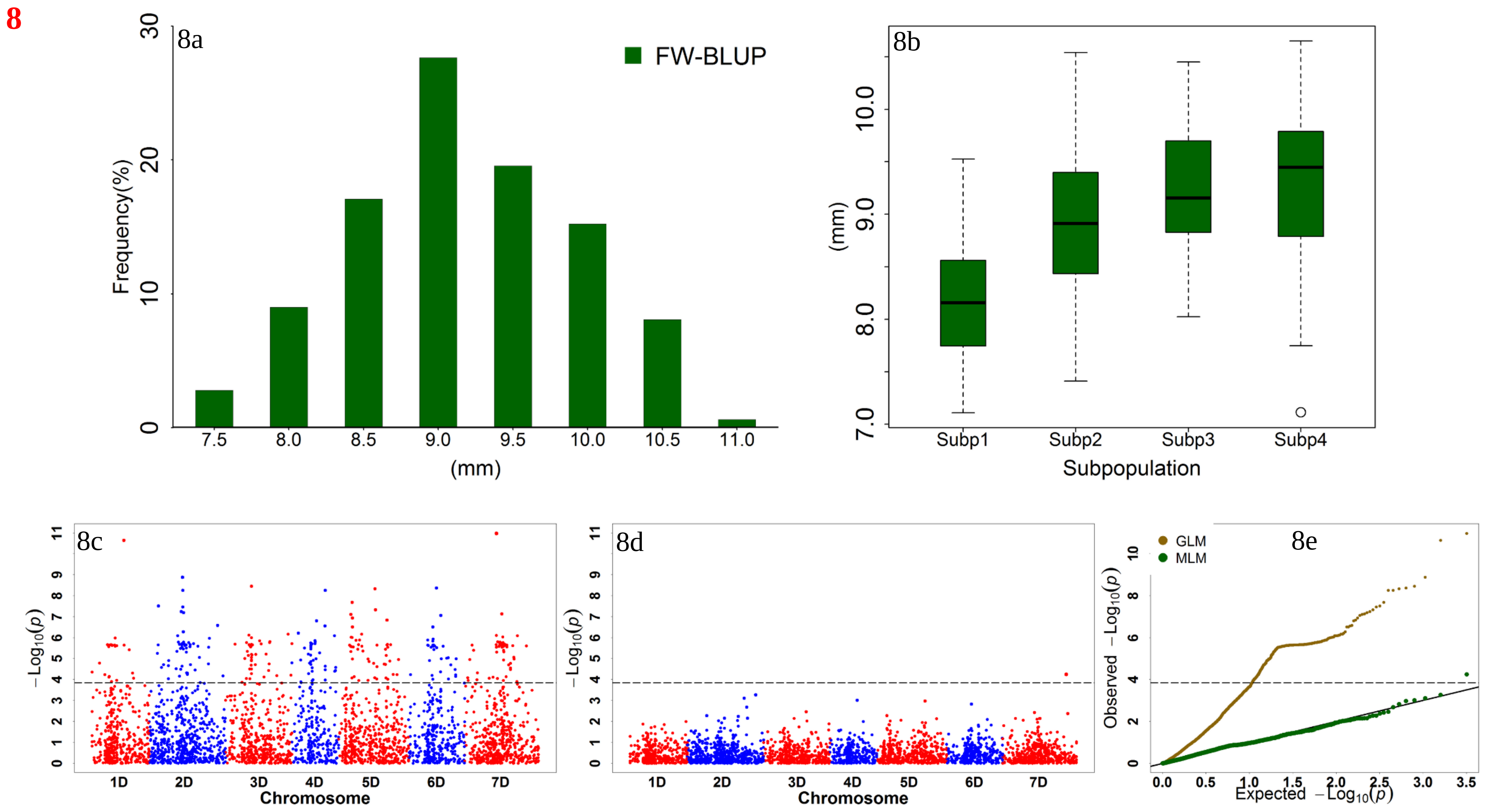

8: Summary of GWAS results for BLUP of flag leaf width (FW-BLUP). (8a, 8b) Phenotypic BLUP histogram, distribution of each subpopulation. (8c) GLM results for association analysis. (8d) MLM results for association analysis. (8e) Q-Q plots of GLM and MLM.

9

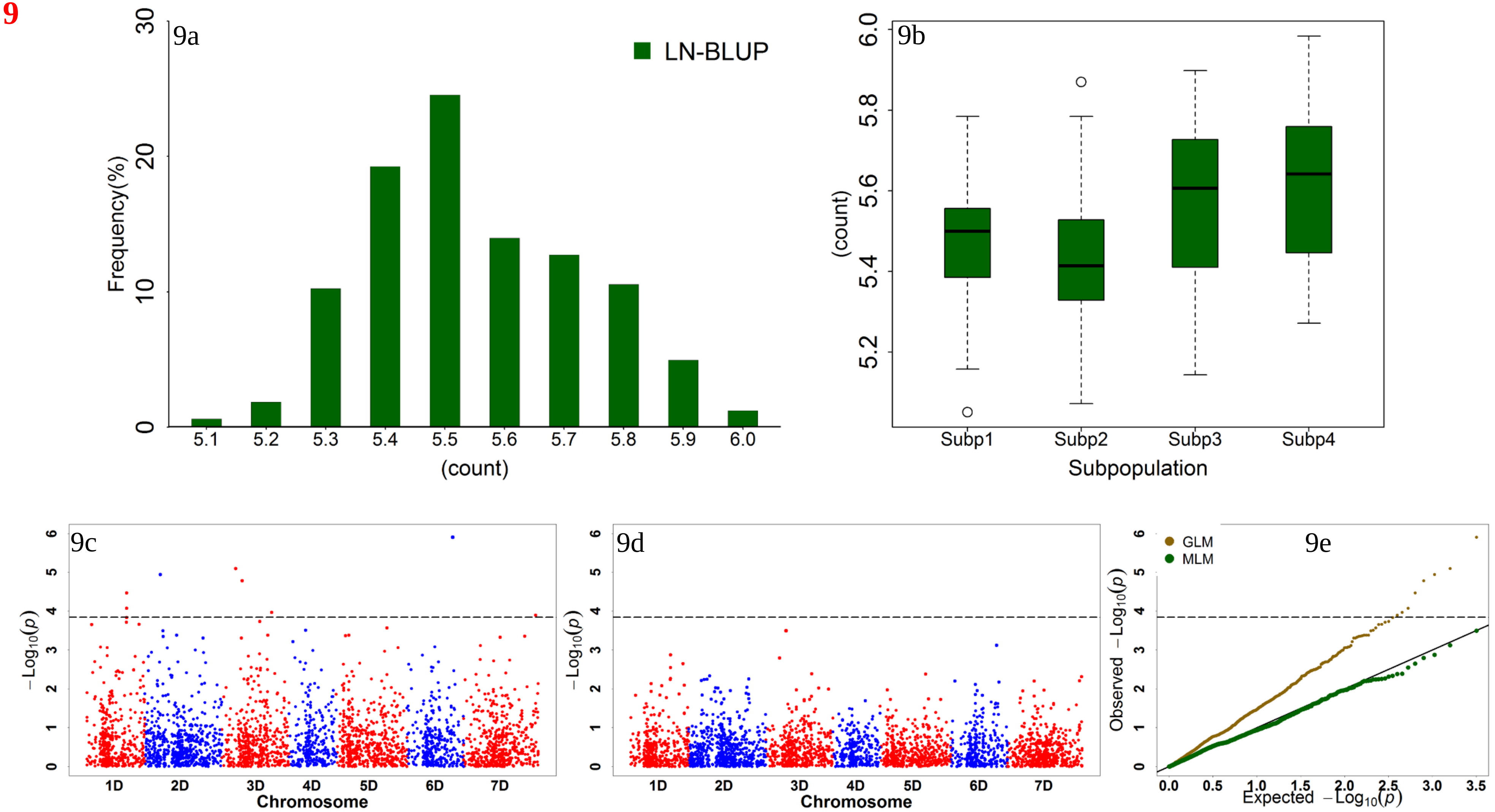

9: Summary of GWAS results for BLUP of leaf numbers (LN-BLUP). (9a, 9b) Phenotypic BLUP histogram, distribution of each subpopulation. (9c) GLM results for association analysis. (9d) MLM results for association analysis. (9e) Q-Q plots of GLM and MLM.

10

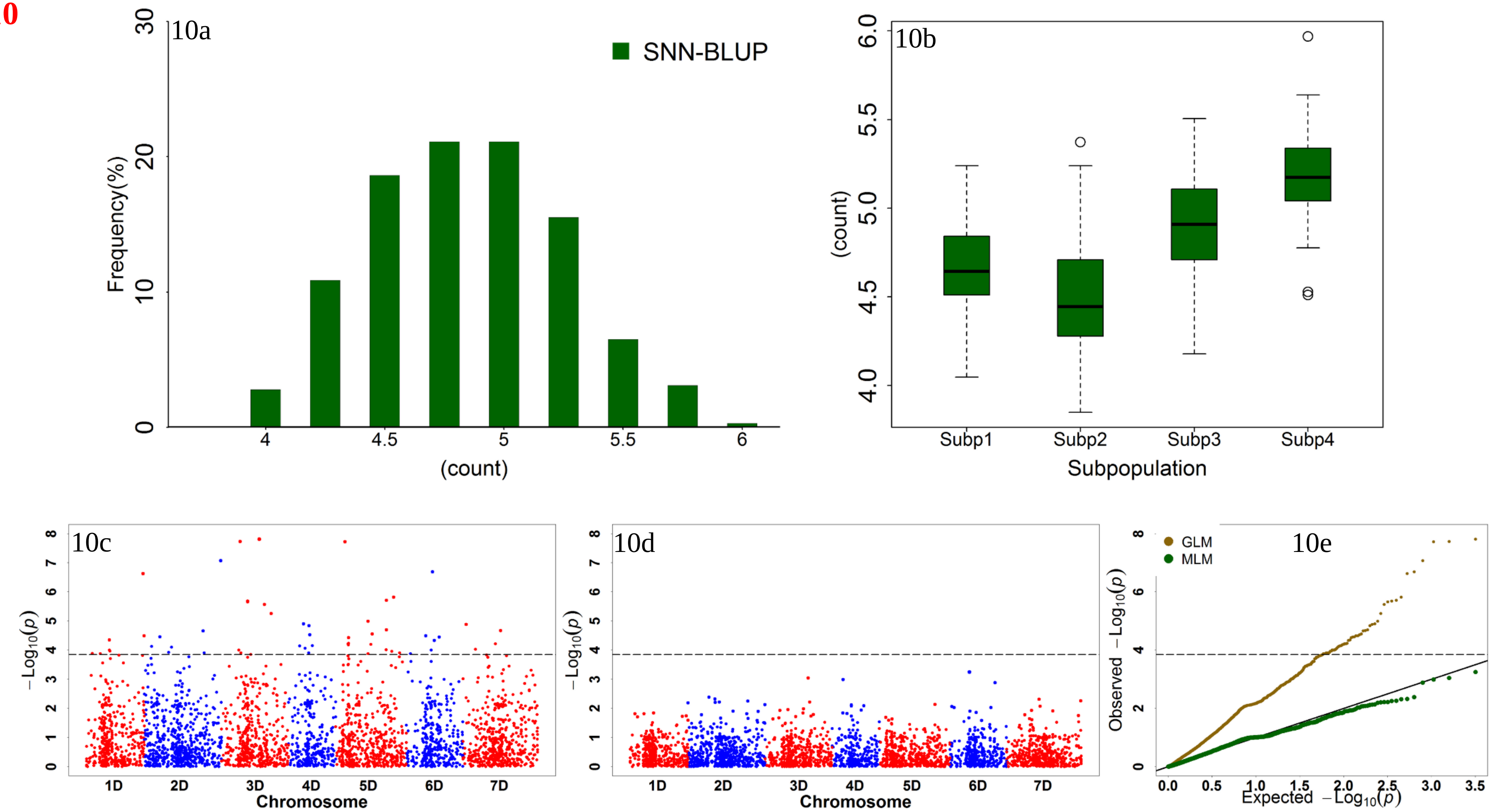

10: Summary of GWAS results for BLUP of stem node numbers (SNN-BLUP). (10a, 10b) Phenotypic BLUP histogram, distribution of each subpopulation. (10c) GLM results for association analysis. (10d) MLM results for association analysis. (10e) Q-Q plots of GLM and MLM.

11

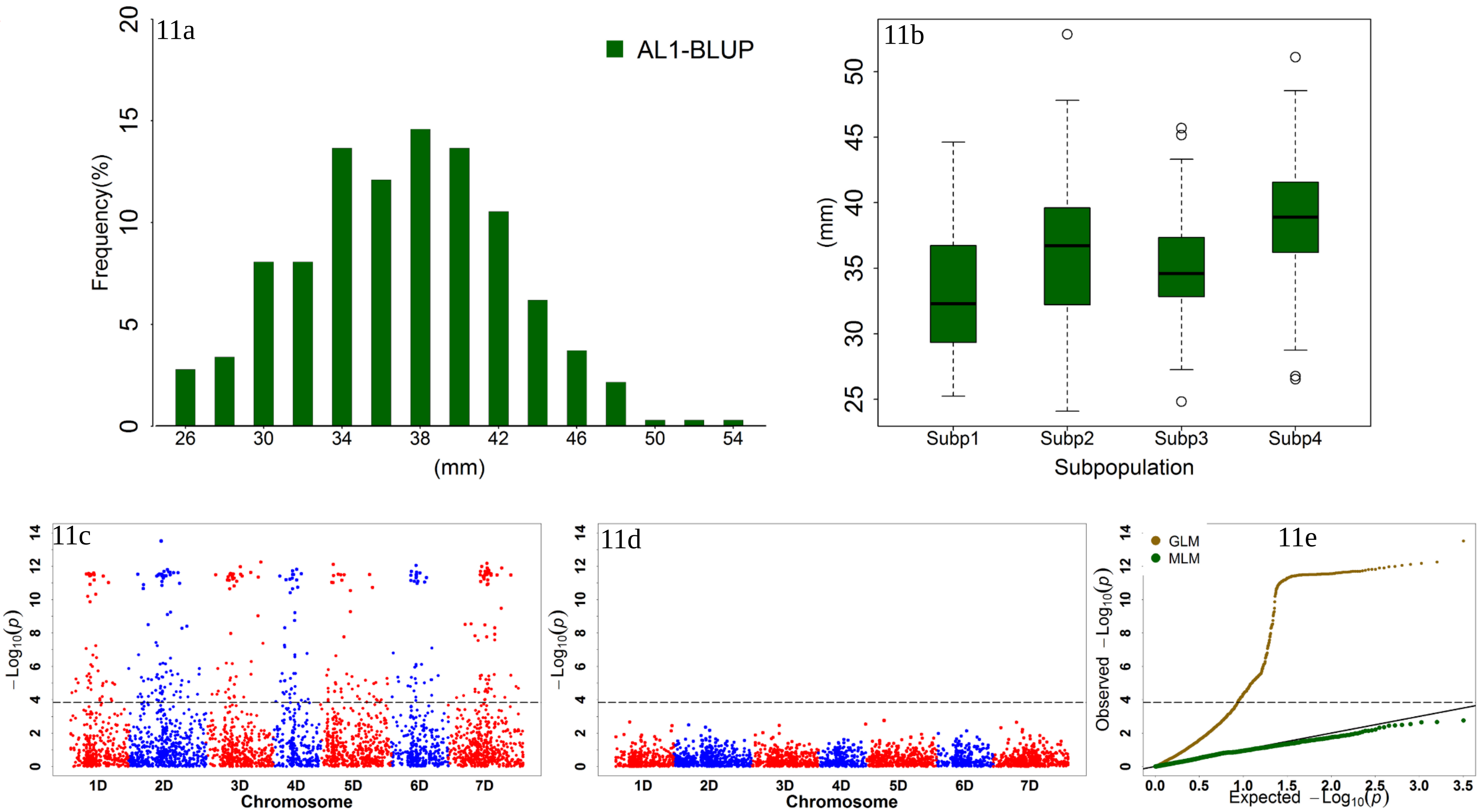

11: Summary of GWAS results for BLUP of awn length 1 (AL1-BLUP). (11a, 11b) Phenotypic BLUP histogram, distribution of each subpopulation. (11c) GLM results for association analysis. (11d) MLM results for association analysis. (11e) Q-Q plots of GLM and MLM.

12

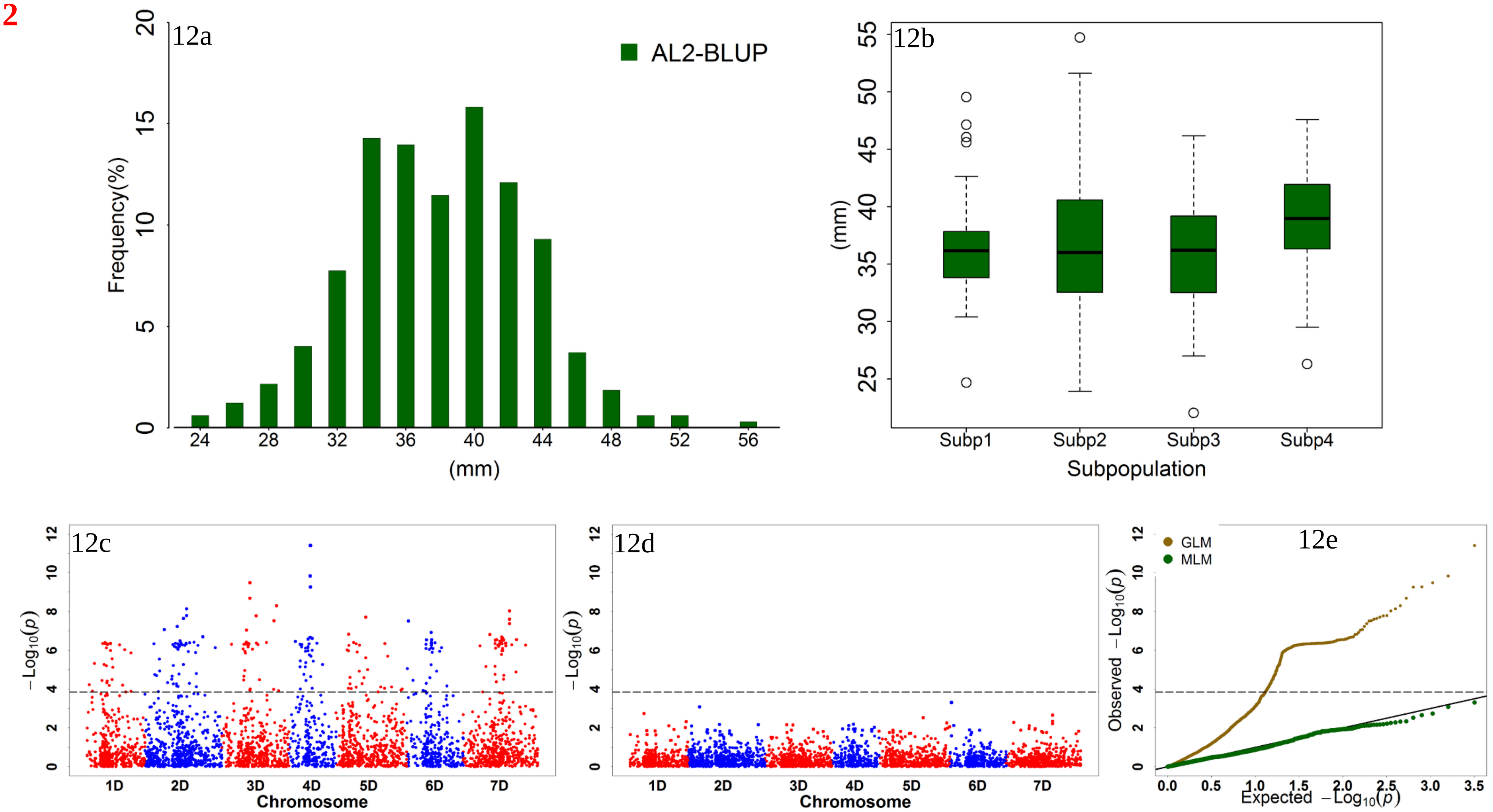

12: Summary of GWAS results for BLUP of awn length 2 (AL2-BLUP). (12a, 12b) Phenotypic BLUP histogram, distribution of each subpopulation. (12c) GLM results for association analysis. (12d) MLM results for association analysis. (12e) Q-Q plots of GLM and MLM.

13

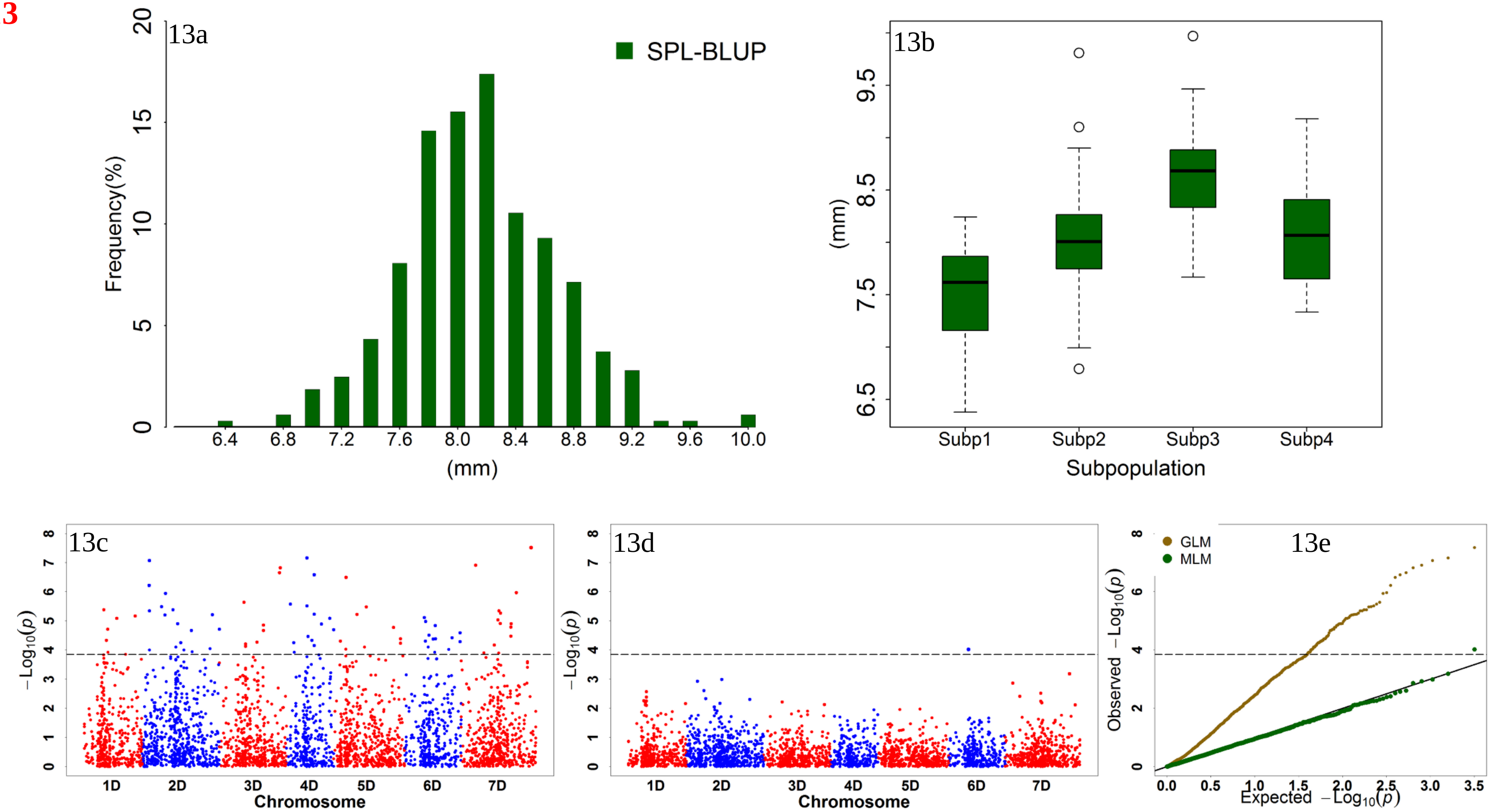

13: Summary of GWAS results for BLUP of spikelet length (SPL-BLUP). (13a, 13b) Phenotypic BLUP histogram, distribution of each subpopulation. (13c) GLM results for association analysis. (13d) MLM results for association analysis. (13e) Q-Q plots of GLM and MLM.

14

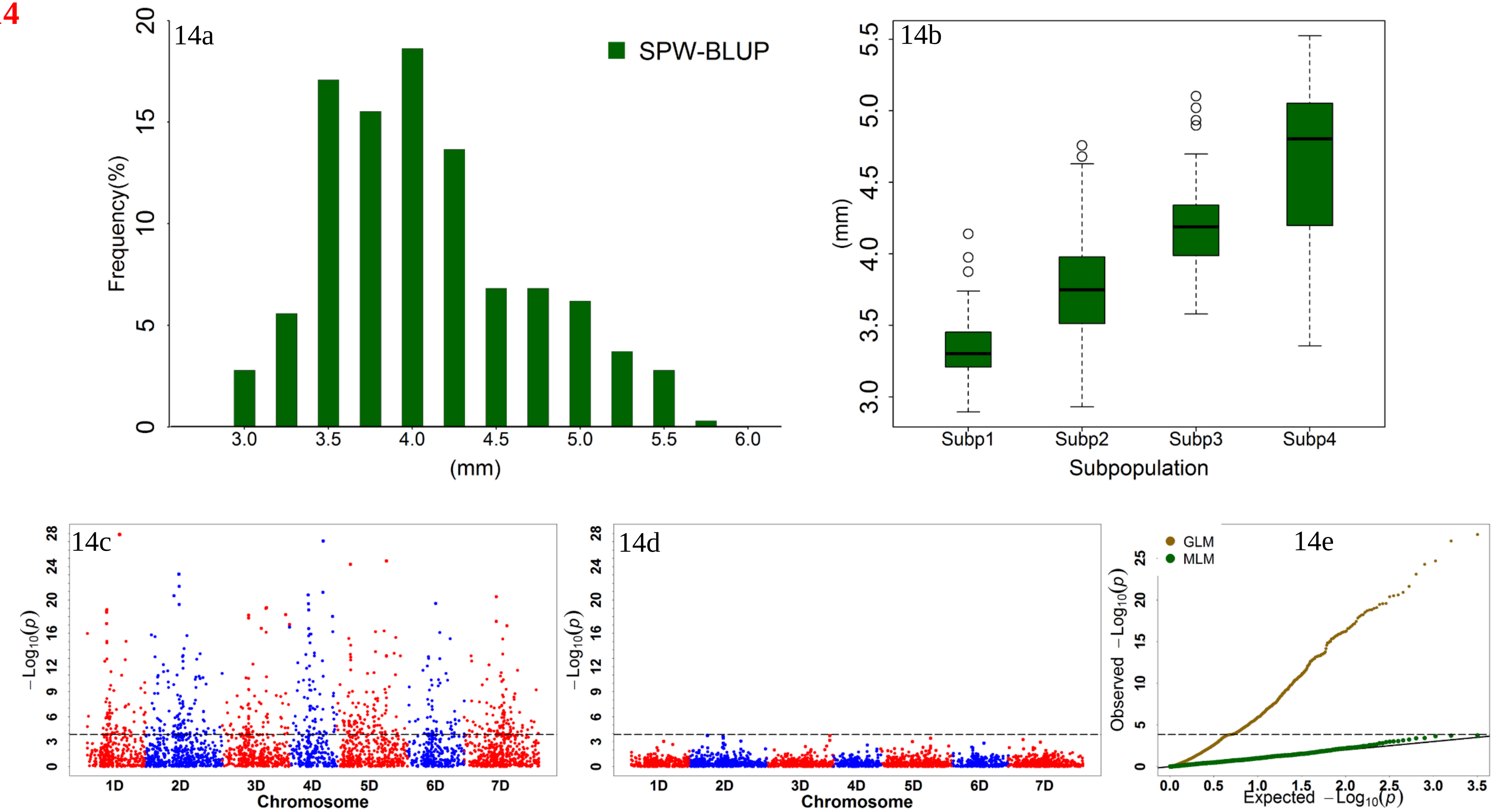

14: Summary of GWAS results for BLUP of spikelet width (SPW-BLUP). (14a, 14b) Phenotypic BLUP histogram, distribution of each subpopulation. (14c) GLM results for association analysis. (14d) MLM results for association analysis. (14e) Q-Q plots of GLM and MLM.

15

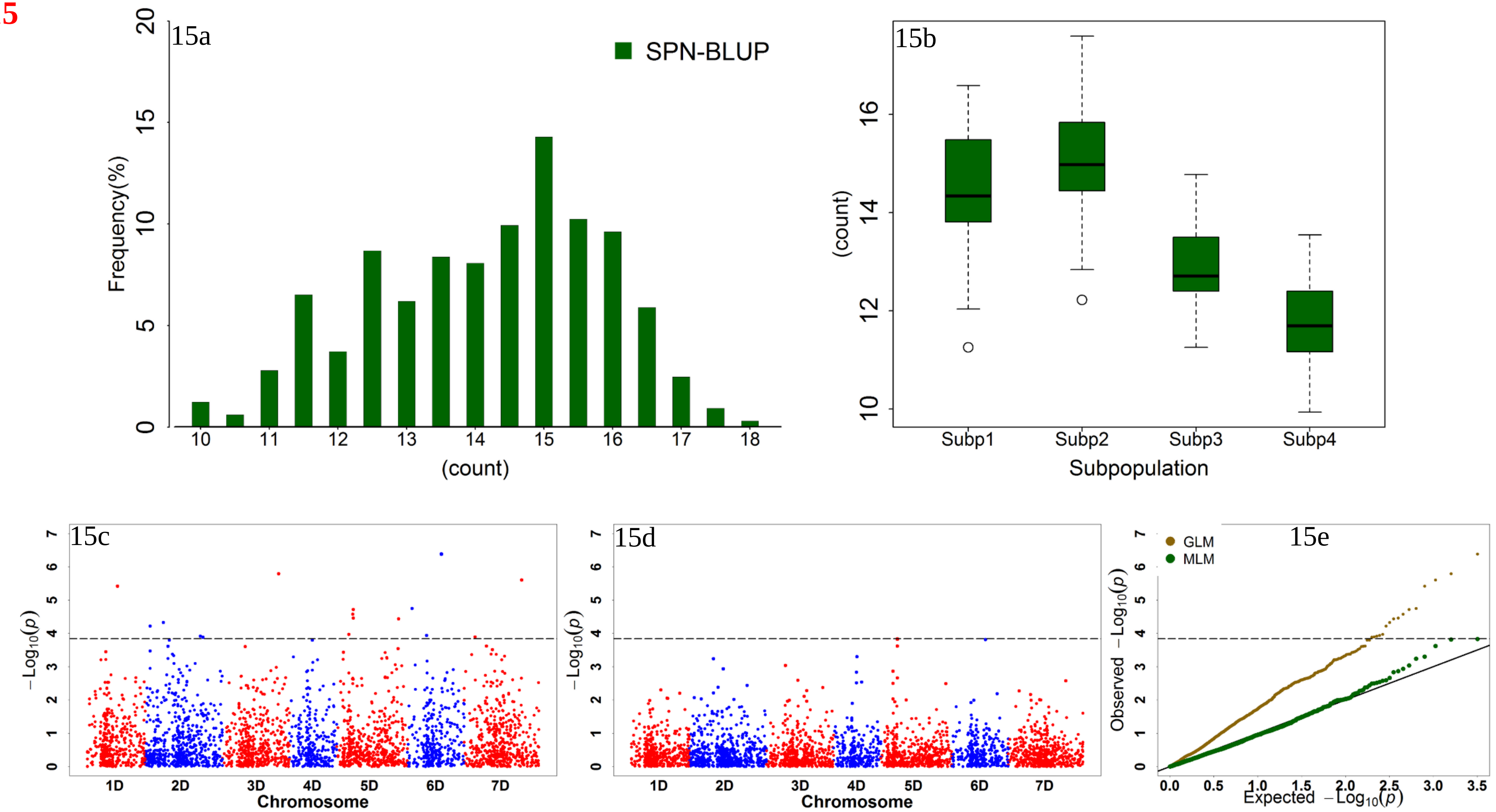

15: Summary of GWAS results for BLUP of spikelet numbers (SPN-BLUP). (15a, 15b) Phenotypic BLUP histogram, distribution of each subpopulation. (15c) GLM results for association analysis. (15d) MLM results for association analysis. (15e) Q-Q plots of GLM and MLM.

16

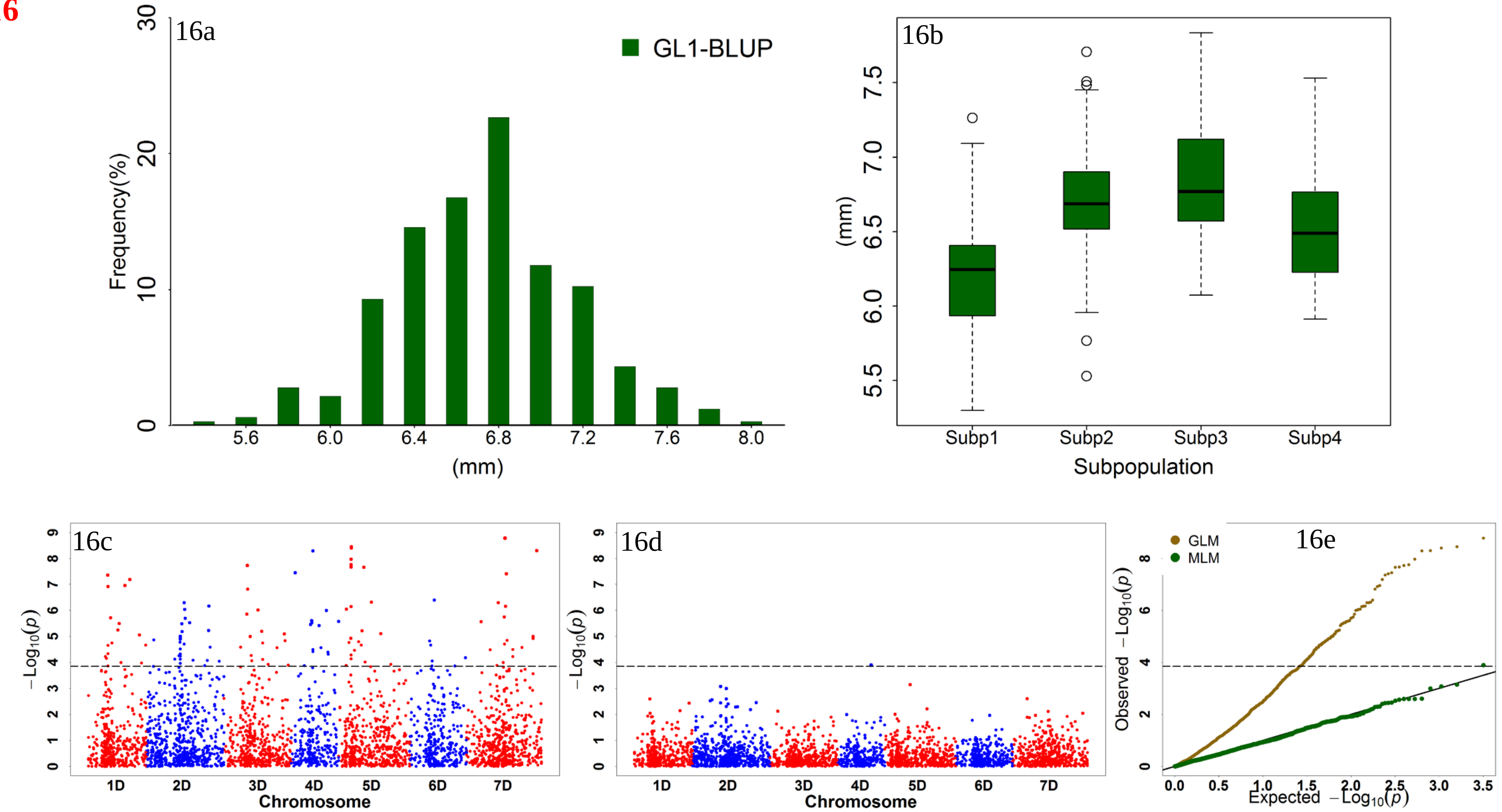

16: Summary of GWAS results for BLUP of glume length 1 (GL1-BLUP). (16a, 16b) Phenotypic BLUP histogram, distribution of each subpopulation. (16c) GLM results for association analysis. (16d) MLM results for association analysis. (16e) Q-Q plots of GLM and MLM.

17

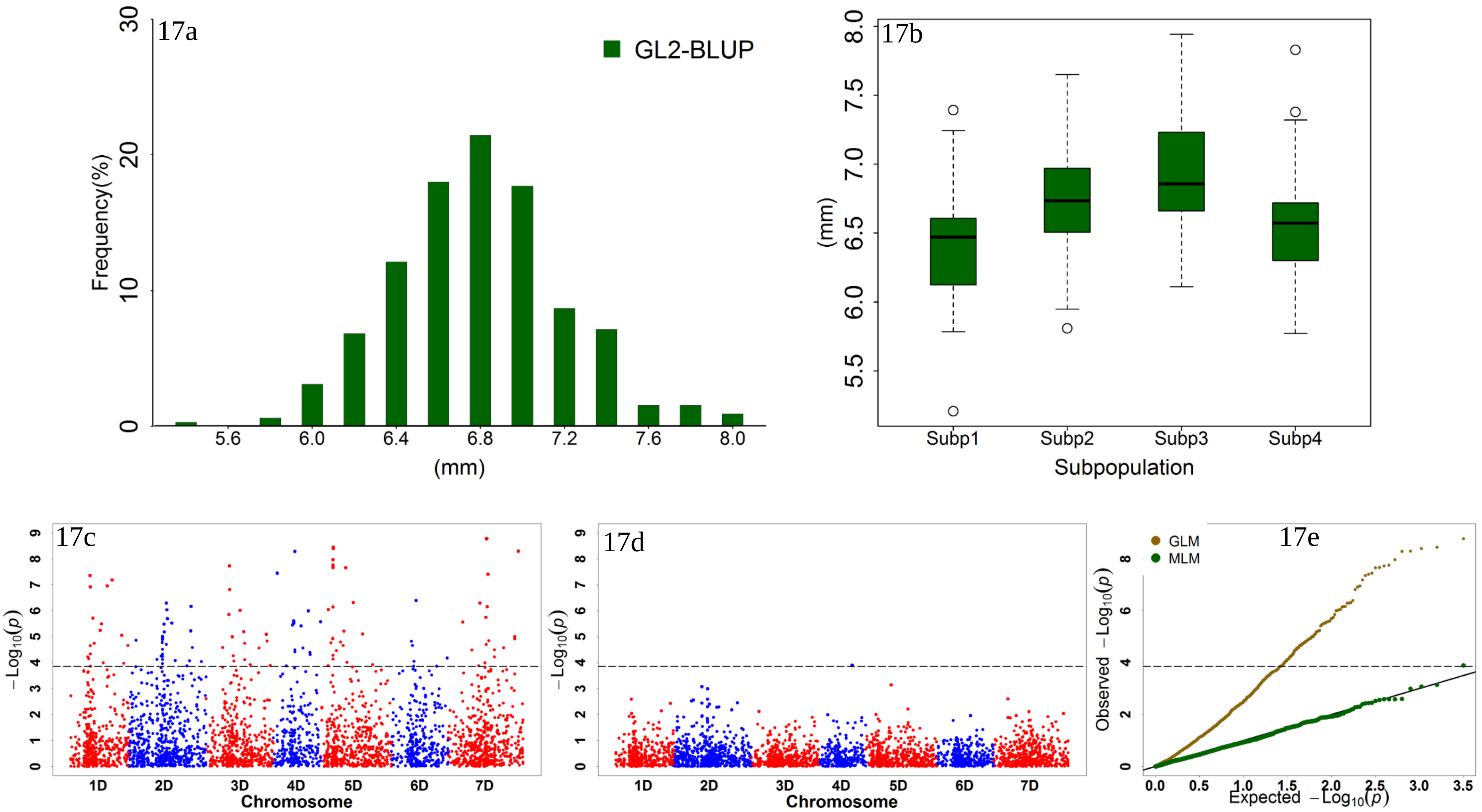

17: Summary of GWAS results for BLUP of glume length 2 (GL2-BLUP). (17a, 17b) Phenotypic BLUP histogram, distribution of each subpopulation. (17c) GLM results for association analysis. (17d) MLM results for association analysis. (17e) Q-Q plots of GLM and MLM.

18

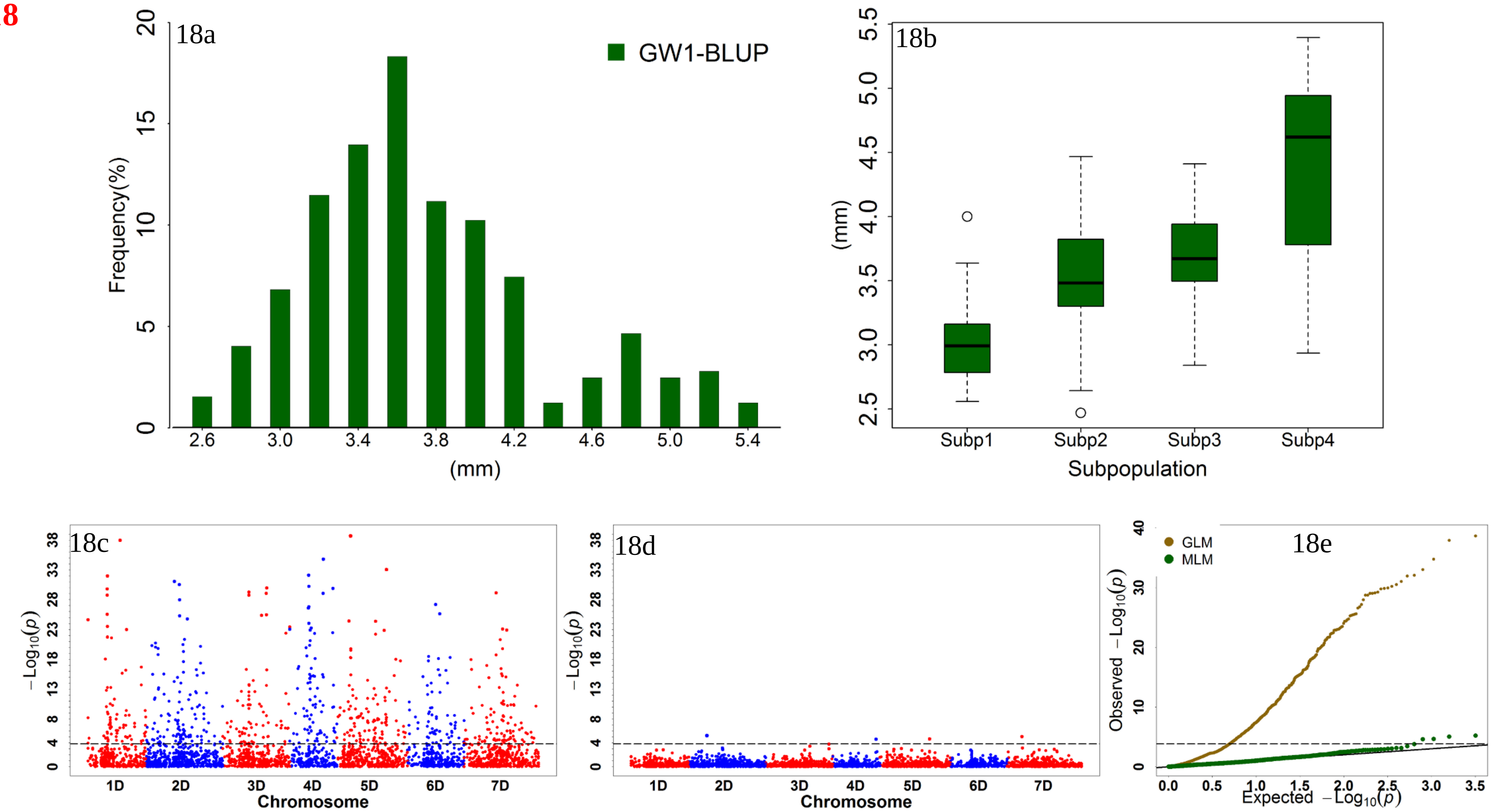

18: Summary of GWAS results for BLUP of glume width 1 (GW1-BLUP). (18a, 18b) Phenotypic BLUP histogram, distribution of each subpopulation. (18c) GLM results for association analysis. (18d) MLM results for association analysis. (18e) Q-Q plots of GLM and MLM.

19

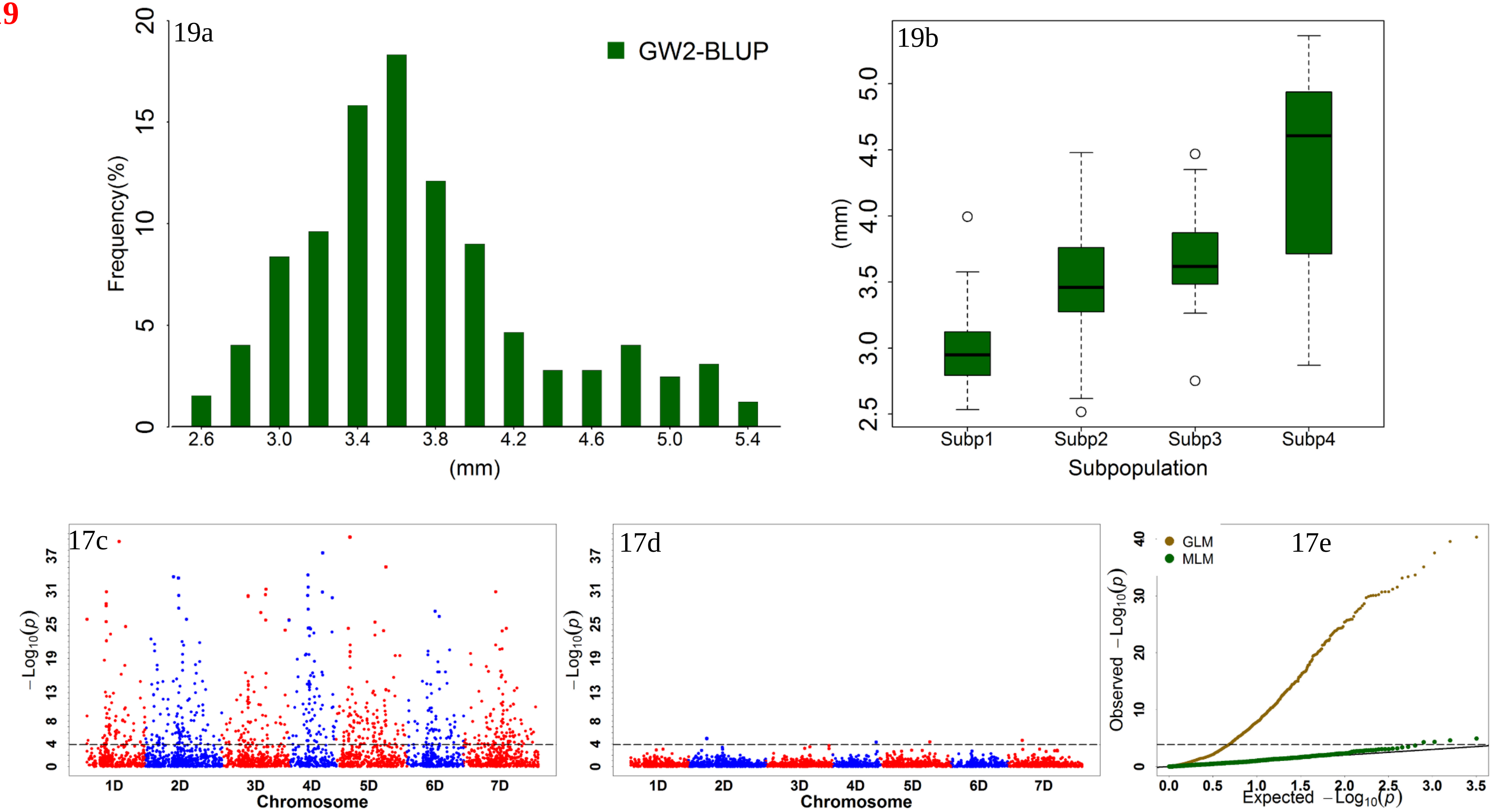

19: Summary of GWAS results for BLUP of glume width 2 (GW2-BLUP). (19a, 19b) Phenotypic BLUP histogram, distribution of each subpopulation. (19c) GLM results for association analysis. (19d) MLM results for association analysis. (19e) Q-Q plots of GLM and MLM.

20

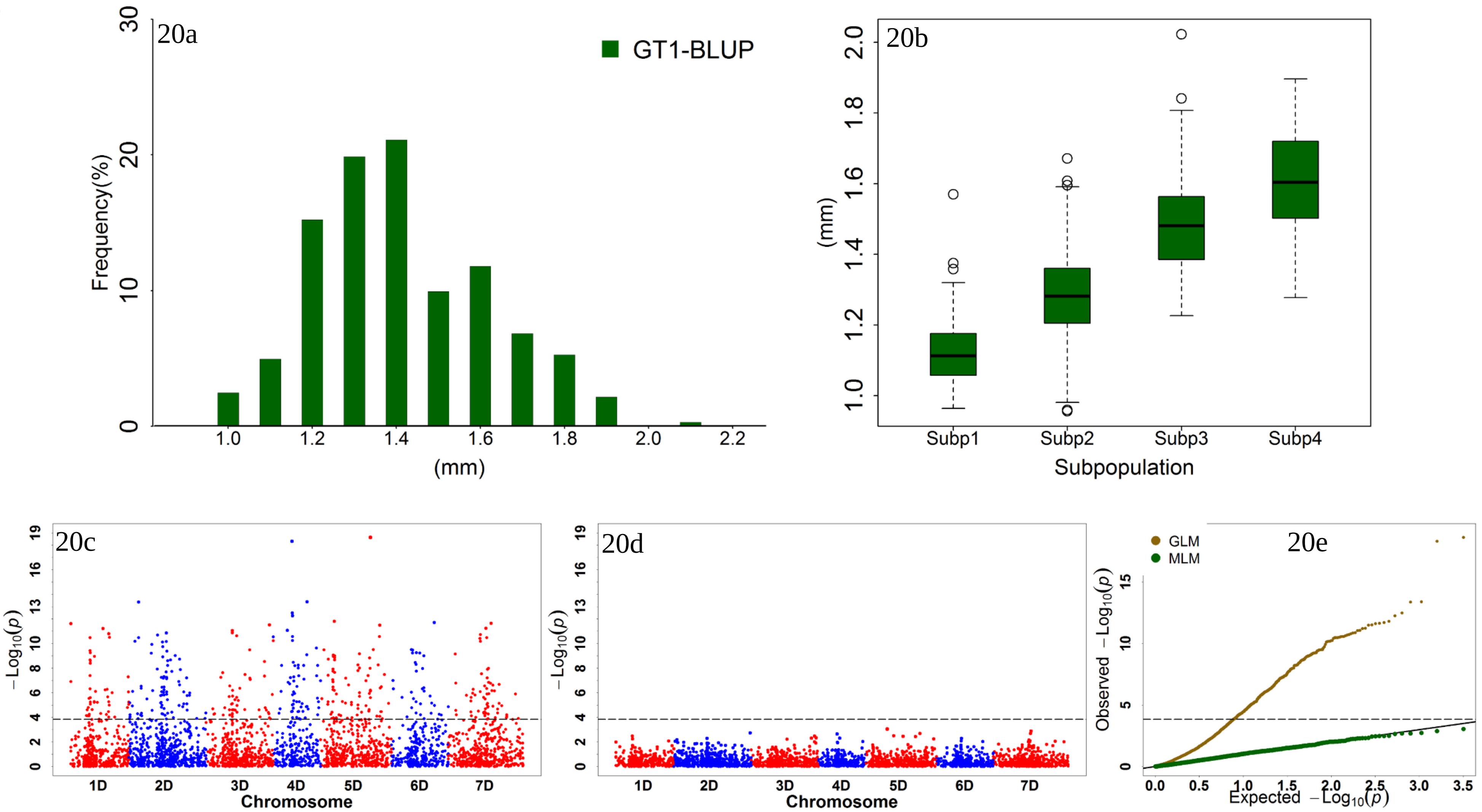

20: Summary of GWAS results for BLUP of glume thickness 1 (GT1-BLUP). (20a, 20b) Phenotypic BLUP histogram, distribution of each subpopulation. (20c) GLM results for association analysis. (20d) MLM results for association analysis. (20e) Q-Q plots of GLM and MLM.

21

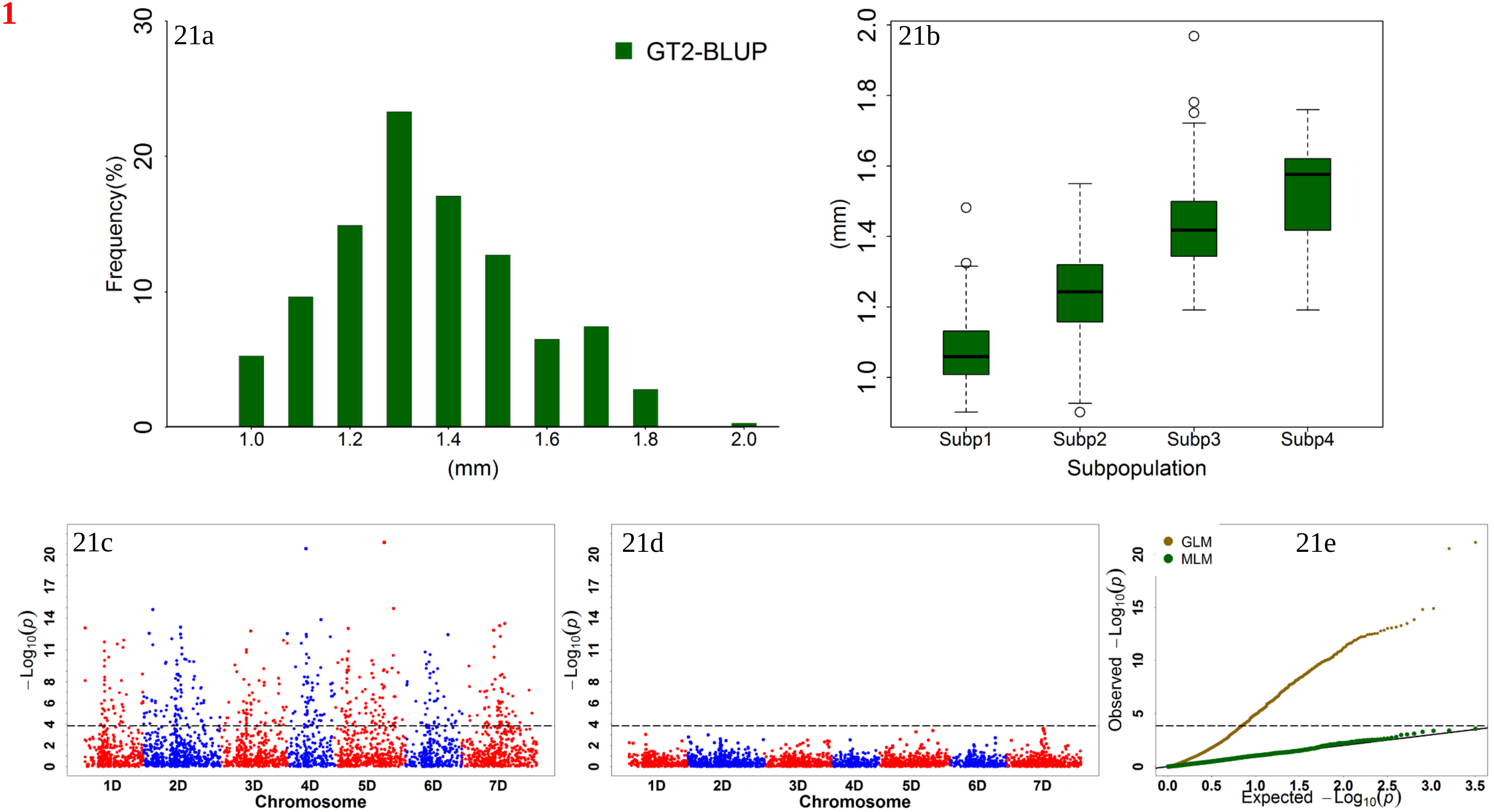

21: Summary of GWAS results for BLUP of glume thickness 2 (GT2-BLUP). (21a, 21b) Phenotypic BLUP histogram, distribution of each subpopulation. (21c) GLM results for association analysis. (21d) MLM results for association analysis. (21e) Q-Q plots of GLM and MLM.

22

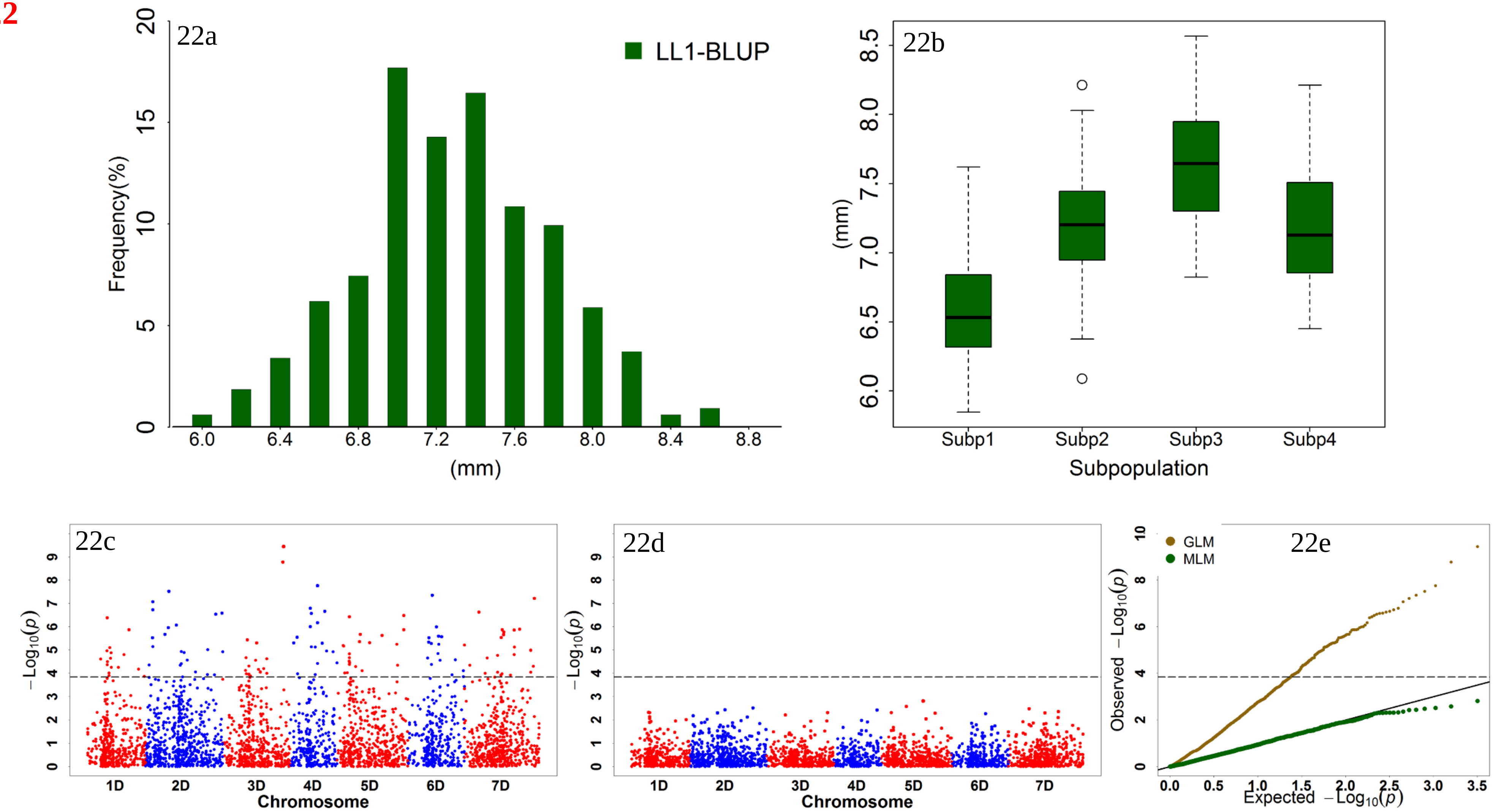

22: Summary of GWAS results for BLUP of lemma length 1 (LL1-BLUP). (22a, 22b) Phenotypic BLUP histogram, distribution of each subpopulation. (22c) GLM results for association analysis. (22d) MLM results for association analysis. (22e) Q-Q plots of GLM and MLM.

23

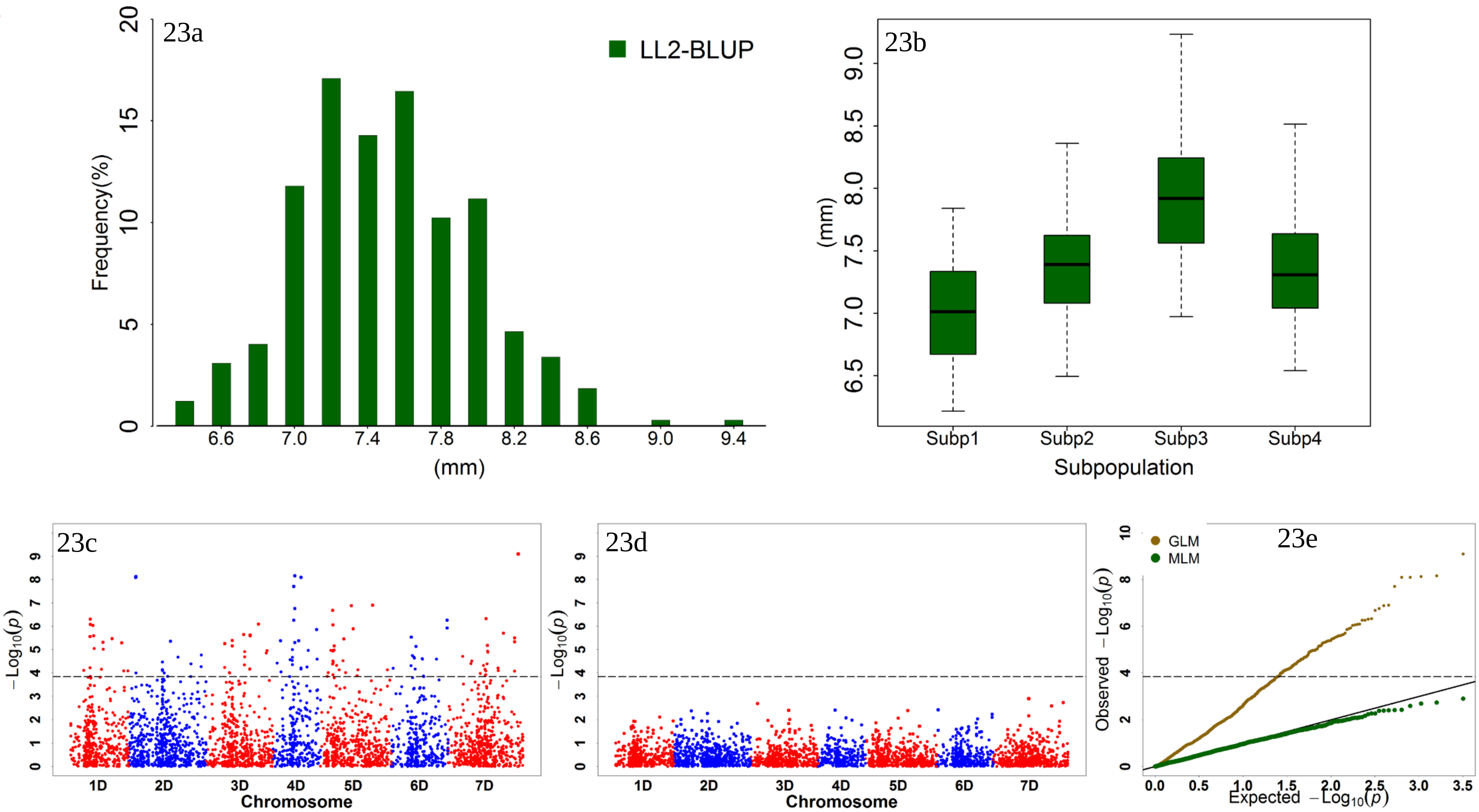

23: Summary of GWAS results for BLUP of lemma length 2 (LL2-BLUP). (23a, 23b) Phenotypic BLUP histogram, distribution of each subpopulation. (23c) GLM results for association analysis. (23d) MLM results for association analysis. (23e) Q-Q plots of GLM and MLM.

24

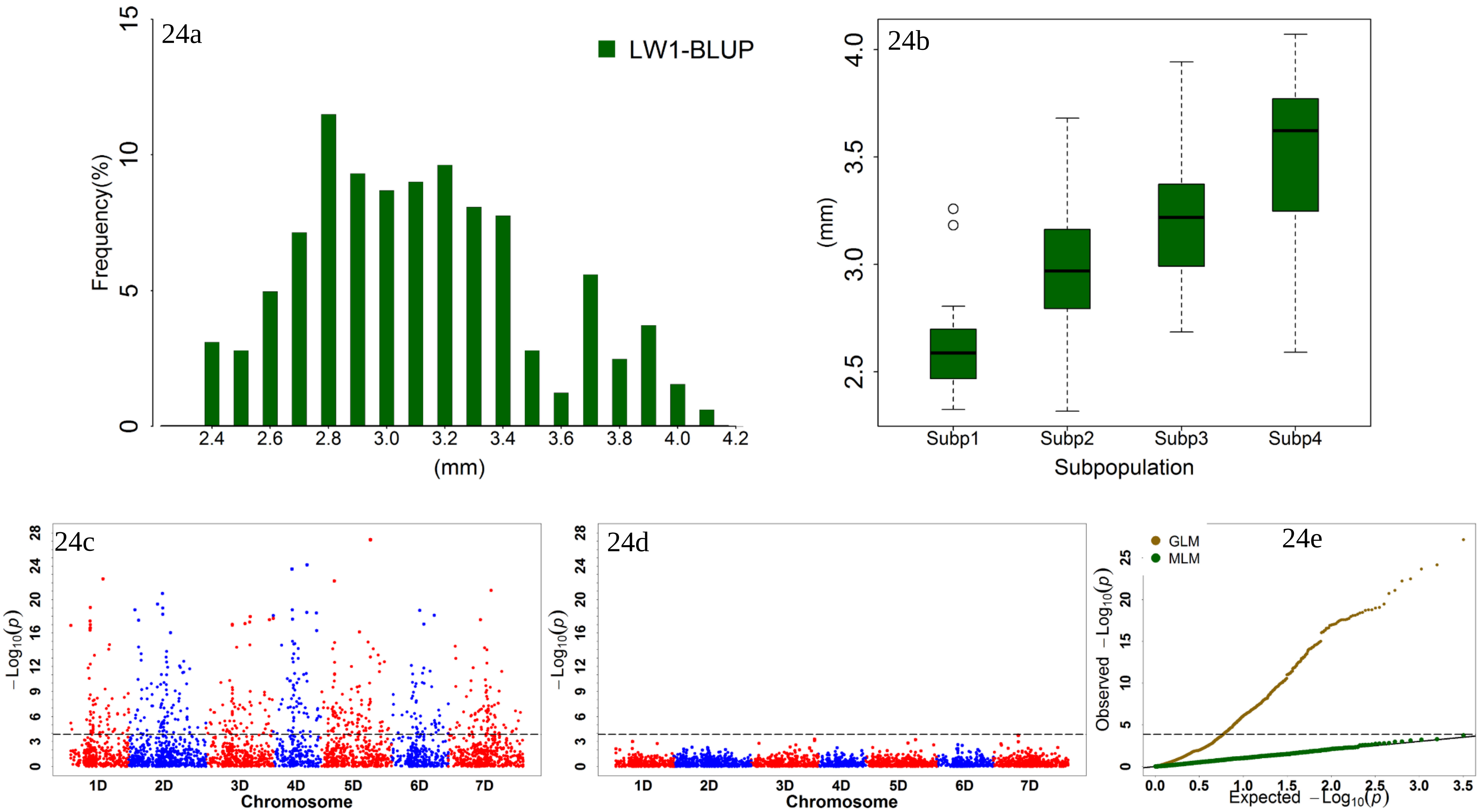

24: Summary of GWAS results for BLUP of lemma width 1 (LW1-BLUP). (24a, 24b) Phenotypic BLUP histogram, distribution of each subpopulation. (24c) GLM results for association analysis. (24d) MLM results for association analysis. (24e) Q-Q plots of GLM and MLM.

25

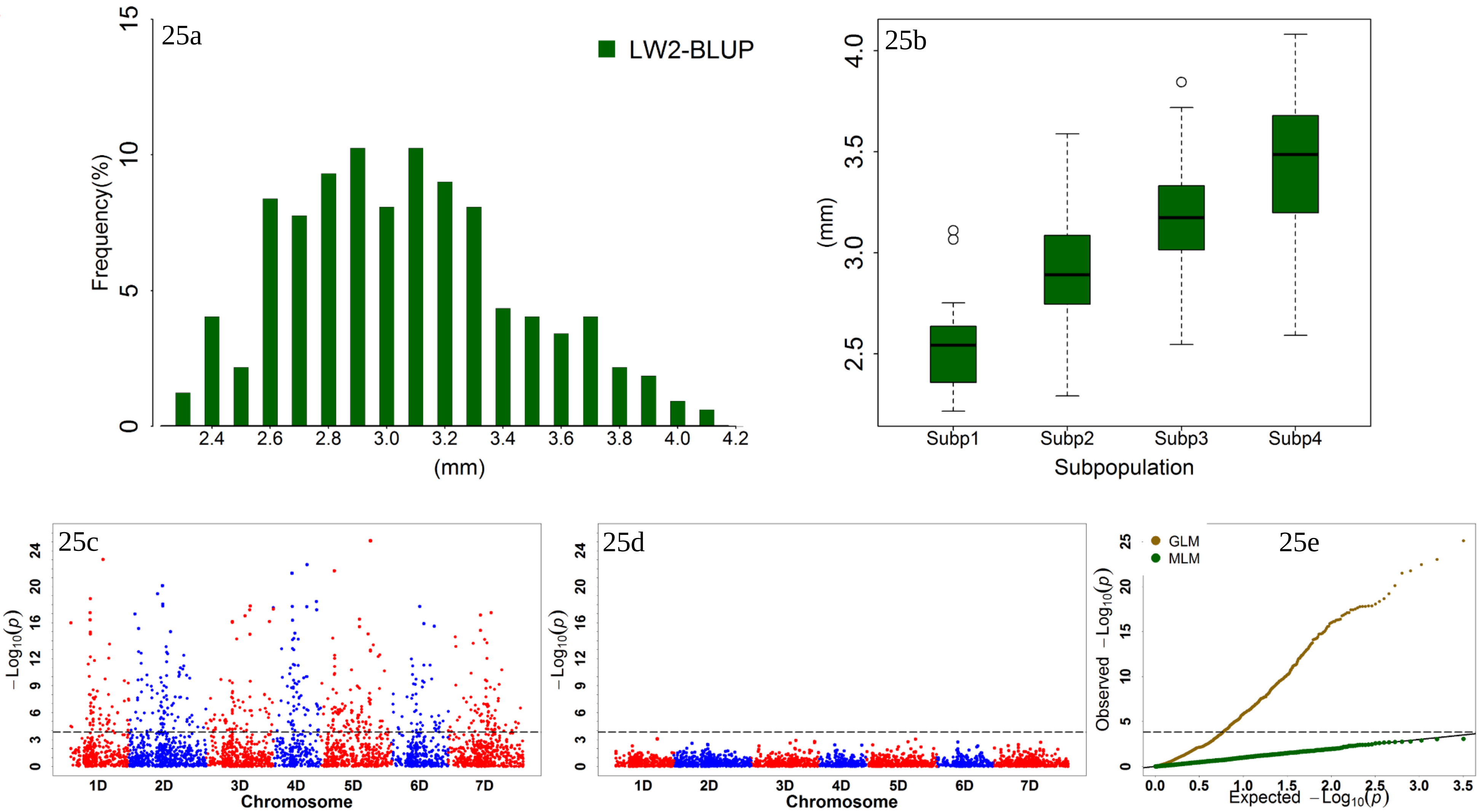

25: Summary of GWAS results for BLUP of lemma width 2 (LW2-BLUP). (25a, 25b) Phenotypic BLUP histogram, distribution of each subpopulation. (25c) GLM results for association analysis. (25d) MLM results for association analysis. (25e) Q-Q plots of GLM and MLM.

26

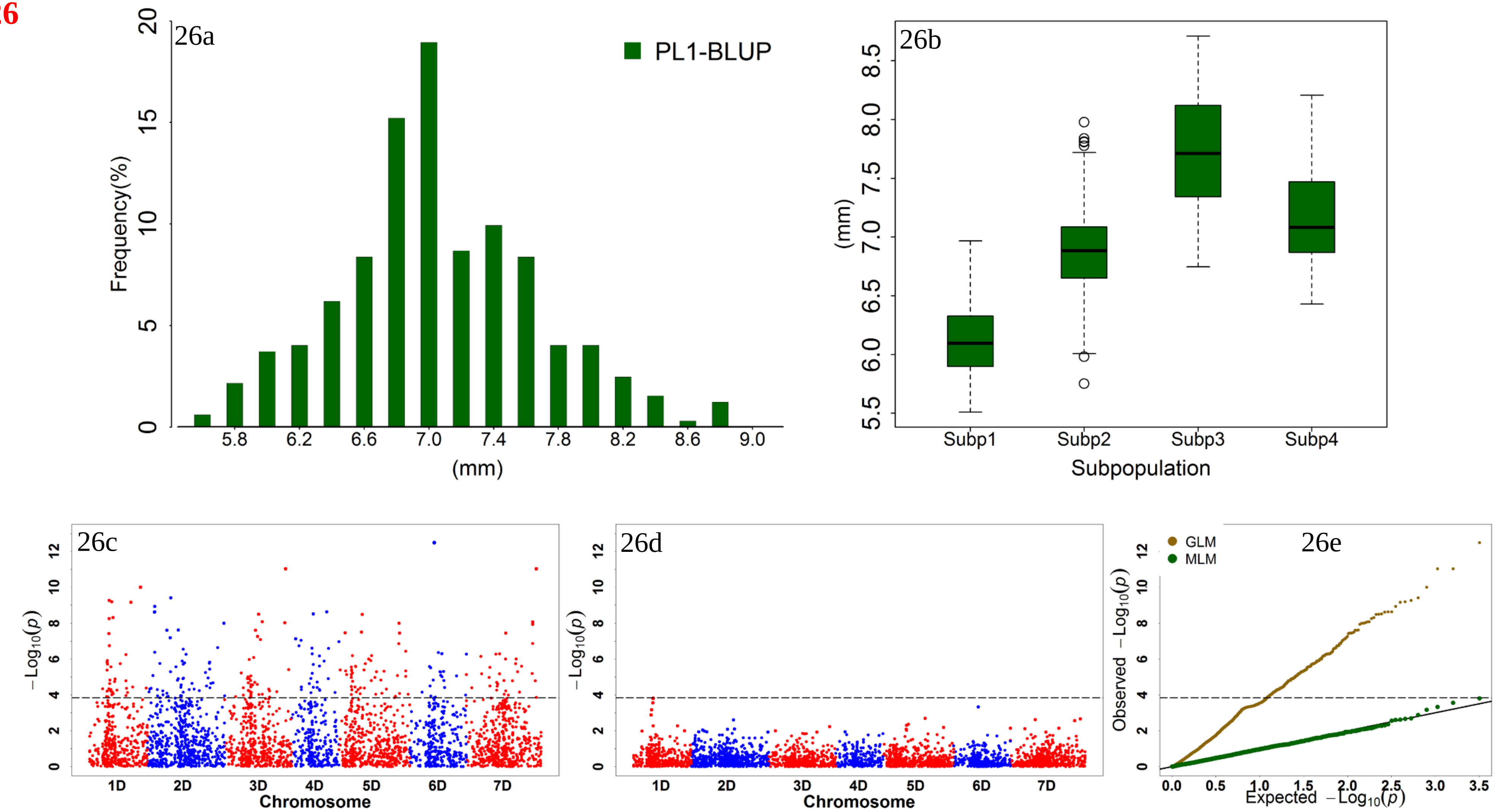

26: Summary of GWAS results for BLUP of palea length 1 (PL1-BLUP). (26a, 26b) Phenotypic BLUP histogram, distribution of each subpopulation. (26c) GLM results for association analysis. (26d) MLM results for association analysis. (26e) Q-Q plots of GLM and MLM.

27

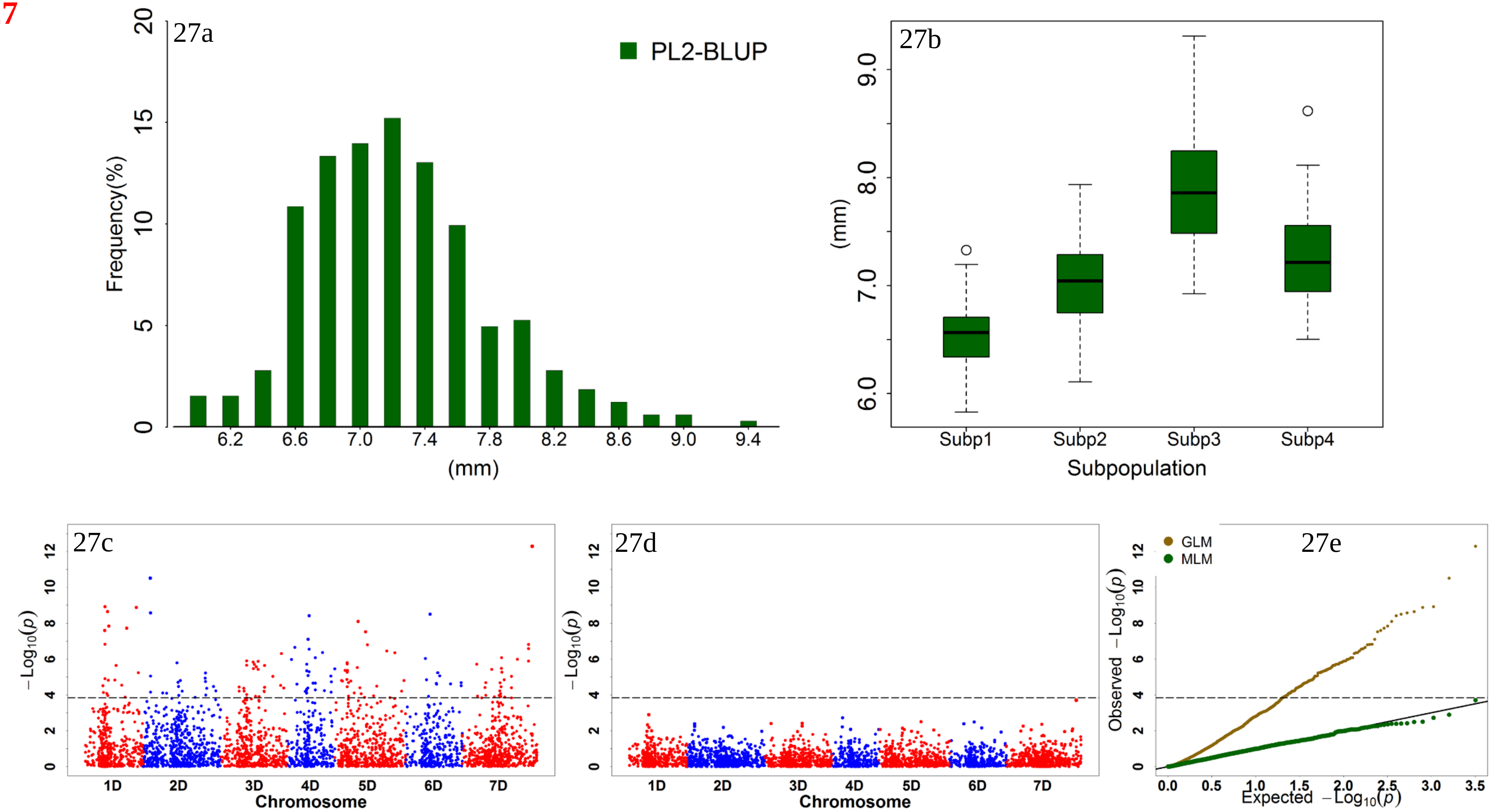

27: Summary of GWAS results for BLUP of palea length 2 (PL2-BLUP). (27a, 27b) Phenotypic BLUP histogram, distribution of each subpopulation. (27c) GLM results for association analysis. (27d) MLM results for association analysis. (27e) Q-Q plots of GLM and MLM.

28

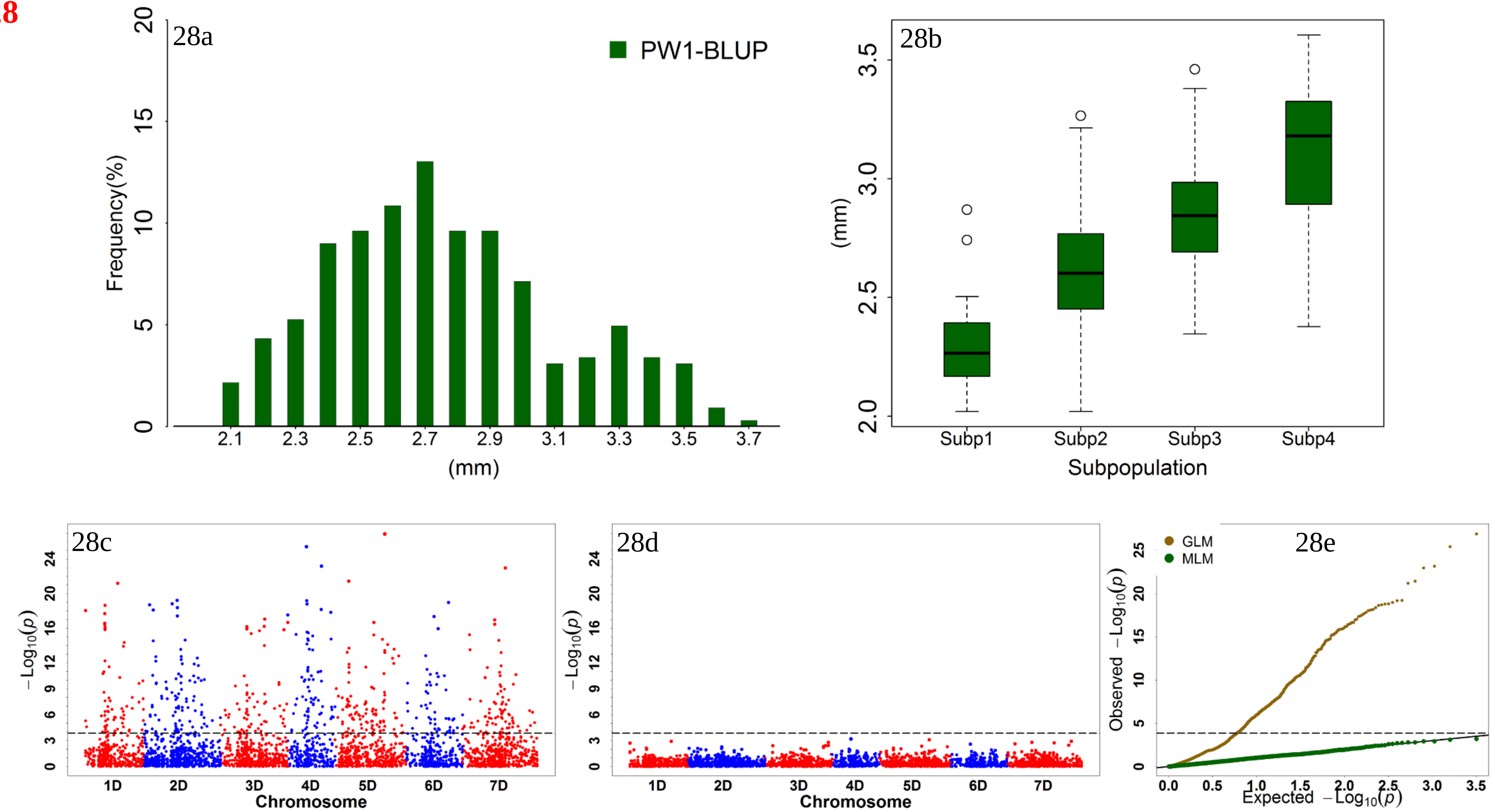

28: Summary of GWAS results for BLUP of palea width 1 (PW1-BLUP). (28a, 28b) Phenotypic BLUP histogram, distribution of each subpopulation. (28c) GLM results for association analysis. (28d) MLM results for association analysis. (28e) Q-Q plots of GLM and MLM.

29

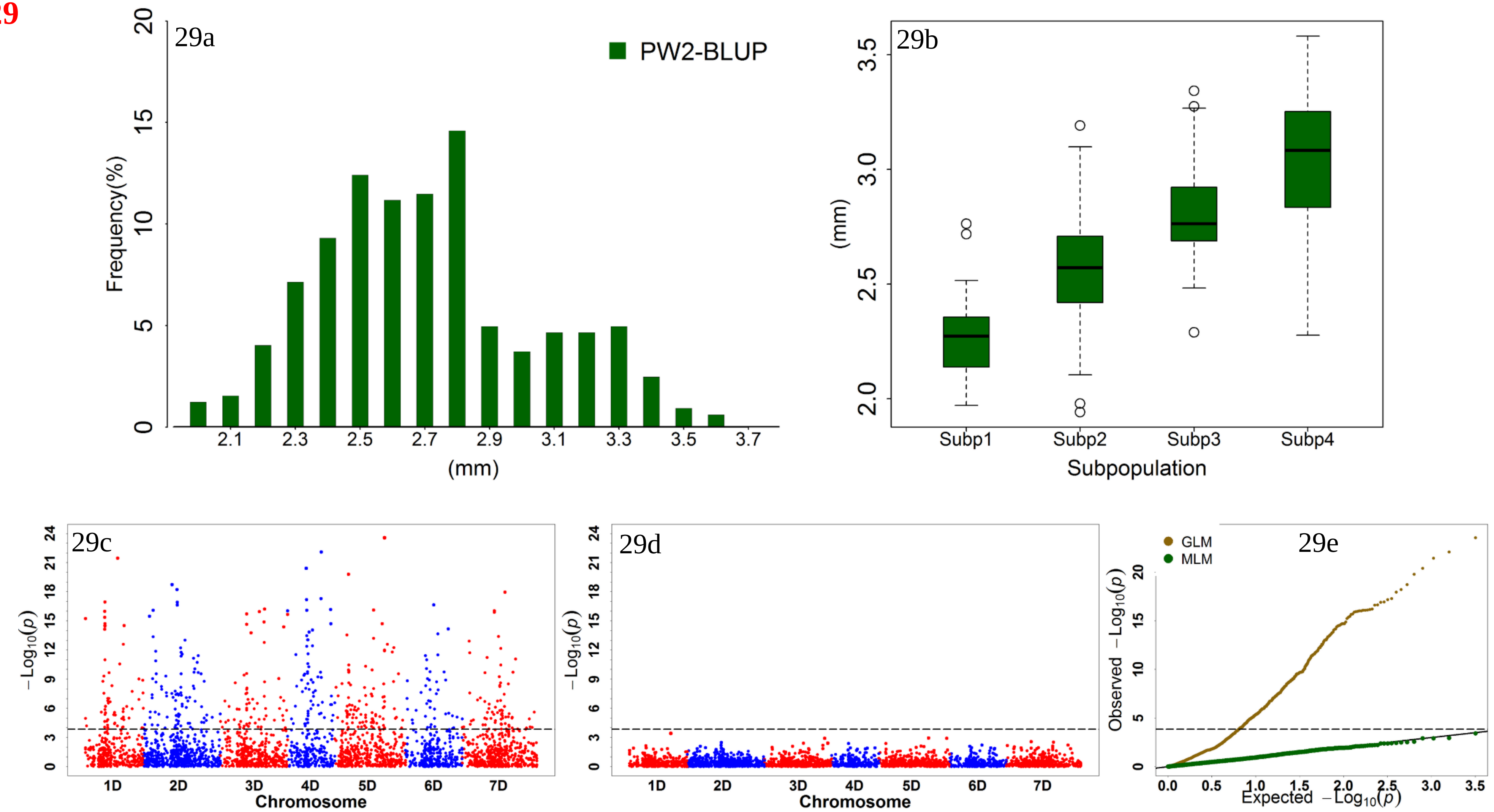

29: Summary of GWAS results for BLUP of palea width 2 (PW2-BLUP). (29a, 29b) Phenotypic BLUP histogram, distribution of each subpopulation. (29c) GLM results for association analysis. (29d) MLM results for association analysis. (29e) Q-Q plots of GLM and MLM.

Table S1. Heritability estimates for the investigated 29 morphological traits.

Trait	Heritability	Trait	Heritability	Trait	Heritability	Trait	Heritability
PH	0.58	LN	0.27	GL2	0.83	LW2	0.88
SL	0.64	SNN	0.65	GW1	0.94	PL1	0.87
IL1	0.81	AL1	0.73	GW2	0.94	PL2	0.79
IL2	0.86	AL2	0.76	GT1	0.83	PW1	0.84
IL3	0.85	SPL	0.81	GT2	0.84	PW2	0.83
IL4	0.82	SPW	0.9	LL1	0.82		
FL	0.6	SPN	0.86	LL2	0.75		
FW	0.64	GL1	0.84	LW1	0.88		

PH: plant height; SL: spike length; IL1: internode length1; IL2: internode length 2; IL3: internode length 3; IL4: internode length 4; FL: flag leaf length; FW: flag leaf width; LN: leaf numbers; SNN: stem node numbers; AL1: awn length1; AL2: awn length 2; SPL: spikelet length; SPW: spikelet width; SPN: spikelet numbers; GL1: glume length1; GL2: glume length 2; GW1: glume width 1; GW2: glume width 2; GT1: glume thickness 1; GT2: glume thickness 2; LL1: lemma length 1; LL2: lemma length 2; LW1: lemma width 1; LW2: lemma width 2; PL1: palea length 1; PL2: palea length 2; PW1: palea width 1; PW2: palea width 2.

Table S2. Correlation coefficients (r) for the tested traits between year 2012 and 2013.

**Trait**	***r***	**Trait**	***r***	**Trait**	***r***	**Trait**	***r***
PH	0.430**	LN	0.174**	GL2	0.736**	LW2	0.803**
SL	0.502**	SNN	0.530**	GW1	0.891**	PL1	0.793**
IL1	0.718**	AL1	0.599**	GW2	0.891**	PL2	0.676**
IL2	0.776**	AL2	0.632**	GT1	0.724**	PW1	0.737**
IL3	0.760**	SPL	0.713**	GT2	0.739**	PW2	0.718**
IL4	0.727**	SPW	0.830**	LL1	0.727**		
FL	0.455**	SPN	0.778**	LL2	0.627**		
FW	0.501**	GL1	0.749**	LW1	0.796**		

PH: plant height; SL: spike length; IL1: internode length1; IL2: internode length 2; IL3: internode length 3; IL4: internode length 4; FL: flag leaf length; FW: flag leaf width; LN: leaf numbers; SNN: stem node numbers; AL1: awn length1; AL2: awn length 2; SPL: spikelet length; SPW: spikelet width; SPN: spikelet numbers; GL1: glume length1; GL2: glume length 2; GW1: glume width 1; GW2: glume width 2; GT1: glume thickness 1; GT2: glume thickness 2; LL1: lemma length 1; LL2: lemma length 2; LW1: lemma width 1; LW2: lemma width 2; PL1: palea length 1; PL2: palea length 2; PW1: palea width 1; PW2: palea width 2.

**, significant at $P<0.01$.

Table S4. Classification of 322 accessions of *Aegilops tauschii* according to discriminant function analysis.

Name			Division by discriminant analysis				Total
			**ssp. *taschii*_I**	**ssp. *taschii*_II**	**ssp. *strangulata*_I**	**ssp. *strangulata*_II**	
Original	Count	ssp. *taschii*_I	46	2	0	1	49
		ssp. *taschii*_II	14	139	0	2	155
		ssp. *strangulata*_I	1	0	48	2	51
		ssp. *strangulata*_II	0	0	9	58	67
	Percent (%)	ssp. *taschii*_I	93.9	4.1	0	2	100
		ssp. *taschii*_II	9	89.7	0	1.3	100
		ssp. *strangulata*_I	2	0	94.1	3.9	100
		ssp. *strangulata*_II	0	0	13.4	86.6	100

Table S6. Summary of cluster analysis in 322 Aegilops tauschii accessions.

**Cluster**	**Accessions**	**Origins**	**ssp. *taschii*_I**	**ssp. *taschii*_II**	**ssp. *strangulata*_I**	**ssp. *strangulata*_II**
1	113	13	4	106	3	0
2	78	16	43	23	5	7
3	44	3	0	1	1	42
4	87	13	2	25	42	18
